# Supplementary material for: Mechanistic insights into the three steps of poly(ADP-ribosylation) reversal
Source: Nat Commun. 2021 Jul 28;12:4581. doi: 10.1038/s41467-021-24723-3 (PMC8319183; doi:10.1038/s41467-021-24723-3)
Supplement: Supplementary file 2 — Reporting Summary [file 41467_2021_24723_MOESM2_ESM.pdf]

nature portfolio

Corresponding author(s): Ivan Ahel

Last updated by author(s): Jun 28, 2021

Reporting Summary

Nature Portfolio wishes to improve the reproducibility of the work that we publish. This form provides structure for consistency and transparency in reporting. For further information on Nature Portfolio policies, see our Editorial Policies and the Editorial Policy Checklist.

Statistics

For all statistical analyses, confirm that the following items are present in the figure legend, table legend, main text, or Methods section.

n/a	Confirmed	
☐	☒	The exact sample size (*n*) for each experimental group/condition, given as a discrete number and unit of measurement
☐	☒	A statement on whether measurements were taken from distinct samples or whether the same sample was measured repeatedly
☐	☒	The statistical test(s) used AND whether they are one- or two-sided<br>*Only common tests should be described solely by name; describe more complex techniques in the Methods section.*
☒	☐	A description of all covariates tested
☒	☐	A description of any assumptions or corrections, such as tests of normality and adjustment for multiple comparisons
☐	☒	A full description of the statistical parameters including central tendency (e.g. means) or other basic estimates (e.g. regression coefficient) AND variation (e.g. standard deviation) or associated estimates of uncertainty (e.g. confidence intervals)
☐	☒	For null hypothesis testing, the test statistic (e.g. *F*, *t*, *r*) with confidence intervals, effect sizes, degrees of freedom and *P* value noted<br>*Give P values as exact values whenever suitable.*
☒	☐	For Bayesian analysis, information on the choice of priors and Markov chain Monte Carlo settings
☒	☐	For hierarchical and complex designs, identification of the appropriate level for tests and full reporting of outcomes
☒	☐	Estimates of effect sizes (e.g. Cohen's *d*, Pearson's *r*), indicating how they were calculated

Our web collection on statistics for biologists contains articles on many of the points above.

Software and code

Policy information about availability of computer code

Data collection	Generic Data Acquisition software (GDA, v8.26, Diamond Light Source)<br>Xcalibur (v4.2, Thermo Scientific)<br>SoftMax Pro (v5.4, Molecular Devices)
Data analysis	Publicly available software:<br>Statistical analysis and graph representation: Prism (v9.1, GraphPad)<br>Mass spectra analysis: MNova (v13, Mestrelab Research)<br>Data reduction: Xia (v0.3.8)<br>Molecular replacement: Phaser (v2.7)<br>Data refinement: REFMAC5<br>Manual model building: Coot (v0.8.9)<br>Software suits: CCP4i2 (v7), Phenix (v1.18)<br>Structure validation: MolProbity (v4.5, Duke University)<br>Structure representation and modeling: PyMOL (v2.3, Schrödinger, LLC), Chimera (v1.15, UCSF), YASARA (v21.2, YASARA Biosciences GmbH)

For manuscripts utilizing custom algorithms or software that are central to the research but not yet described in published literature, software must be made available to editors and reviewers. We strongly encourage code deposition in a community repository (e.g. GitHub). See the Nature Portfolio guidelines for submitting code & software for further information.

Data

Policy information about availability of data

All manuscripts must include a data availability statement. This statement should provide the following information, where applicable:

- Accession codes, unique identifiers, or web links for publicly available datasets
- A description of any restrictions on data availability
- For clinical datasets or third party data, please ensure that the statement adheres to our policy

The atomic coordinates and structure factors for the hARH3 E41A:H2BS7mar, hARH3 E41A:dimer, hARH3 E41A:α-NAD+, and LchARH3:meADPr structures reported in this paper have been deposited in the RCSB Protein Data Bank (www.rcsb.org) under accession codes 7AKS, 7AKR, 7ARW, and 7AQM, respectively (see Methods and Supplementary Table 2). Earlier deposited structural data used in this study are available in the RCSB Protein Data Bank (www.rcsb.org) under accession codes 2FOZ (hARH3 apo form), 5A7R (PARG:dimer), 6D36 (hARH3:ADPr), 6HGZ (LchARH3:ADPr), and 6HH3 (LchARH3:ADP-HPD). The somatic mutation data used in this study are available in the COSMIC database (https://cancer.sanger.ac.uk/cosmic) under accession codes COSM5033871 (D34G), COSM5992851 (T76R), COSM3727906 (S185P), COSM83890 (L186V), and COSM6262998 (G270C) (Supplementary Table 3). Further information and requests for resources and reagents should be directed to and will be fulfilled by the corresponding authors Ivan Ahel (ivan.ahel@path.ox.ac.uk) and Dmitri V. Filippov (filippov@lic.leidenuniv.nl). Source data are provided with this paper.

Field-specific reporting

Please select the one below that is the best fit for your research. If you are not sure, read the appropriate sections before making your selection.

☒ Life sciences ☐ Behavioural & social sciences ☐ Ecological, evolutionary & environmental sciences

For a reference copy of the document with all sections, see nature.com/documents/nr-reporting-summary-flat.pdf

Life sciences study design

All studies must disclose on these points even when the disclosure is negative.

Sample size	No statistical methods were used to predetermine sample size. For crystallographic data, data were collected from single crystals and resolution limits were determined using data completeness and statistical parameters (incl. cc1/2, R values and I/sigma(I)). For statistical analysis experiments were performed in triplicates to ensure consistence within the experiment.
Data exclusions	No data were excluded.
Replication	For statistical analysis, experiments were performed as three biological replicates measured in triplicates, precise replicate numbers and statistical analysis methods are indicated in the figure legends. All attempts at replication were successful. For in vivo analysis of the branch frequency (R2-Ado/R-Ado ratio) the experiment was performed as biological quadruplicate.
Randomization	Randomization is not applicable as there are no groups to be allocated.
Blinding	Blinding is not applicable as there are no groups to be allocated.

Reporting for specific materials, systems and methods

We require information from authors about some types of materials, experimental systems and methods used in many studies. Here, indicate whether each material, system or method listed is relevant to your study. If you are not sure if a list item applies to your research, read the appropriate section before selecting a response.

Materials & experimental systems

n/a	Involved in the study	
☐	☒	Antibodies
☐	☒	Eukaryotic cell lines
☒	☐	Palaeontology and archaeology
☒	☐	Animals and other organisms
☒	☐	Human research participants
☒	☐	Clinical data
☒	☐	Dual use research of concern

Methods

n/a	Involved in the study	
☒	☐	ChIP-seq
☒	☐	Flow cytometry
☒	☐	MRI-based neuroimaging

Antibodies

Antibodies used	Rabbit monoclonal anti-pan-ADP-ribose binding reagent (Millipore, cat# MABE1016, lot# 3423280; RRID: AB_2665466)<br>Mouse monoclonal anti-6xHis antibody (Takara, cat# 631212, lot# 1906294A; RRID: AB_2721905)

	Polyclonal goat anti-mouse immunoglobulins/HRP (Dako, cat# P0447, lot# 20083051; RRID: AB_2617137)<br>Polyclonal swine anti-rabbit immunoglobulins/HRP (Dako, cat# P0399, lot# 41236529; RRID: AB_2617141)
Validation	All antibodies were validated by the manufacturer for the application in immunoblotting.

Eukaryotic cell lines

Policy information about cell lines

Cell line source(s)	U2OS cells (ATCC, cat# HTB-96)<br>U2OS ARH3 KO cells (Fontana et al., 2017; PMID 28650317, doi: 10.7554/eLife.28533)
Authentication	Cell lines were established and authenticated in earlier manuscripts and were no re-authenticated for this manuscript.
Mycoplasma contamination	All cells used were mycoplasma negative.
Commonly misidentified lines<br>(See ICLAC register)	U2OS cells are not in the ICLAC register of commonly misidentified cell lines (as of release v10).
