# Supplementary material for: Mechanistic insights into the three steps of poly(ADP-ribosylation) reversal
Source: Nat Commun. 2021 Jul 28;12:4581. doi: 10.1038/s41467-021-24723-3 (PMC8319183; doi:10.1038/s41467-021-24723-3)
Supplement: Supplementary file 4 — Source Data [file 41467_2021_24723_MOESM4_ESM.pdf]

Supplementary Information

Mechanistic insights into the three steps of poly(ADP-ribosylation) reversal

Johannes Gregor Matthias Rack[1,&], Qiang Liu[2,&], Valentina Zorzini[1], Jim Voorneveld[2], Antonio Ariza[1], Kourosh Honarmand Ebrahimi[3], Julia M. Reber[4], Sarah C. Krassnig[4], Dragana Ahel[1], Gijsbert A. van der Marel[2], Aswin Mangerich[4], James S. O. McCullagh[3], Dmitri V. Filippov[2,*], Ivan Ahel[1,*]

[1]Sir William Dunn School of Pathology, University of Oxford, South Parks Road, OX1 3RE, Oxford, UK.

[2]Leiden University, Leiden Institute of Chemistry, Einsteinweg 55, 2333 CC, Leiden, The Netherlands.

[3]Department of Chemistry, University of Oxford, Chemistry Research Laboratory, Mansfield Road, OX1 3TA, Oxford, UK.

[4]Molecular Toxicology Group, Department of Biology, University of Konstanz, 78457 Konstanz, Germany

[&]contributed equally

*To whom correspondence should be addressed.

Ivan Ahel:	Tel:(+44) 1865 285 656;	Email: ivan.ahel@path.ox.ac.uk
Dmitri V. Filippov:	Tel:(+31) 71 527 3571;	Email: filippov@chem.leidenuniv.nl

This PDF file includes:

- Supplementary Note 1
- Supplementary Tables 1-7
- Supplementary Figures 1-14
- Supplementary Methods
  - General procedure
  - Part 1. Synthesis of resin **2**.
  - Part 2. Alternative route for the synthesis of parotriose **14**.
  - Part 3. Procedure for the synthesis of each compound
  - Part 4. LC-MS analysis and HRMS analysis of compound **1** and **23**
- Supplementary Figures: NMR spectra
- Supplementary References

Supplementary Note 1.

To systematically describe PAR molecules we propose the following nomenclature:

$$X(ADPr_{ay:n}[ADPr_{by:n}]ADPr_{ay+1:n})$$

with X(…) the protein attachment residue and […] indicators of an inserted branch. Segments belonging to a single linear polymer are indicated by subscript letters followed by segment number (*y*) and the length of the linear segment (*n*) given after the colon.

Example 1: Ser($ADPr_{a1:23}$[$ADPr_{b1:5}$]$ADPr_{a2:10}$)
A linear polymer of length 33 linked to a serine residue with a branch of length 5 attached at position 23

Example 2: Ser($ADPr_{a1:23}$[$ADPr_{b1:5}$[$ADPr_{c1:6}$]$ADPr_{b2:10}$]$ADPr_{a2:7}$)
A serine-linked linear polymer of length 30 with a branch point at position 23. The branch is of length 15 with a further branch insertion of length 6 at position 5 of the branch.

Following this nomenclature we denote the ADPr moieties in our branch point molecule $ADPr_{a1:n}$, [$ADPr_{b1:1}$] and $ADPr_{a2:1}$ (Supplementary Table 1).

Supplementary Table 1. Chemical structures of selected ADP-ribose derivatives

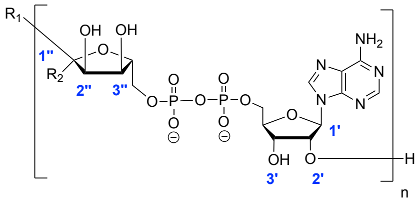

Compound names	R_1	R_2	n
**ADP- ribose**	OH or H	H or OH	1
**meADPr**	OCH_3	H	1
**1''-*O*-acetyl-ADPr**	*O*Ac	H	1
**α-NAD⁺**	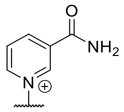	H	1
**Ser-ADPr**	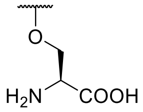	H	1
**Glu-ADPr**	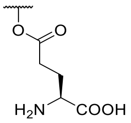	H	1
**Asp-ADPr**	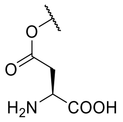	H	1
**dimer (di-ADPr)**	OCH_3	H	2
**trimer (tri-ADPr; linear)**	OCH_3		3
**PAR (linear)**	OH or H	H or OH	variable
**PAR branch point**	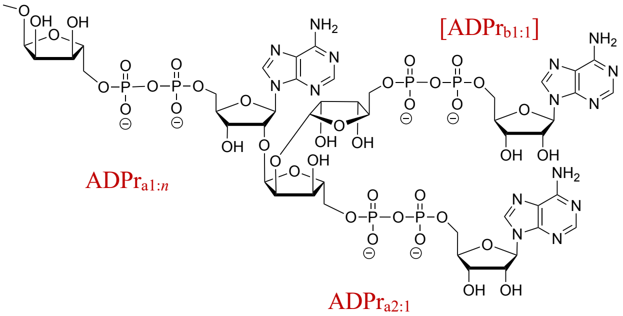		

Supplementary Table 2. Data collection and refinement statistics.

	*h*ARH3 E41A:H2BS7mar (PDB 7AKS)	*h*ARH3 E41A:dimer (PDB 7AKR)	*h*ARH3 E41A:α-NAD$^+$ (PDB 7ARW)	*Lch*ARH3:meADPr (PDB 7AQM)
**Data collection**				
Space group	$P2_1\ 2_1\ 2$	$P2_1$	$P1$	$P2_1\ 2_1\ 2_1$
Cell dimensions				
a, *b*, *c* (Å)	123.36, 158.63, 74.92	86.62, 91.60, 91.09	44.74, 66.11, 70.05	66.82, 97.61, 107.39
α, β, γ (°)	90.00, 90.00, 90.00	90.00, 105.47, 90.00	115.80, 94.97, 104.07	90.00, 90.00, 90.00
Resolution (Å)	158.62-1.86 (1.91-1.86)*	70.63-1.95 (2.02-1.95)*	61.52-1.31 (1.35-1.31)*	56.72-2.50 (2.58-2.50)*
R_{sym} or R_{merge}	16.7(182.4)	14.5(153.5)	7.07(8.97)	5.49(9.13)
$I / \sigma I$	12.9(1.4)	7.9(1.3)	9.79(0.70)	7.37(0.88)
Completeness (%)	99.9(97.7)	99.87(99.97)	95.62(92.95)	98.97(98.00)
Redundancy	21.4(11.5)	6.6(6.7)	6.6(6.4)	2.0(2.0)
**Refinement**				
Resolution (Å)	97.38-1.86	70.63 -1.95	34.78-1.31	53.70-2.50
No. reflections	124916(11488)	99824(9977)	157282(15352)	24695(2396)
R_{work} / R_{free}	0.176/0.197	0.188/0.208	0.166/0.182	0.246/0.284
No. atoms				
Protein	10552	10441	5150	4903
Ligand/ion	341	327	148	78
Water	785	438	759	53
B-factors				
Protein	30.77	46.31	25.20	74.52
Ligand/ion	35.82	47.22	28.71	82.09
Water	38.42	45.87	40.99	61.16
R.m.s. deviations				
Bond lengths (Å)	0.016	0.015	0.006	0.010
Bond angles (°)	1.83	1.69	1.13	1.54

*Number of xtals: 1.

Supplementary Table 3. COSMIC variants summary.

mutation (aa)	mutation (CDS)	count	tissue	legacy mutation ID
D34G	c.110A>G	1	skin carcinoma	COSM5033871
T76R	c.227C>G	4	prostate (adenocarcinoma)	COSM5992851
S185P	c.553T>C	1	hairy cell leukaemia, variant	COSM3727906
L186V	c.556C>G	2	squamous cell carcinoma; pancreas*	COSM83890
G270C	c.808G>T	1	liver (neoplasm)	COSM6262998

*not specified

Supplementary Table 4. ARH3 sequences utilised to generate multiple sequence alignment.

common name	binominal name	accession[*]
human	*Homo sapiens*	NP_060295.1
cat	*Felis catus*	XP_023114243.1
mouse	*Mus musculus*	NP_598644.1
ocelot gecko	*Paroedura picta*	GCF42864.1
great tit	*Parus major*	XP_015504659.1
gombessa	*Latimeria chalumnae*	XP_005988572.1
whale shark	*Rhincodon typus*	XP_020390629.1
Gaboon caecilian	*Geotrypetes seraphini*	XP_033812889.1
tropical clawed frog	*Xenopus tropicalis*	NP_001016184.1

[*]GenBank accession numbers

Supplementary Table 5. Overview of distances (Å) and angles (°) between nicotinamide and Mg_{II} ion in the α-NAD^+ crystal structure.

		chain A	chain B
distances	Mg···N1	3.51	3.32
	Mg···C2	3.37	3.10
	Mg···C3	3.59	3.36
	Mg···C4	4.00	3.88
	Mg···C5	4.09	4.02
	Mg···C6	3.87	3.78
	Mg···Nam (centroid)	3.47	3.29
	Mg···Nam (plane)	2.79	2.09
angles	$\theta_{N1\text{-}C2}$	75.33	71.86
	$\theta_{C2\text{-}C3}$	77.28	72.79
	$\theta_{C3\text{-}C4}$	92.16	91.13
	$\theta_{C4\text{-}C5}$	104.71	108.20
	$\theta_{C5\text{-}C6}$	102.38	106.85
	$\theta_{C6\text{-}N1}$	87.86	88.81
LISP*		2.07†	2.54†

*Label-independent slippage parameter (LISP) defined as

$$LISP\ [\text{Å}] = \frac{l_c}{N}\sum_{i=1}^{N}\left|sin\left(\theta_i - \frac{\pi}{2}\right)\right|$$

with l_c, Mg···Nam (centroid) distance; N, number of atoms in the ring; θ_i, the angle between the middle point of two carbon atoms, the ring centroid, and the magnesium

†Projection of Mg on the ring plane is outside the aromatic ring.

Supplementary Table 6. Oligonucleotides used in this study.

name	purpose*	sequence
A3E41A_Xtal_for	s.d.m.	GAAGTTCTGTTTCAGGGTCCGGGCAGCTCCCTCTCGCGC TTCCGAGGCTGCC
A3E41A_Xtal_rev	s.d.m.	GCTGCCCGGACCCTGAAACAGAACTTCCAAACTTGTGAT ATCGTGATGGTGGTGGTGG
hARH3_D34G_for	s.d.m.	GCTCGGGGctTGCGTGGGCTCCTTCTACG
hARH3_D34G_rev	s.d.m.	GAGCCCACGCAagCCCCGAGCAGCGCGCCAGCC
hARH3_E41A_for	s.d.m.	CCTTCTACGcGGCCCACGACACCGTCGAC
hARH3_E41A_rev	s.d.m.	CGTGGGCCgCGTAGAAGGAGCCCACGC
hARH3_T76R_for	s.d.m.	GTACTACAgAGATGACACAGCCATGGC
hARH3_T76R_rev	s.d.m.	GTGTCATCTcTGTAGTACAAGGCTTCTG
hARH3_D77N_for	s.d.m.	GTACTACACAaATGACACAGCCATGGCCAG
hARH3_D77N_rev	s.d.m.	CATGGCTGTGTCATtTGTGTAGTACAAGGCTTCTGTC
ARH3 D78N F	s.d.m.	CTTGTACTACACAgATAACACAGCCATGGC
ARH3 D78N R	s.d.m.	GCCATGGCTGTGTTATcTGTGTAGTACAAG
ARH3_G115S_FOR	s.d.m.	CCTGACAGGaGCTATGGTGCTGGAGTAGTC
ARH3_G115S_REV	s.d.m.	CAGCACCATAGCtCCTGTCAGGGTCTTTC
hARH3_F143L_for	s.d.m.	GGGCCCAGcTTAACGGGAAAGGCTCCTATGGC
hARH3_F143L_rev	s.d.m.	CCTTTCCCGTTAAgCTGGGCCCGGGCAGGCTC
hARH3_S148A_for	s.d.m.	CGGGAAAGGCgCCTATGGCAATGGAGGTGC
hARH3_S148A_rev	s.d.m.	CCATTGCCATAGGcGCCTTTCCCGTTAAACTGGGCCC
hARH3_Y149L_for	s.d.m.	GAAAGGCTCCctTGGCAATGGAGGTGCCATGCGG
hARH3_Y149L_rev	s.d.m.	CCTCCATTGCCAagGGAGCCTTTCCCGTTAAACTGGG
hARH3_G150E_for	s.d.m.	GGCTCCTATGagAATGGAGGTGCCATGCGG
hARH3_G150E_rev	s.d.m.	GCACCTCCATTctCATAGGAGCCTTTCCCGTTAAAC
hARH3_S185P_for	s.d.m.	CACGCCTCCcCCCTGGGTTACAATGGCGCCATCC
hARH3_S185P_rev	s.d.m.	GTAACCCAGGGgGGAGGCGTGTGTCAGCTGGG
hARH3_L186V_for	s.d.m.	CCTCCTCCgTGGGTTACAATGGCGCC
hARH3_L186V_rev	s.d.m.	GTAACCCAcGGAGGAGGCGTGTGTCAGC
hARH3_N269A_for	s.d.m.	GAGCTAGGGgcTGGCATTGCTGCCTTTGAGTCGG
hARH3_N269A_rev	s.d.m.	GCAGCAATGCCAgcCCCTAGCTCAGACACCACTTCC
hARH3_G270C_for	s.d.m.	CTAGGGAATtGCATTGCTGCCTTTGAGTCGG
hARH3_G270C_rev	s.d.m.	GCAGCAATGCaATTCCCTAGCTCAGACACC
hARH3_I271A_for	s.d.m.	CTAGGGAATGGCgcTGCTGCCTTTGAGTCGGTACCC
hARH3_I271A_rev	s.d.m.	CTCAAAGGCAGCAgcGCCATTCCCTAGCTCAGACACC
PARP1_G972R_for	s.d.m.	cgttcctcttAggaccgggatttcatctgg
PARP1_G972R_rev	s.d.m.	cccggtccTaagaggaacgtctacacc
PARP1_27f (Y986H)	s.d.m.	gtgaatgacacctctctactacataacgagtacattgtctatg
PARP1_27r (Y986H)	s.d.m.	catagacaatgtactcgttatgtagtagagaggtgtcattcac
PARP1_28f (Y986S)	s.d.m.	atatcatagacaatgtactcgttacttagtagagaggtgtcattcacacc
PARP1_28r (Y986S)	s.d.m.	ggtgtgaatgacacctctctactaagtaacgagtacattgtctatgatat

*s.d.m., site-directed mutagenesis

Supplementary Table 7. Source and analyzer settings of the Xevo TQ-S mass spectrometer for the detection of R-Ado and R_2-Ado.

**Source settings**	**Value**
Capillary (kV)	1.0
Cone (V)	7.0
Source offset (V)	50.0
Source temperature (°C)	150
Desolvation temperature (°C)	500
Cone gas flow (l/h)	150
Desolvation gas flow (l/h)	1000
Collision gas flow (ml/min)	0.15
Nebulizer gas flow (bar)	7.0
**Analyzer settings**	**Value**
LM1 resolution	3.0
HM1 resolution	14.9
Ion energy 1	0.1
MS mode collision energy	4.0
MS-MS mode collision energy	20.0
MS mode entrance	1.0
MS mode exit	1.0
LM2 resolution	3.0
HM2 resolution	15.0
Ion energy 2	0.6
Gain	1.0
Multiplier	0.0

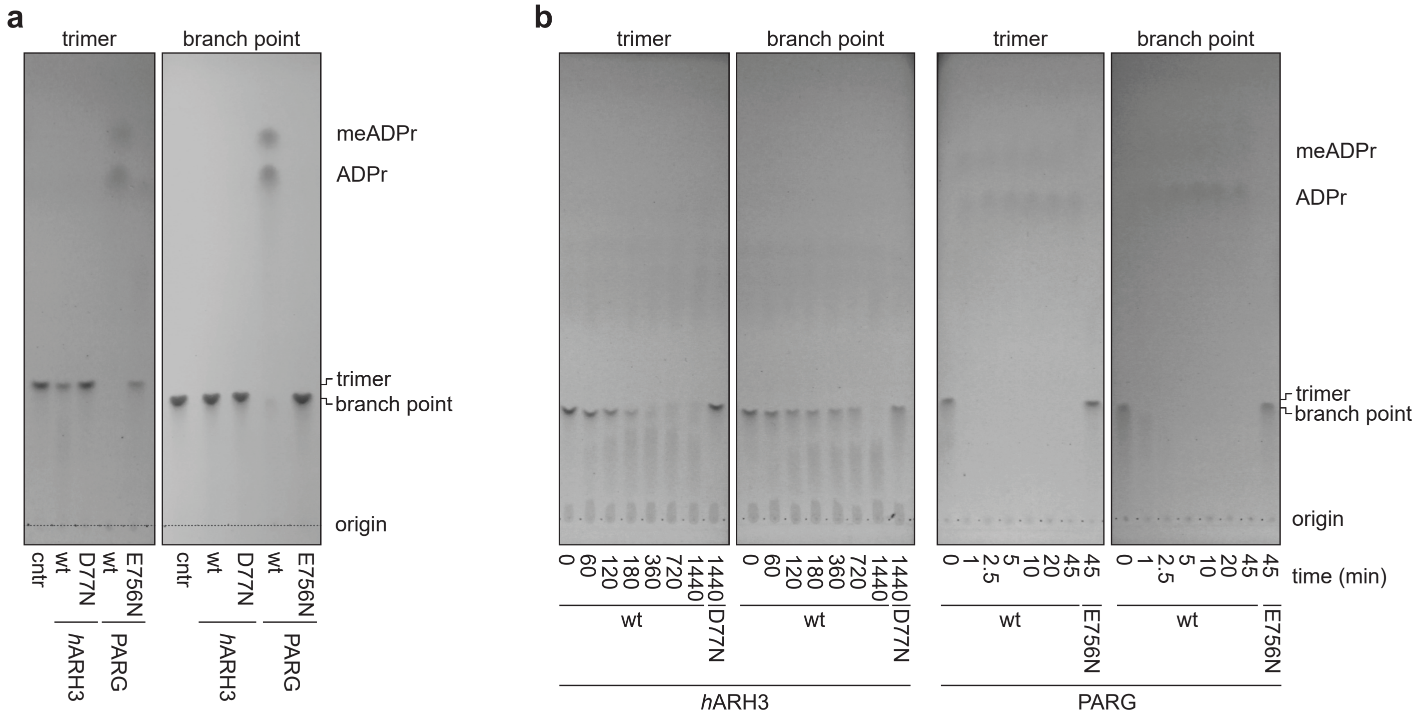

Supplementary Fig. 1. PAR degradation by *h*ARH3 and PARG. (**a**) Synthetic PAR trimer and branch point degradation by *h*ARH3 and PARG. Samples were analysed by thin-layer chromatography (TLC) and representative results of three independent experiments are shown. (**b**) Time-course of synthetic PAR trimer and branch point degradation by *h*ARH3 and PARG. Samples were analysed by TLC and representative results of two independent experiments are shown. Source data are provided as a Source Data file.

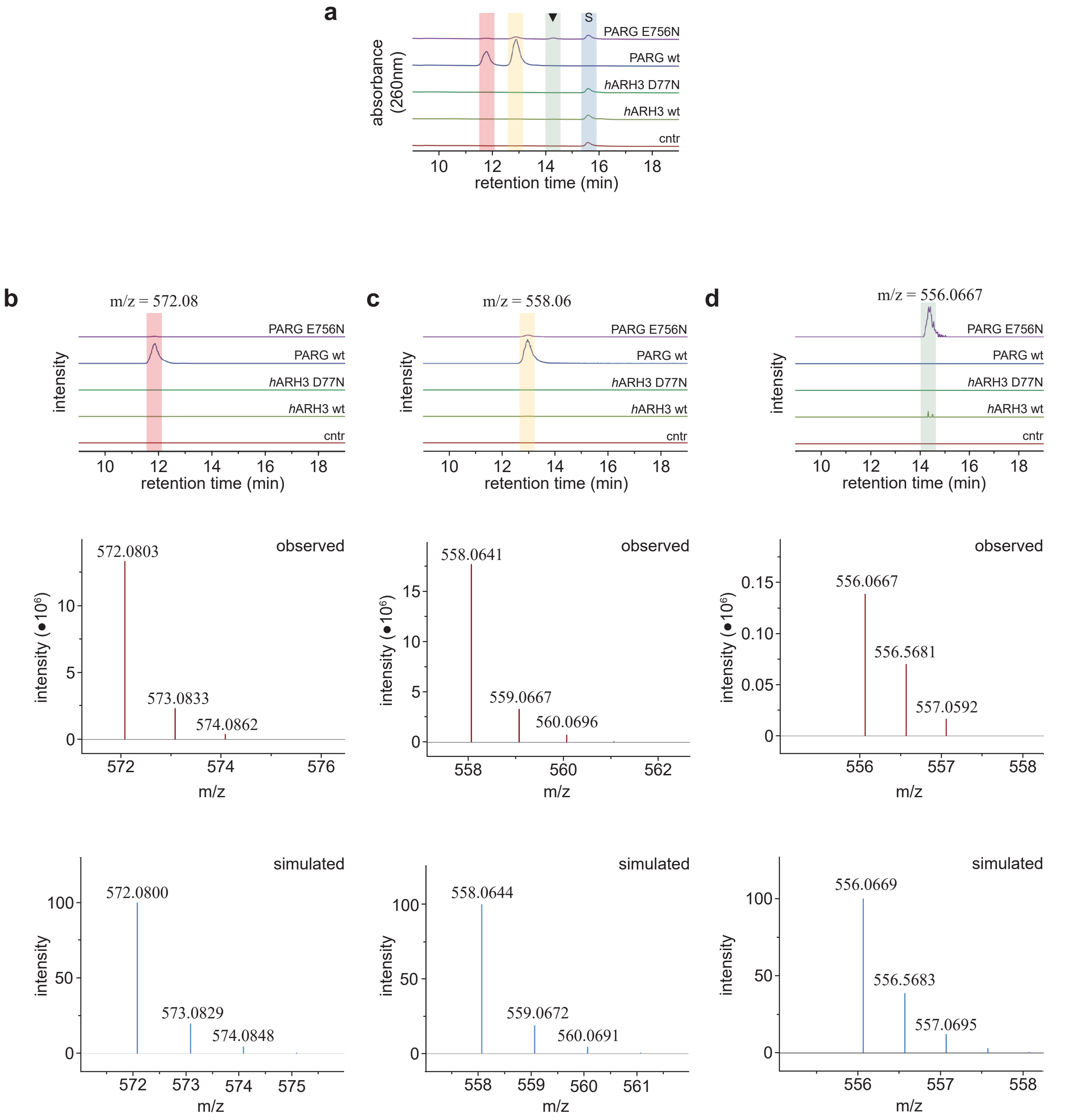

Supplementary Figure 2. High-resolution mass spectrometry analysis of hydrolysis of PAR branch point. (**a**) UV visible chromatograms with S denoting PAR branch point and the black triangle the PARG E756N specific peak. (**b+c**) Total negative ion count for ADPr $[M-H]^-$ with a m/z of 572.08 (**b**) and 558.06 (**c**) (upper panels) and high-resolution mass spectrum of meADPr (**b**) and ADPr (**c**) (lower panels). (**d**) Total negative ion count for $[M-2H]^{2-}$ of a molecules with m/z of 556.0667 (upper panel). Ion chromatograms were extracted setting tolerance at 10 ppm. These molecules are best identified with chemical formula of $C_{16}H_{25}N_5O_{14}P_2$ (**b**), $C_{15}H_{23}N_5O_{14}P_2$ (**c**) and $C_{31}H_{46}N_{10}O_{27}P_4$ (**d**), which corresponds to meADPr (**b**), ADPr (**c**) and a meADPr-ADPr dimer (**d**). Source data are provided as a Source Data file.

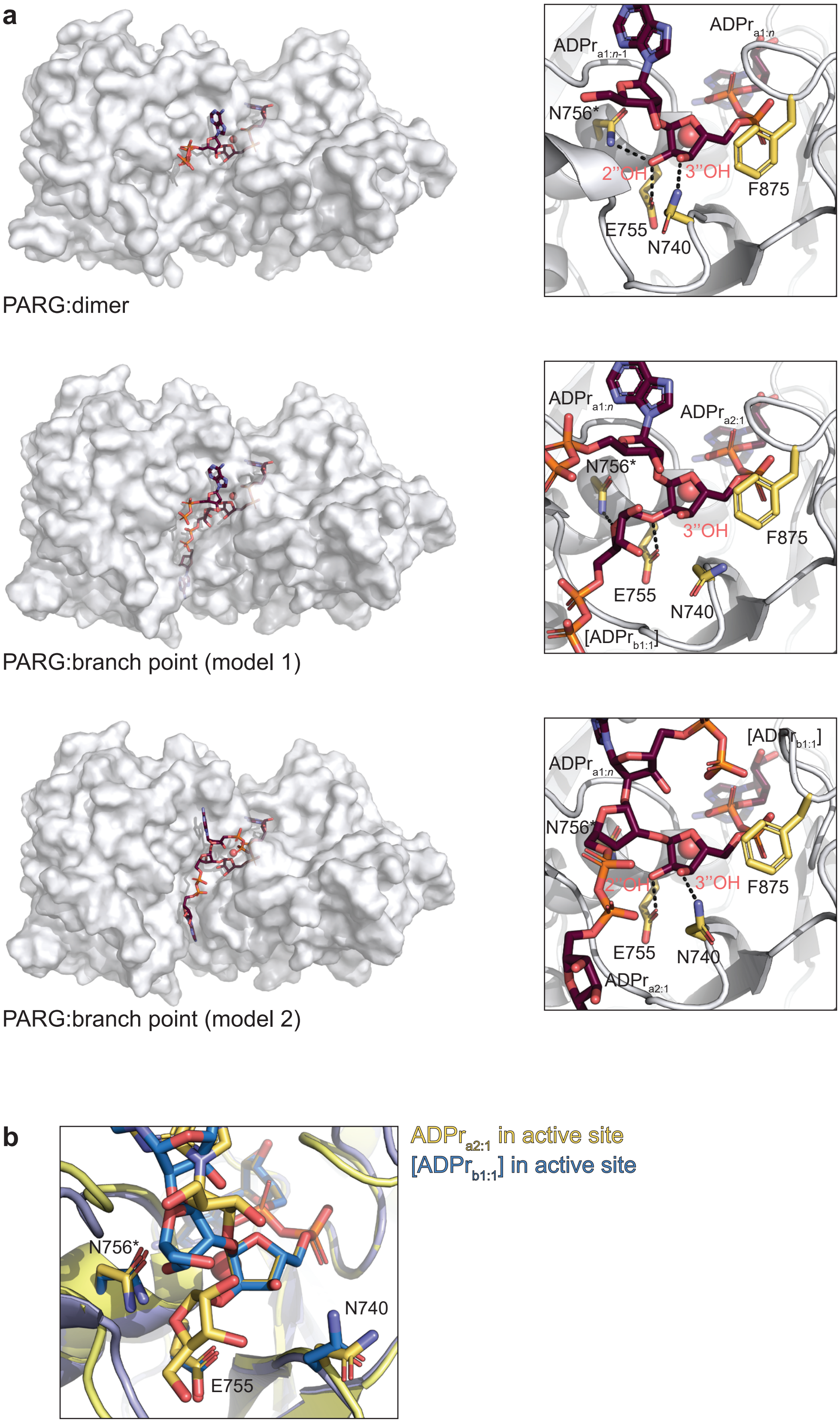

Supplementary Figure 3. Modelling of the PARG:branch point interaction. Energy minimised models of PARG in complex with branch point PAR. Allowing free movement of the whole ligand during energy minimisation resulted in models which did not maintain catalytic contacts (top panel (a)). To analyse whether adoption of a catalytic conformation is theoretically possible, we fixed the position of the ADPr moiety bound in the active site. All modeled complexes had comparable energy values with fixed ligand model energies <4% higher than the free ligand models. (**a**) Surface representations of PARG in complex with PAR dimer (PDB A5R7; top panel) and branch point (model 1 [middle panel]: $ADPr_{a2:1}$ (linear PAR continuation) or model 2 [bottom panel]: [$ADPr_{b1:1}$] (branched ADPr) placed in the active site). Close ups (right panels) depict the coordination of ligand by the catalytic residues. Models are derived from energy minimisation with fixed orientation for the ADPr moiety bound in the active site. Asterisk, the structure and models are of the catalytic mutant E756N. (**b**) Close-up comparison of the active site coordination of the two branched PAR models. Note, some foreground ligand atoms were removed for clarity.

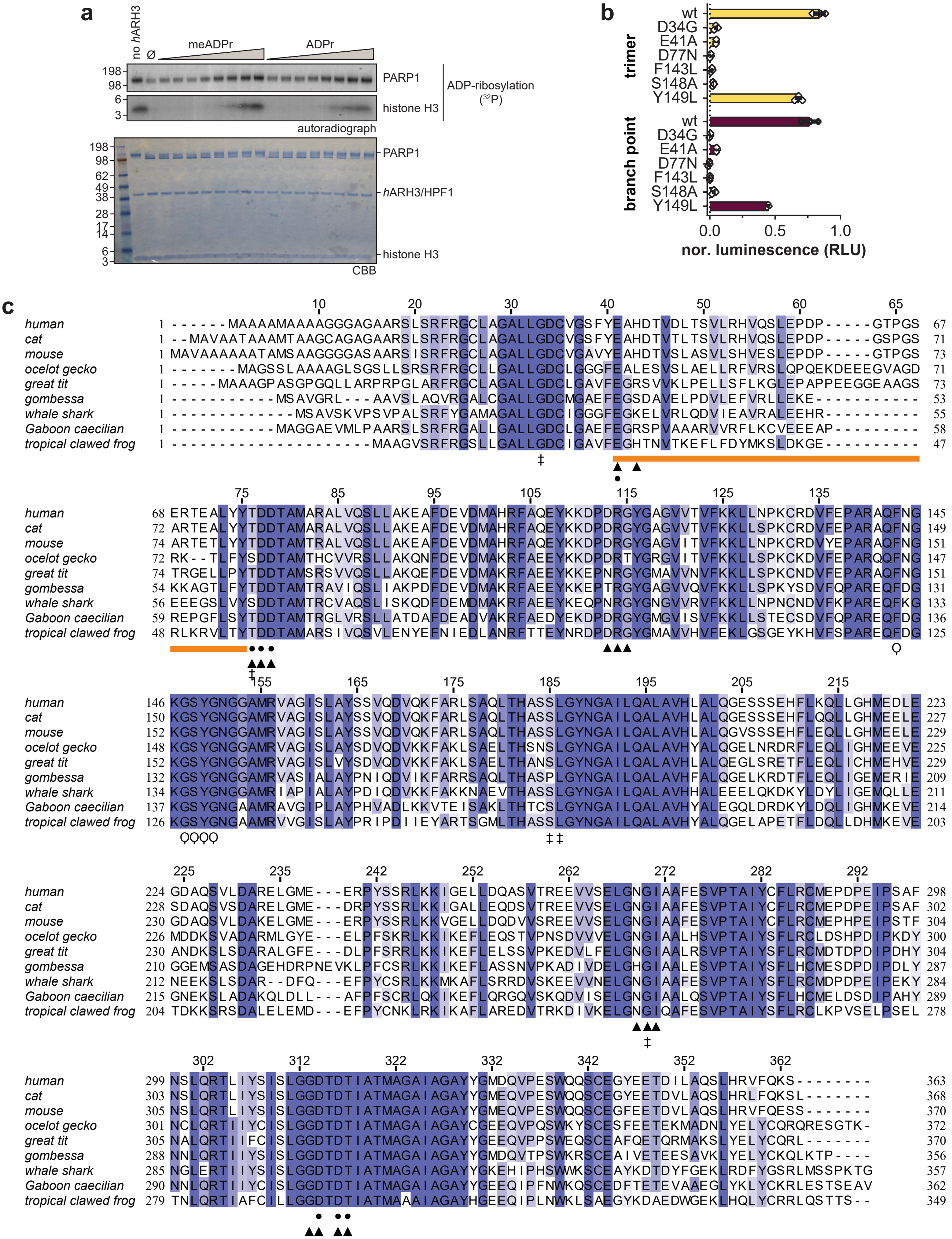

Supplementary Figure 4. Manipulation of ARH3 activity. (**a**) Analysis of *h*ARH3 inhibition by ADPr and meADPr. The reactions were supplemented with increasing amounts of compound (1, 5, 10, 50, 100, 250, 500, and 1,000 µM) as indicated. (**b**) Analysis of PAR trimer and branch point hydrolysis by different *h*ARH3 mutants via AMP detection (methods). All data were normalised to full conversion by PARG and represented as mean values (open diamonds) ± s.e.m. from three independent experiments measured in triplicates. (**c**) Sequence conservation amongst ARH3 homologues. The multiple sequence alignment was generated using MAFFT L-INS-i as integrated into Jalview v2.11 using sequences listed in Supplementary Tab. 4. Numbering corresponse to the position in the *h*ARH3 sequence and residues of interest are highlighted underneath the alignemnt: double daggers, mutations found in the COSMIC database and used in the work (Supplementary Tab. 3); koppa, residues involved in adensosine binding; black circle, magnesium coordinating residues; black triangle, residues involved in peptide binding and Glu41-flap region (orange line). Source data are provided as a Source Data file.

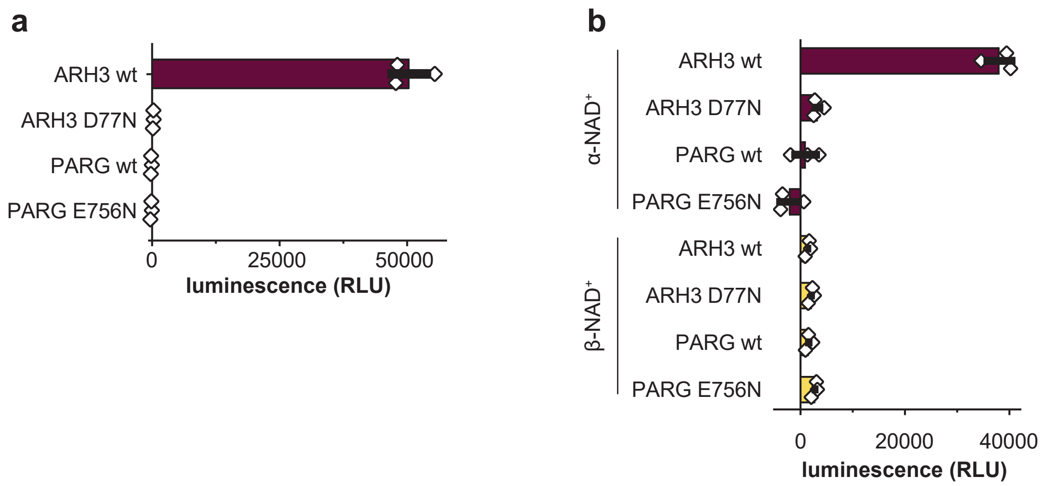

Supplementary Figure 5. Controls for the *h*ARH3 activity. (**a**) Analysis of hydrolysis of H2BS7mar peptide by *h*ARH3 and PARG. Data represented as mean values (open diamonds) ± s.e.m. from three independent samples measured in triplicates. (**b**) Analysis of stereoselectivity of NAD^+ hydrolysis by *h*ARH3 and PARG. Data represented as mean values (open diamonds) ± s.e.m. from three independent samples measured in triplicates. Source data are provided as a Source Data file.

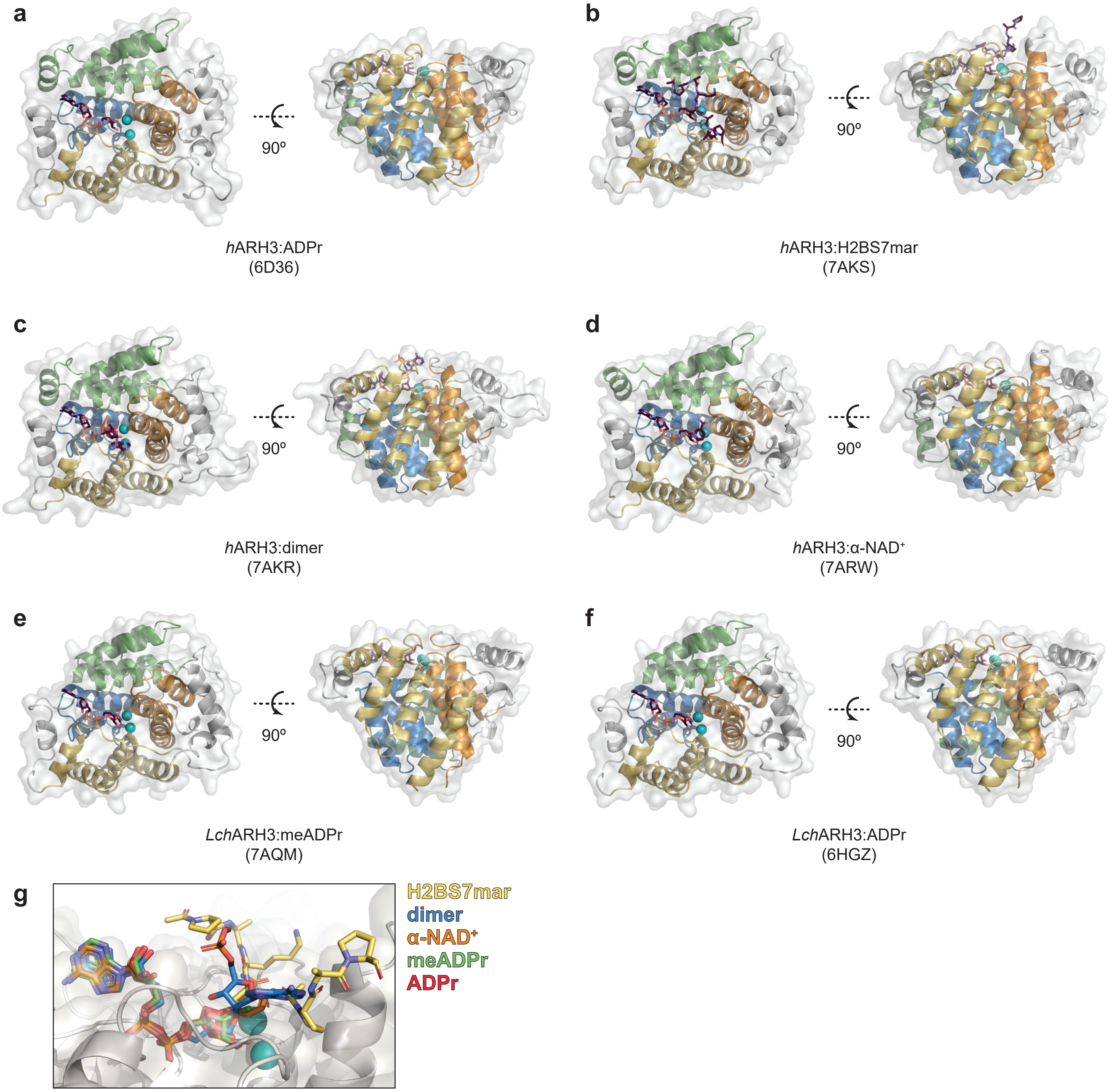

Supplementary Figure 6. Structural overview and comparison of ARH3 ligand complexes. (**a-f**) Ribbon-surface representation of *h*ARH3 (a-d) and *Lch*ARH3 (e+f) structures in complex with different ligands (purple). The four quasidomains composing the core fold are coloured in orange (A), yellow (B), blue (C), and green (D). The magnesium ions in the catalytic centre are given in turquoise. (**g**) Comparison of ligand placement within the ligand binding site of ARH3. For clarity only the ribbon-surface of the *h*ARH3:H2BS7mar structure is given. The comparison is derived from the structures 7AKS, 7AKR, 7ARW, 7AQM, and 6HGZ using PyMol v2.4.

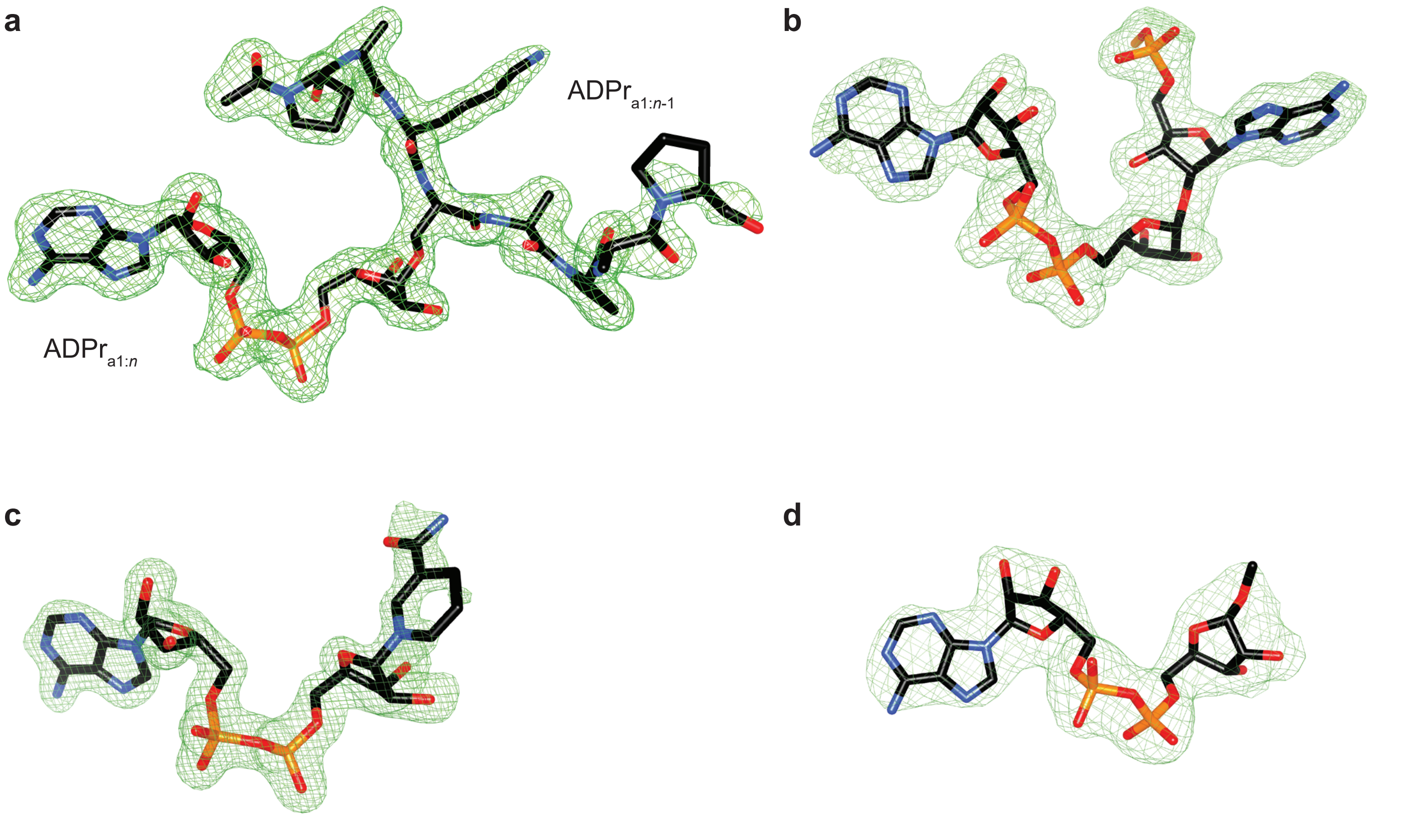

Supplementary Figure 7. Electron density evidence for the structure bound ligands. (**a-d**) Composite omit maps (green) of the different ligands contoured at 2σ. (**a**) H2BS7mar (**b**) dimeric ADPr (**c**) α-NAD^+ and (**d**) meADPr.

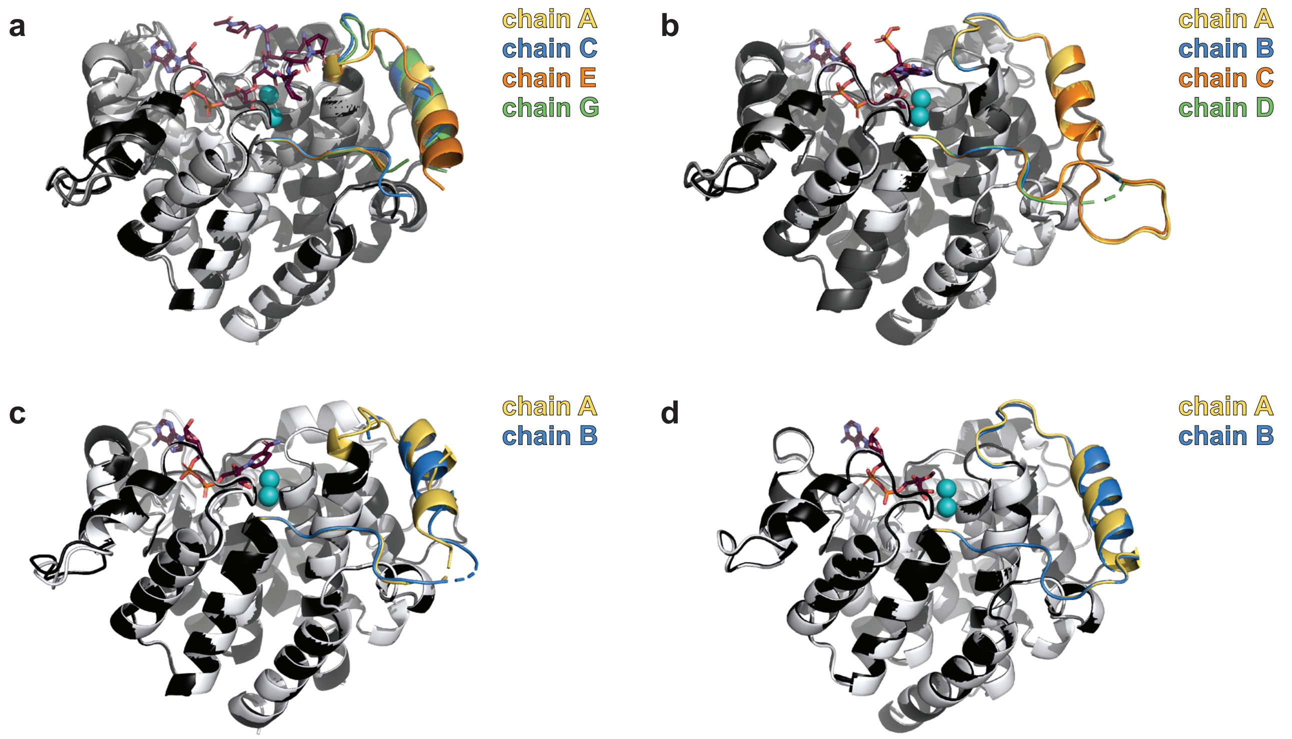

Supplementary Figure 8. Comparison of Glu41-flap conformations within the different chains in the asymmetric unit. (**a**) *h*ARH3:H2BS7mar, (**b**) *h*ARH3:dimer, (**c**) *h*ARH3:α-NAD+, and (**d**) *Lch*ARH3:meADPr. The Glu41-flap region is coloured as indicated and the remaining structure in ascending greyscale starting with white for chain A. For clarity only ligands corresponding to chain A are shown.

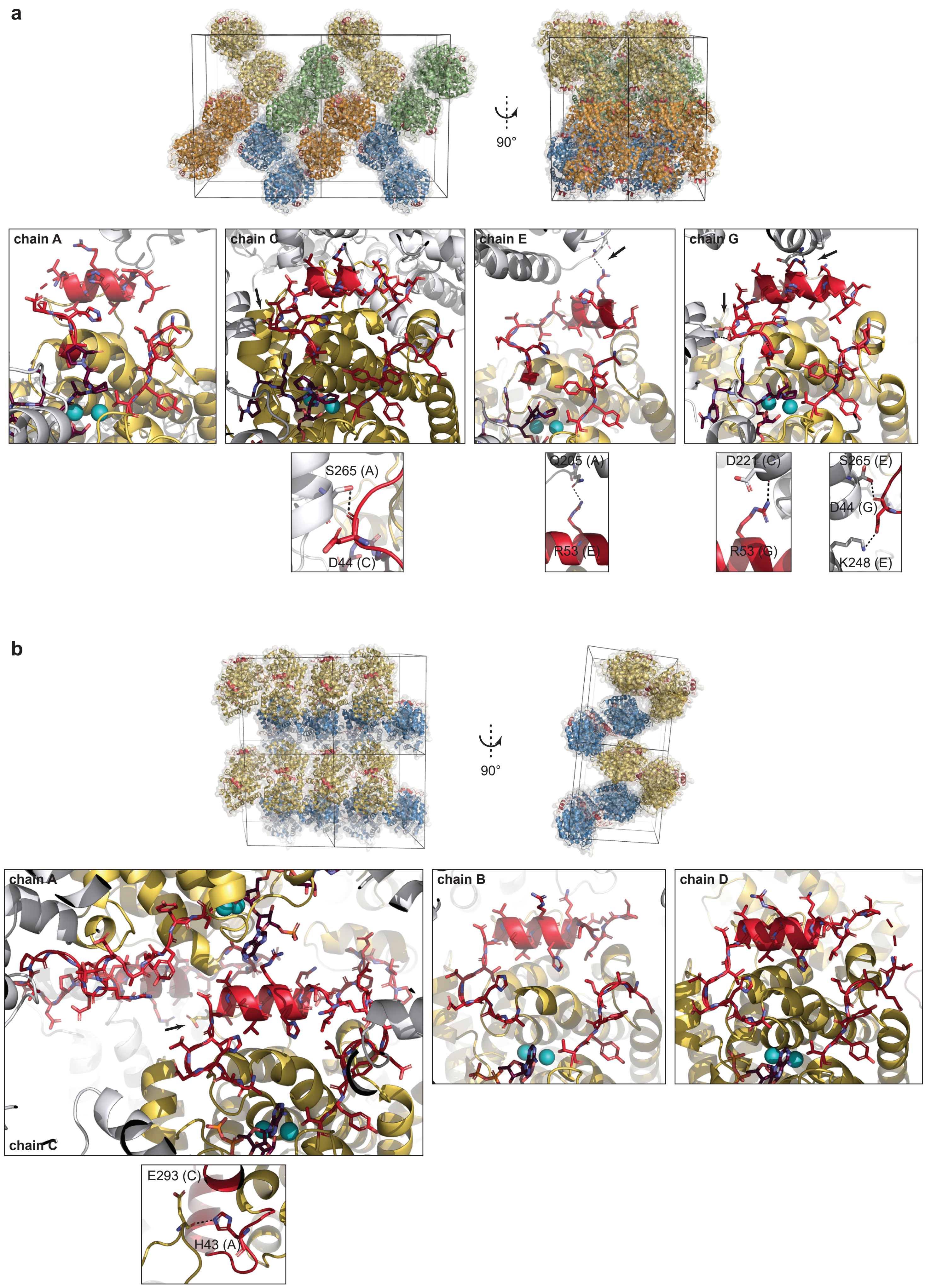

Supplementary Figure 9. (page 1 of 2)

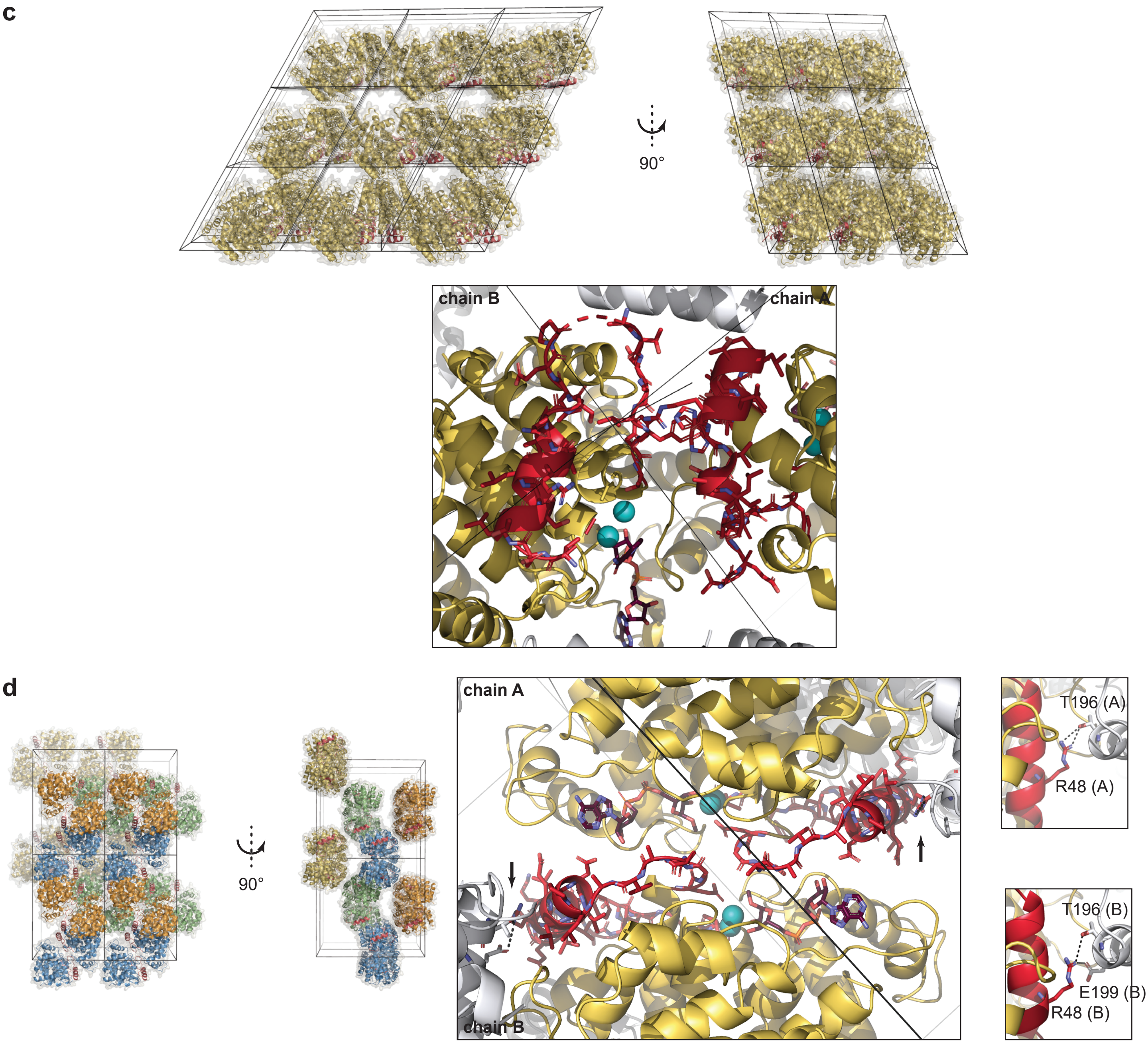

Supplementary Figure 9. (page 2 of 2) Analysis of crystal packing differences between the *h*ARH3 crystal forms. Top panels show crystal lattices, middle panel show packing of Glu41-flap (red) for the different chain within the respective crystal and bottom panels show polar contacts of Glu41-flap residues with other residues within the crystal. Positions of these contacts are indicated by arrows in the middle panels and their chain identity is given in parenthesis. Note, chain belonging to the same asymetric unit (AU) are given in yellow in the middle and bottom panel and symetry related molecules are given in white. (**a**) *h*ARH3:H2BS7mar crystallised in $P2_12_12$ with four molecules in the AU and four AUs in the unit cell (UC). AUs within the UC are coloured yellow, blue, orange and green. (**b**) *h*ARH3:dimer crystallised in $P2_1$ with four molecules in the AU and two AUs (yellow/blue) in the UC. (**c**) *h*ARH3:α-NAD$^+$ cystallised in $P1$ with two molecules in the AU and one AU per UC. Note, no contacts within the Glu41-flap could be identified and the bottom panels was omitted. (**d**) *Lch*ARH3:meADPr-crystallised in $P2_12_12_1$ with two molecules in the AU and four AUs in the UC.

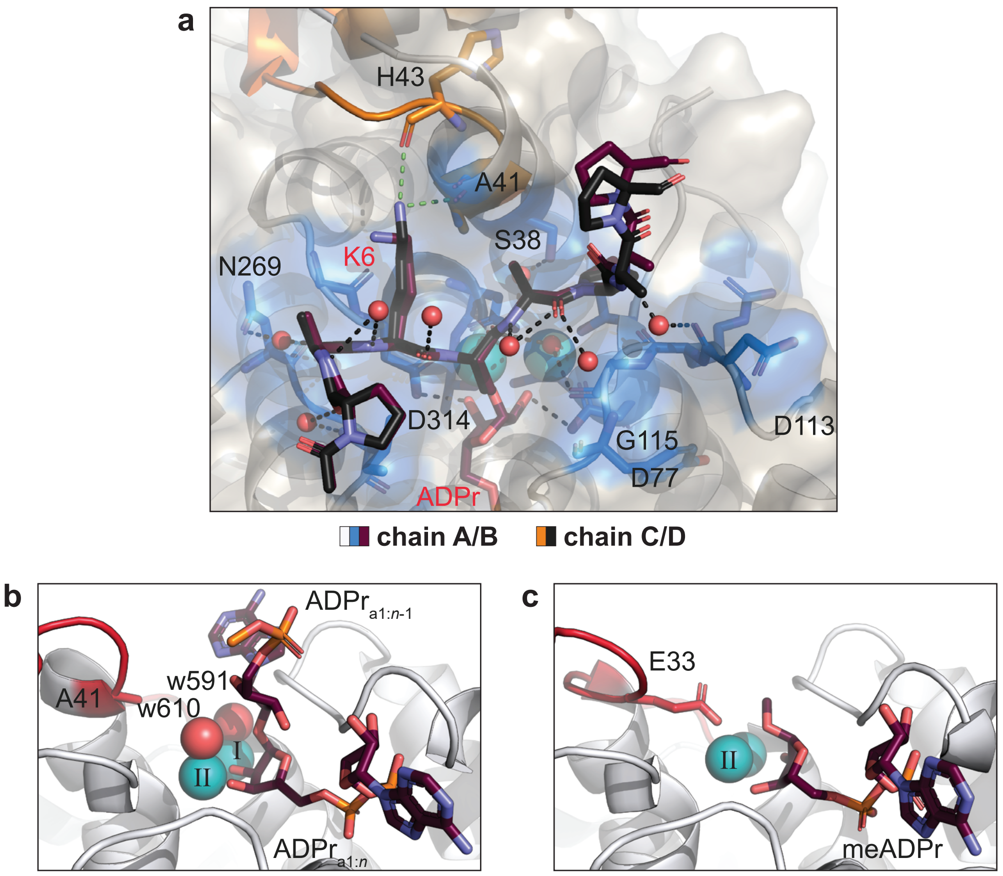

*h*ARH3:dimer
(7AKR)

*Lch*ARH3:meADPr
(7AQM)

Supplementary Figure 10. ARH3 ligand coordination. (**a**) Close up of the peptide binding surface (blue) of *h*ARH3. Interactions between chain A (ARH3) and B (H2BS7mar) are given in black, additional contacts in chains C/D in green, selected residues of *h*ARH3 are labeled in black and of the peptide in red. (**b+c**) Ribbon representation of the ARH3 active site with ligand highlighted in purple, magnesium ions in turquoise and the Glu41-flap in red. (**b**) Close up of PAR dimer coordination. Note, in the wt structure displacement of w610 by Glu41 appears possible. (**c**) Close up of meADPr coordination by *Lch*ARH3. Note, due to resolution the predicted axial water of Mg_I is not resolved in the structure.

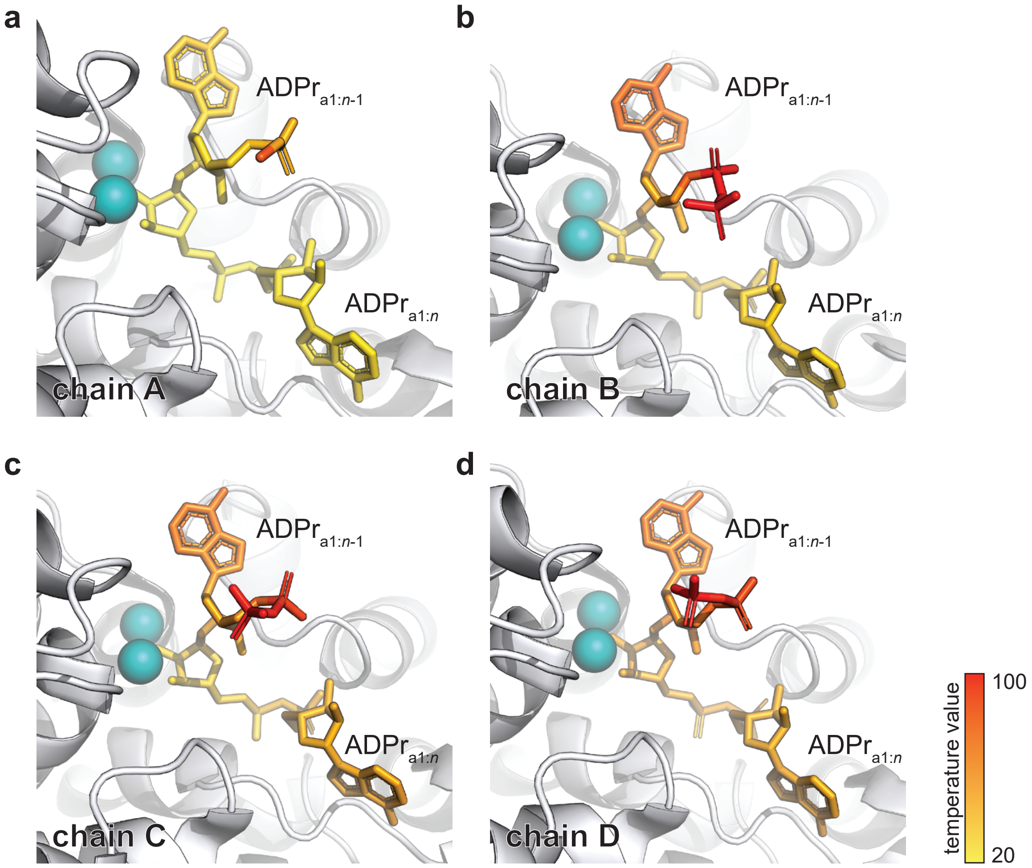

Supplementary Figure 11. Analysis of temperature values (B-factors) of the dimeric ADPr ligands (PDB 7AKR). (**a-d**) Represented are all four chains found in the asymetric unit. The atom B-factors are given as heat map scaled as indicated and the active site magnesium ions (turquoise) are given for orientation.

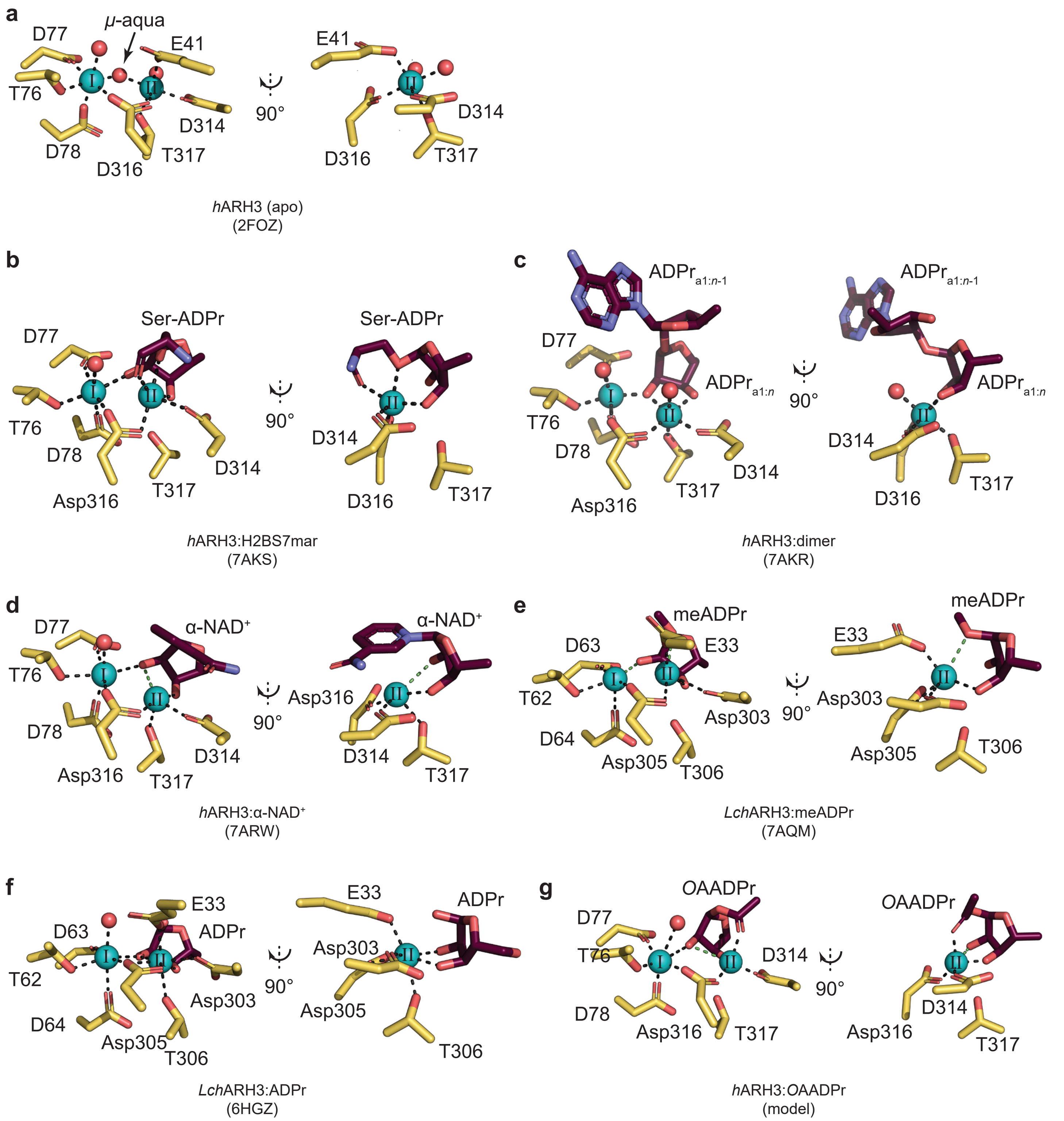

Supplementary Figure 12. Influence of ligand binding on the coordination of the catalytic magnesium ions. (**a-g**) Liquorice representation of distal ribose orientation and metal coordination within the active site. Ligands are given in purple, protein in yellow, magnesium ions in turquoise, water molecules in red, ligand metal contacts as black lines and potential long distant contacts (2.8-3.2 Å) as green lines. (**a**) apo form, (**b**) H2BS7mar, (**c**) dimeric ADPr, (**d**) α-NAD$^+$, (**e**) meADPr, (**f**) ADPr, and (**g**) *O*AADPr.

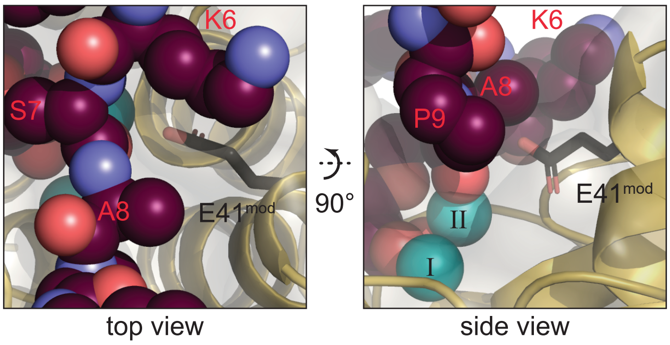

Supplementary Figure 13. Peptide binding limits the ability of Glu41 to adopt different rotamers. Ribbon-surface representation of *h*ARH3 A41E (reversal to wt) model in complex with the H2BS7mar peptide (purple spheres, residue labels red). The bound peptide imposes sterical constrains on the position of the modeled Glu41 residue (E41^{mod}), thus supporting repositioning into the second coordination sphere.

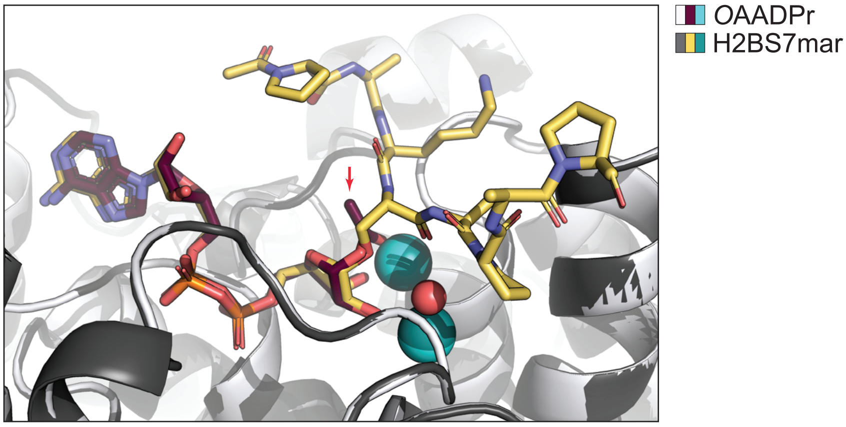

Supplementary Figure 14. Comparison of *O*AADPr and H2BS7mar binding to *h*ARH3. The model of *O*AADPr binding follows closely the binding of the H2BS7mar peptide within the active site. However, positioning of the methyl moiety of the *O*Ac group (arrow) suggests that binding of Asp-/Glu-ADPr peptides may be unfavourable.

Supplementary Methods

General procedure

All solvents used were stored over molecular sieves and all reactions were carried out in oven or flame-dried glassware. Unless stated otherwise, all solvents were removed by rotary evaporation under reduced pressure at 40 ℃. Reactions were monitored by TLC-analysis using silica gel 60 F_{254} (Merk Millipore) with detection by spraying with 20% H_2SO_4 in MeOH or $(NH_4)_6Mo_7O_{24}\cdot 4H_2O$ (25g/L) and $(NH_4)_4Ce(SO_4)_4\cdot 2H_2O$ in 10% sulfuric acid, followed by charring at approx. 150℃. LC-MS analysis was performed on a Thermo Finnigan LCQ Advantage MAX ion-trap mass spectrometer with an electrospray ion source coupled to Surveyor HPLC system (Thermo Finnegan) using an analytical Gemini C18 column (Phenomex, 50 x 4.60 mm, 3 micron) in combination with eluents A: H_2O; B: MeCN and C: 1% aq. TFA as the solvent system. High resolution mass spectra were recorded by direct injection (2 μL of a 2 μM solution in water/acetonitrile; 50/50; v/v and 0.1% formic acid) on a mass spectrometer (Thermo Finnigan LTQ Orbitrap) equipped with an electrospray ion source in positive mode with resolution R = 60000 at m/z 400 (mass range m/z = 150-2000) and dioctylpthalate (m/z = 391.2842) as a "lock mass". The high resolution mass spectrometer was calibrated prior to measurements with a calibration mixture (Thermo Finnigan). ^{1}H-, ^{13}C- and ^{31}P-NMR spectra were measured on Brüker DPX-300, Brüker AV-400/500/600/850 and all individual signal was assigned using 2D-NMR spectroscopy. Chemical shifts were given in ppm (δ) relative to TMS (0 ppm) or indirectly referenced to H_3PO_4 (0.00 ppm) in D_2O via the solvent residual signal and coupling constants were given in Hz. Infrared (IR) spectra were record on a Shimadzu FT-IR 8300. Optical rotation was measured by MCP 100 Modular Circular Polarimeter using methanol as solvent. LCAA-CPG resin were purchased from Sigma-Aldrich. Tentagel N resin was purchased from Rapp Polymere (product name: TentaGel® N NH_2, product number: N30002).

Part 1. Synthesis of resin 2

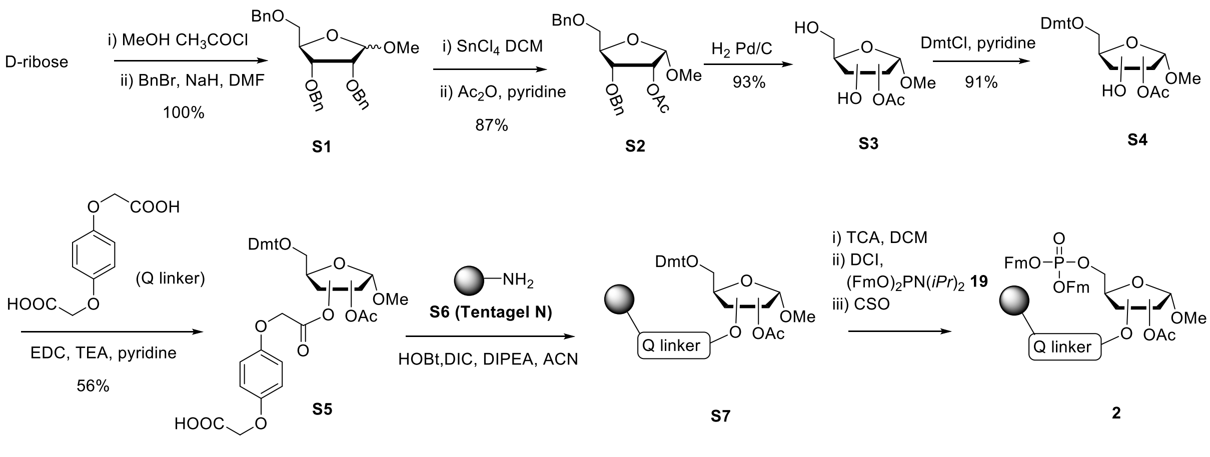

Supplementary Figure 15. Synthesis of the resins **2**.

The solid phase procedure of branched ADPr target **1** oligomer started with the functionalisation of Tentagel N (TG) solid supports. Apart from the usual controlled pore glass (CPG) support, the TG resin consisting of a polystyrene matrix with polyethylene glycol (PEG) linker grafted to it, was selected. Tentagel N is equally suitable for phosphoramidite chemistry as CPG but due to its swelling properties and loading capacity TG potentially allows for a bigger scale synthesis. A suitably protected ribose residue was obtained by a two step-conversion of D-ribose into a α/β mixture of 1-*O*-methyl-2,3,5-tri-*O*-benzyl-ribofuranoside (**S1**). The 2-*O*-benzyl in **S1** was selectively removed with $SnCl_4$/DCM[1] and the released hydroxyl was acetylated, after which the pure α-anomer **S2** could be isolated. The unexpected isomerization of β- to the desired α-anomer can be explained by the opening and closing of the furanose ring under the influence of Lewis acid ($SnCl_4$). Pd/C catalysed hydrogenolysis of the benzyl groups in **S2** was accompanied by acetyl migration to give **S3** as a mixture of regio-isomers. After protection of the primary OH in **S3** with the 4,4'-dimethoxytrityl (DMT) group to give **S4**, the Q-linker was introduced with EDC/TEA in pyridine. The obtained acid **S5** was used to functionalise Tentagel N solid supports **S6** to yield resins **S7** with desired loadings (207 μmol/g). Finally, the Fm-protected phosphotriester was installed by the following sequence of reactions. At first, the DMT group was removed with trichloroacetic acid (TCA) in DCM, then the released primary OH was phosphitylated with known bis-(*9H*-fluoren-9-ylmethyl)-diisopropylamidophosphite **19**[2,3] and, finally, the intermediate phosphite triester was oxidized by CSO to give **2**. The successful introduction of phosphate function was confirmed by ^{31}P-NMR analysis of the mixture obtained by NH_4OH treatment of **2**.

Part 2. Alternative route for the synthesis of parotriose 13

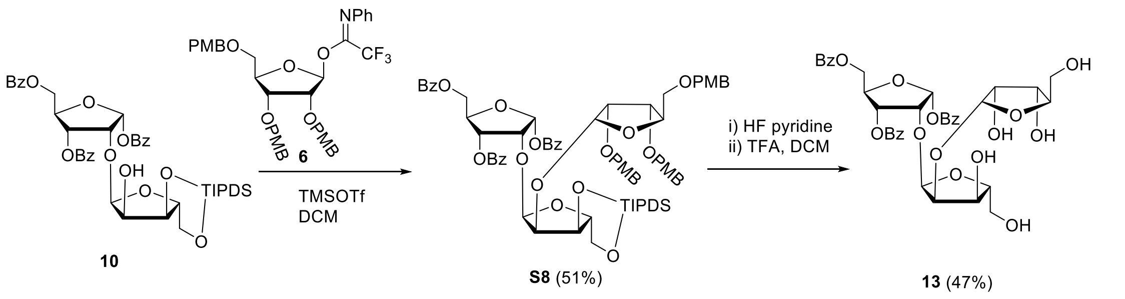

Supplementary Figure 16. Synthesis of parotriose **13** via the coupling of donor **6** and acceptor **10.**

Supplementary Figure 16 depicted the synthesis of parotriose **13**. Condensation of donor **6** and acceptor **10** using TMSOTf activation conditions gave trisaccharide **S8** with an excellent stereoselectivity (only α product), however only moderate yield. Subsequently, all PMB

protection and silyl groups needed to be removed for the upcoming manipulation of protecting groups. Unfortunately, pentol **13** was obtained with a yield of only 45% after HF-pyridine mediated desilylation and TFA assisted removal of the PMB groups. Changing the order of deprotection sequence did not improve the yield. The low yield could be attributed to acidic cleavage of one or both glycosidic bonds.

Part 3. Procedure for the synthesis of each compound

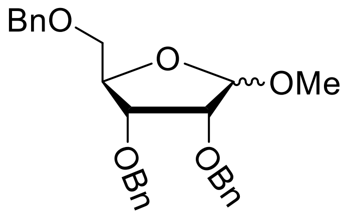

1-*O*-Methyl-2,3,5-tris-*O*-benzyl-αβ-D-ribofuranoside (S1)

D-Ribose(5 g, 33.30 mmol), methanol (120 mL) and acyl chloride (0.62 mL, 10.99 mmol) were added into a flask and the reaction was stirred at room temperature for 5 hours after which the reaction was quenched by $NaHCO_3$ (6 g). The mixture was filtered and concentrated. The residue was then co-evaporated with toluene (thrice) and DMF (160 mL) was added into the flask. The mixture was cooled down to 0 ℃ then NaH (5.4 g, 166.5 mmol, 60% in mineral oil) was added. After gas generation ceased, BnBr (15 mL, 166.5 mmol) was added in 3 portions over 10 min. The mixture was allowed to warm up to room temperature carefully and stirred for 16 h. MeOH (15 mL) was added to quench the reaction and H_2O and EtOAc were added. The water layer was washed with EtOAc then all the organic layers were combined and dried ($MgSO_4$). The mixture was filtered, concentrated and purified by silica gel column chromatography (pentane/EtOAc, 19/1 – 2/1) to obtain **S1** as a colourless oil (14.5 g, 33.30 mmol, 100%). Spectroscopic data was identical with the reported same compound.[4]

^{1}H NMR (500 MHz, chloroform-*d*) δ 7.44 – 7.12 (m, 20H, arom. αβ), 4.91 (d, *J* = 1.2 Hz, 1H, H1-β), 4.87 (d, *J* = 4.3 Hz, 0.3H, H1-α), 4.69 – 4.39 (m, 8H, CH_2-Bn-αβ), 4.35 (ddd, *J* = 7.1, 5.8, 3.7 Hz, 1H, H4-β), 4.27 – 4.21 (m, 0.3H, H4-α), 4.02 (dd, *J* = 7.1, 4.7 Hz, 1H, H3-β), 3.86 – 3.80 (m, 1.3H, H2-β, H3-α), 3.77 (dd, *J* = 6.8, 4.3 Hz, 0.3H, H2-α), 3.60 (AB, *J* = 10.6, 3.8 Hz, 1H, H5-β), 3.51 (AB, *J* = 10.6, 5.8 Hz, 1H, H5-β), 3.46 (s, 1H, OMe-α), 3.40 (AB, *J* = 10.4, 4.1 Hz, 0.3H, H5-α), 3.34 (AB, *J* = 10.4, 4.2 Hz, 0.3H, H5-α), 3.30 (s, 3H, OMe-β).

^{13}C NMR (126 MHz, $CDCl_3$) δ 138.37, 138.31, 137.98, 137.89, 137.87 (Cq. arom. αβ), 128.44, 128.41, 128.39, 128.35, 128.34, 128.31, 128.05, 127.99, 127.95, 127.91, 127.84, 127.80, 127.71, 127.69, 127.67, 127.63, 127.61, 127.54 (arom. αβ), 106.40 (C1-β), 102.53 (C1-α), 82.15(C4-α), 80.51 (C4-β), 79.75 (C2-β), 78.44 (C3-β), 77.86 (C2-α), 75.03 (C3-α), 73.48, 73.19, 72.47, 72.45, 72.35, 72.34 (CH_2 Bn-αβ), 71.37 (C5-β), 70.19 (C5-α), 55.57 (OMe-α), 55.09 (OMe-β).

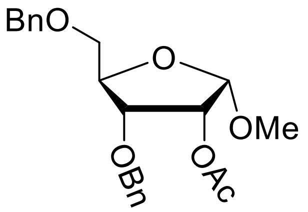

1-*O*-Methyl-2-*O*-acetyl-3,5-di-*O*-benzyl-α-D-ribofuranoside (S2) Compound **S1** (8.3 g, 19.10 mmol) and DCM (95 mL) were added into a flask after which the solution was cooled down to 0 ℃. $SnCl_4$ (19.1 mL, 19.1 mmol, 1M solution in DCM) was added to the reaction and the mixture was stirred at 4 ℃ for 16 hours. The reaction was quenched by aq. $NaHCO_3$ (sat.) and filtered. The organic filtration was washed by H_2O (once), brine (once) and dried ($MgSO_4$). The mixture was filtered, concentrated and co-evaporated with toluene (thrice). The residue was re-dissolved in pyridine (95 mL), added DMAP (117 mg, 0.96 mmol) and acetic anhydride (18.0 mL, 191.0 mmol). The reaction was stirred at room temperature for 3 hours after which was quenched by aq. $NaHCO_3$ (sat.). EtOAc was added to extract the mixture and the organic layer was further washed by H_2O (once) and brine (once). The organic layer was dried ($MgSO_4$), filtered and concentrated. The residue was purified by silica gel column chromatography (pentane/EtOAc, 95/5 – 80/20 – 70/30) to obtain **S2** as a colourless oil (6.4 g, 16.57 mmol, 87 %).

^{1}H NMR (500 MHz, chloroform-d) δ 7.40 – 7.22 (m, 10H), 5.12 (d, *J* = 4.5 Hz, 1H, H1), 4.91 (dd, *J* = 7.1, 4.5 Hz, 1H, H2), 4.68 (d, *J* = 12.4 Hz, 1H, C*H*H Bn), 4.53 (d, *J* = 12.1 Hz, 1H, C*H*H Bn), 4.47 (dd, *J* = 12.3, 10.4 Hz, 2H, 2xC*H*H Bn), 4.21 (d, J = 4.0 Hz, 1H, H4), 4.04 (dd, *J* = 7.1, 4.2 Hz, 1H, H3), 3.51 – 3.43 (m, 4H, OMe, H5), 3.33 (AB, *J* = 10.5, 4.2 Hz, 1H, H5), 2.20 (s, 3H, Ac).

^{13}C NMR (126 MHz, $CDCl_3$) δ 170.22 (CO Ac), 137.75, 137.74 (Cq. arom.), 128.21, 128.17, 127.97, 127.67, 127.50 (arom.), 101.64 (C1), 81.36 (C4), 75.01 (C3), 73.23, 72.90 (CH_2 Bn), 71.99 (C2), 69.25 (C5), 55.40 (OMe), 20.61 (Me Ac). IR (film): 2928, 1740, 1453, 1372, 1238, 1124, 1096, 1065, 1027, 739, 698 cm^{-1}.

HRMS (ESI$^+$) calcd for $C_{22}H_{26}O_6Na$ (M+Na) 409.1622. Found 409.1622. $[\alpha]_D^{20}$ +95.0 (c = 1, in DCM)

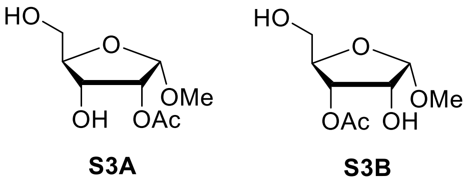

1-*O*-Methyl-2-*O*-acetyl-α-D-ribofuranoside (S3A)/
1-*O*-Methyl-3-*O*-acetyl-α-D-ribofuranoside (S3B)

Compound **S2** (1.24 g, 3.21 mmol) was dissolved in tBuOH/Dioxane/H_2O (12 mL, 4/4/1; v/v/v). Pd/C (124 mg, 10 wt % Pd) were added and H_2 bubbled through the mixture for 24 hours at room temperature after which the reaction mixture was filtered over celite. The filtration was concentrated under reduced pressure and co-evaporated with toluene (once). The residue was purified by silica gel column chromatography (DCM/methanol, 100/2 – 100/5) to obtain **S3** as a colourless oil (615 mg, 2.98 mmol, 93 %; 80% 2-OAC product and 20% 3-OAc product)

^{1}H NMR (500 MHz, chloroform-*d*) δ 5.10 (d, *J* = 4.1 Hz, 1H, H1-A), 4.96 (dd, *J* = 7.2, 3.3 Hz, 0.25H, H3-B), 4.92 (d, *J* = 4.6 Hz, 0.25H, H1-B), 4.75 (dd, *J* = 6.3, 4.1 Hz, 1H, H2-A), 4.19 (td, *J* = 7.2, 6.8, 3.5 Hz, 1H, H3-AB), 4.13 (td, *J* = 3.6, 2.4 Hz, 1H, H4-A), 4.05 (q, *J* = 3.4 Hz, 0.25H, H4-B), 3.81 – 3.73 (m, 1.5H, H5-A, H5-B), 3.69 (AB, *J* = 11.5, 3.4 Hz, 1H, H5-A), 3.45 (s, 0.75H, OMe-B), 3.41 (s, 3H, OMe-A), 2.84 (d, *J* = 9.6 Hz, 1H, 3-OH-A), 2.74 (d, *J* = 10.8 Hz, 0.25H, 3-OH-B), 2.40 (bs, 1.25H, 5-OH-AB), 2.15 (s, 3H, Ac-A), 2.11 (s, 0.75H, Ac-B).
^{13}C NMR (126 MHz, $CDCl_3$) δ 170.92, 170.47 (CO, Ac), 102.57 (C1-B), 101.96 (C1-A), 86.03 (C4-A), 83.07 (C4-B), 73.24 (C2-A), 71.49 (C3-B), 71.35 (C2-B), 70.04 (C3-A), 62.65 (C5-A), 62.51 (C5-B), 55.57 (OMe-B), 55.37 (OMe-A), 21.00 (CH_3 Ac-B), 20.77 (CH_3 Ac-A).
IR (film): 3444, 2932, 1735, 1374, 1234, 1081, 1028, 964, 899, 5002, 607, 479 cm^{-1}.
HRMS (ESI^{+}) calcd for $C_8H_{14}O_6Na$ (M+Na) 229.0683. Found 229.0685. $[\alpha]_D^{20}$ +112.3 (c = 1, in DCM)

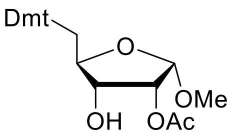

S4A

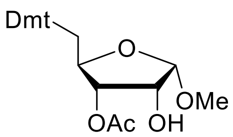

S4B

1-*O*-Methyl-2-*O*-acetyl-5-O-(4,4'-di-methoxyltrityl)-α-D-ribofuranoside (S4A) / 1-*O*-Methyl-3-*O*-acetyl-5-O-(4,4'-di-methoxyltrityl)-α-D-ribofuranoside (S4B)

Compound **S3** (584 mg, 2.83 mmol), 4,4'-dimethoxyltrityl chloride (DMTCl, 1.01 g, 2.98 mmol), and pyridine were added into a flask and the solution was stirred for 16 hours at room temperature after which was concentrated. The residue was dissolved in EtOAc and washed by aq. $NaHCO_3$ (sat.). The organic layer was dried ($MgSO_4$), filtered and concentrated. The residue was purified by silica gel column chromatography (pentane/EtOAc, 100/0 – 90/10 – 80/20 – 60/40) to obtain **S4** as a light yellow foam (1.31 g, 2.58 mmol, 91 %).
^{1}H NMR (400 MHz, chloroform-*d*) δ 7.46 – 7.42 (m, 2H, DMT arom.), 7.38 – 7.16 (m, 7H, DMT arom.), 6.88 – 6.80 (m, 4H, DMT arom.), 5.22 (d, *J* = 4.1 Hz, 0.25H, H1-B), 5.16 (dd, *J* = 7.0, 2.8 Hz, 0.75H, H3-A), 5.06 – 5.04 (m, 1H, H1-A, H2-B), 4.44 (dd, *J* = 7.0, 4.7 Hz, 0.75H, H2-A), 4.27 – 4.24 (m, 0.5H, H3-B, H4-B), 4.16 (q, *J* = 3.3 Hz, 0.75H, H4-A), 3.79 (s, 6H, OMe DMT), 3.51 (s, 2.25H, OMe-A), 3.47 (s, 0.75H, OMe-B), 3.39 – 3.33 (m, 1H, H5), 3.25 – 3.16 (m, 1H, H5), 2.72 (s, 1H, OH), 2.20 (s, 0.75H, Ac-B), 2.10 (s, 2.25H, Ac-A).
^{13}C NMR (101 MHz, $CDCl_3$) δ 170.44(CO Ac-A), 170.30 (CO Ac-B), 158.55, 144.83, 144.69, 136.03, 135.96, 135.87, 135.76 (Cq. arom.), 130.40, 130.13, 128.22, 127.91, 126.87, 113.21 (arom.), 102.59 (C1-A), 101.94(C1-B), 86.27, 86.26 (Cq. DMT), 85.50 (C4-B), 81.97 (C4-A), 73.34 (C2-B), 72.08 (C3-A), 71.42 (C2-A), 70.73 (C3-B), 63.68 (C5-A), 63.67 (C5-B), 55.70 (OMe), 55.34 (OMe DMT-B), 55.25 (OMe DMT-A), 21.01 (CH_3 Ac-A), 20.85 (CH_3 Ac-B).
IR (film): 3507, 2933, 2837, 1741, 1608, 1509, 1446, 1300, 1248, 1177, 1077, 1035, 830, 596 cm^{-1}.

HRMS (ESI⁺) calcd for $C_{29}H_{32}O_8Na$ (M+Na) 531.1989. Found 531.1992. $[\alpha]_D^{20}$ +53.1 (c = 1, in DCM)

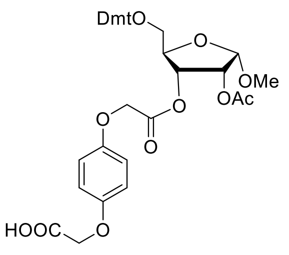

S5A

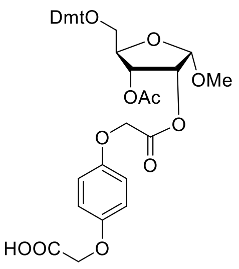

S5B

1-*O*-Methyl-2-*O*-acetyl-3-*O*-hydroquinone-*O,O'*-diacetylhemiester-5-O-(4,4'-dimethoxyltrityl)-α-ᴅ-ribofuranoside (S5A) / 1-*O*-Methyl-2-*O*-hydroquinone-*O,O'*-diacetylhemiester-5-*O*-(4,4'-dimethoxyltrityl)-3-*O*-acetyl-α-ᴅ-ribofuranoside (S5B)

Compound **S4** (1.22 g, 2.40 mmol) was dissolved in pyridine (12 mL). DMAP (29 mg, 0.24 mmol), EDC (446 mg, 2.88 mmol), Et_3N (0.24 mL, 1.73 mmol) and hydroquinone-O,O'-diacetic acid (Q-linker) (650 mg, 2.88 mmol) were added and the reaction was stirred at room temperature for 16 hours. The reaction mixture was concentrated, diluted with $CHCl_3$ and washed with H_2O. The water layer was extracted with $CHCl_3$ and the combined organic layers were dried ($MgSO_4$), concentrated and purified by silica gel chromatography neutralized with 1% Et_3N (DCM/methanol, 100/0 – 99/1 – 95/5 – 90/10) to obtain **S5** as a white foam (962 mg, 1.34 mmol, 56%).

^{1}H NMR (500 MHz, chloroform-*d*) δ 7.38 – 7.32 (m, 2H, arom.), 7.27 – 7.18 (m, 6H, arom.), 7.17 – 7.10 (m, 1H, arom.), 6.85 – 6.73 (m, 8H, arom.), 5.35 (dq, *J* = 7.5, 2.6 Hz, 1/3H, H3-B), 5.28 – 5.22 (m, 2/3H, H3-A), 5.19 (dd, *J* = 5.4, 3.7 Hz, 4/3H, H1-A, H2-A), 5.17 – 5.12 (m, 2/3H, H1-B, H2-B), 4.63 – 4.48 (m, 2H, CO*CH₂*O), 4.39 – 4.37 (m, 2H, *CH₂*COOH), 4.15 (q, *J* = 3.4 Hz, 1H, H4), 3.72 – 3.71 (m, 6H, OMe DMT), 3.38 (s, 3H, OMe), 3.33 – 3.29 (m, 1H, H5), 3.20 – 3.13 (m, 1H, H5), 2.01 (s, 1H, CH_3 Ac-B), 1.97 (s, 2H, CH_3 Ac-A).

^{13}C NMR (126 MHz, $CDCl_3$) δ 174.30(COOH-A), 174.28 (COOH-B), 170.49 (CO Ac-A), 170.03(CO Ac-B), 168.82(CH_2*CO*O-B), 168.45(CH_2*CO*O-A), 158.66, 158.65, 153.83, 152.15, 152.13, 144.75, 136.00, 135.96, 135.85, 135.78 (Cq. arom.), 130.21, 130.19, 128.29, 128.01, 126.98, 126.97, 116.00, 115.91, 115.71, 113.31 (arom.), 101.66 (C1-B), 101.64 (C1-A), 86.47 (Cq. DMT-B), 86.43(Cq. DMT-A), 81.21 (C4-B), 81.03 (C4-A), 71.78 (C2-A), 71.64 (C3-B), 71.42 (C2-B), 70.60 (C3-A), 67.62 (*CH₂*COOH), 66.25 (*CH₂*COO-B), 66.05 (*CH₂*COO-A), 63.45 (C5-B), 63.41 (C5-A), 55.88 (OMe-A), 55.76 (OMe-B), 55.36 (OMe DMT), 20.93 (CH_3 Ac-A), 20.75 (CH_3 Ac-B).

IR (film): 2934, 1738, 1607, 1507, 1445, 1246, 1178, 1073, 1032, 828cm^{-1}.

HRMS (ESI⁺) calcd for $C_{39}H_{40}O_{13}Na$ (M+Na) 739.2361. Found 739.2364. $[\alpha]_D^{20}$ +33.0 (c = 1, in DCM)

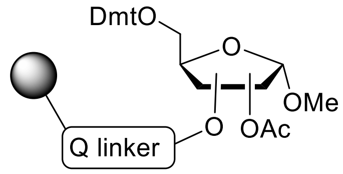

1-*O*-Methyl-2-*O*-acetyl-3-*O*-Q-TG-5-*O*-(4,4'-di-methoxyltrityl)-α-ribofuranoside/ 1-*O*-Methyl-2-*O*-Q-TG-3-*O*-acetyl-5-*O*-(4,4'-di-methoxyltrityl)-α-ᴅ-ribofuranoside (S7)

To a 20 mL reaction syringe with filter frit was added Tentagel N NH_2 (**S6**, 800 mg, 0.20 mmol), MeCN (12 mL), compound **S5** (430 mg, 0.6 mmol), HOBT (12 mg, 0.08 mmol), DIC (0.28 mL, 1.8 mmol) and DIPEA (0.52 mL, 3 mmol). The mixture was shaken at room temperature for 16 hours. The reaction mixture was drained and Tentagel was washed with ACN (twice), DMF (twice) and DCM (thrice) under N_2. The remaining unmodified amine groups were capped by adding a mixture of CAP 1 (6 mL) and CAP 2 (6 mL). The mixture was shaken for 2 hours, drained and washed with DMF (thrice) and DCM (thrice) under N_2. The Tentagel was dried under reduced pressure and the loading was determined by trityl analysis at 503 nm. The loadings for **S7** is 207 µmmol/g.

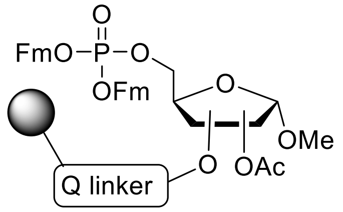

1-*O*-Methyl-2-*O*-acetyl-3-*O*-Q-TG-5-*O*-(di-*O*-fluorenylmethylphosphoryl)-α-ᴅ-ribofuranoside/ 1-*O*-Methyl-2-*O*-Q-TG-3-*O*-acetyl-5-*O*-(di-*O*-fluorenylmethylphosphoryl)-α-ᴅ-ribofuranoside (2)

To a 20 mL reaction syringe with filter frit was added **S7** (900 mg). dichloroacetic acid (5 %, v/v, in DCM) repeatedly until no yellow colour was observed. The resin was extensively washed with DCM (thrice), ACN (five times) under N_2. ETT (0.25M in ACN, 12 eq) and **19** (0.2 M in ACN, 4 eq) were added into the resin and the mixture was shaken under N_2 for 10 minutes and drained. This coupling was repeated once more, the resin drained and washed with ACN (five times). 9 mL of 0.5 M (1S)-(+)-(10-camphorsulfonyl)-oxaziridine (CSO) in ACN was added and the mixture shaken for 30 min under N_2. The resin was drained, washed with ACN (five times) under N_2 and dried under reduced pressure to obtain **2** which was stored at 4 ℃ before use. A test sample of **2** (20 mg) was added into a 2 mL reaction syringe. To this syringe 1 mL DBU solution (10%, v/v, in ACN) was added and the solution was shaken for 10 min to remove the 5-phosphate Fm groups. Subsequently, the TG was drained and washed with ACN (thrice). The resin was treated with 1mL NH_4OH (35%) for 1 hour to cleave the product from resin. The solvent was removed via filtration, followed by concentration under reduced pressure.

^{31}P NMR (162 MHz, D_2O): δ 4.57 (s)

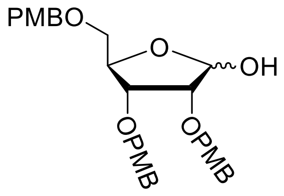

2,3,5-tri-*O*-*p*-methoxylbenzyl-D-ribofuranose (5)

D-Ribose (6.0 g, 40 mmol), allyl alcohol (100 mL) and acetyl chloride (2.0mL, 28 mmol) were added into a flask and the mixture was stirred for 2.5 hours after which the reaction was quenched with solid $NaHCO_3$ and filtered over celite. The filtration was concentrated under reduced pressure and co-evaporated with toluene (twice). To the residue, DMF (100 mL) was added and cooled down to 0 ℃. NaH (60% in mineral oil, 6.4 g, 160 mmol) was added in 3 portions. After hydrogen generation ceased, 4-methoxybenzyl chloride (19 mL, 14 mmol) was added dropwise at 0℃ under N_2. The mixture was stirred overnight after which it was quenched by aqueous saturated NH_4Cl. EA and H_2O were added into the mixture and the organic layer was washed additionally by H_2O (twice) and brine (once) and dried by $MgSO_4$. The mixture was concentrated under reduced pressure. To the residue, $CHCl_3$ (120 mL), H_2O (80 mL) and $PdCl_2$ (2.12 g, 12 mmol) were added and the mixture was vigorously stirred at 50 ℃ under O_2 atmosphere for 48 hours after which the mixture was concentrated under reduced pressure. EA (50 mL) and saturated aqueous $NaHCO_3$ (50 mL) were added into the residue and the organic layer was separated, dried with $MgSO_4$, filtered and concentrated under reduced pressure. Silica gel column chromatography (pentane/EA, 100/0 – 90/10 – 70/30) furnished **5** as light brown oil (11.44 g, 22.42 mmol, 56 %, α-product: 60%, β-product: 40%). ^{1}H-NMR and ^{13}C-NMR showed identical date with reported **5**.[5]

^{1}H NMR (500 MHz, chloroform-*d*) δ 7.35 – 7.10 (m, 6H, arom.), 6.94 – 6.73 (m, 6H, arom.), 5.27 – 5.28 (m, 1H, H1), 4.67 – 4.37 (m, 5.6H, CH_2 PMB), 4.32 – 4.24 (m, 1.4H, H4, C*H*H PMB), 4.19 – 4.15 (m, 1.4H, OH, H3-β), 3.95 – 3.89 (m, 1.2H, H3-α, H2-β), 3.83 – 3.77 (m, 9.4H, OMe, H2-β), 3.61 (AB, *J* = 10.3, 2.8 Hz, 0.4H, H5-β), 3.47 – 3.37 (m, 1.6H, H5-αβ).

^{13}C NMR (126 MHz, $CDCl_3$) δ 159.55, 159.50, 159.49, 159.48, 159.37, 130.12, 130.02, 129.98 (cq. arom.), 129.82, 129.75 (arom.), 129.71 (Cq. arom.), 129.69, 129.68, 129.56 (arom.), 129.51(Cq. arom.), 129.34, 114.00, 113.97, 113.93, 113.91, 113.90 (arom.), 100.52 (C1-β), 96.38 (C1-α), 81.10 (C4-α), 81.08 (C4-β), 80.55 (C2-β), 77.48 (C2-α), 77.36 (C3-α), 76.89 (C3-β), 73.27, 72.48, 72.20, 72.15, 72.03 (CH2 PMB), 69.82 (C5-α), 69.16 (C5-β), 55.40 (OMe-α), 55.38 (OMe-β).

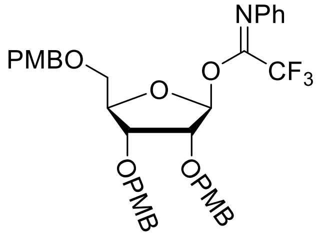

1-*O*-((*N*-Phenyl)-2,2,2-trifluoroacetimido)-2,3,4-tri-*O*-*p*-methoxylbenzyl-D-ribofuranose (6)

Compound **5** (9.3 g, 18.23 mmol) was dissolved in acetone (93 mL). Cs_2CO_3 (8.89 g, 27.34 mmol,) and 2,2,2-trifluoro-*N*-phenylacetimidoyl chloride (3.23 mL, 20.05 mmol) were added and the reaction mixture was stirred at room temperature for 3 hours. After filtration over celite, the solvent was removed and the residue purified using silica gel

column chromatography neutralised with 1% Et_3N (Pentane/EA, 100/0 – 90/10 – 80/20) to afford **6** as a light yellow oil (11.67 g, 17.13 mmol, 94 %).

^{1}H NMR (500 MHz, chloroform-*d*) δ 7.35 – 7.04 (m, 9H, arom.), 6.89 – 6.75 (m, 8H, arom), 6.27 (s, 1H, H1), 4.77 – 4.31 (m, 7H, CH_2 PMB, H4), 4.08 (t, *J* = 6.1 Hz, 1H, H3), 4.01 (d, *J* = 4.6 Hz, 1H, H2), 3.82 – 3.76 (m, 9H, OMe), 3.68 – 3.65 (m, 1H, H5), 3.54 (AB, *J* = 11.2, 5.2 Hz, 1H, H5).

^{13}C NMR (126 MHz, $CDCl_3$) δ 159.60, 159.54, 159.28, 143.97, 130.42 (Cq. arom.), 129.96 (arom.), 129.76 (Cq. arom.), 129.66 (arom.), 129.58 (arom.), 129.53 (Cq. arom.), 129.34, 129.32, 128.85, 113.96, 113.92, 113.84, 113.80 (arom.), 102.65 (C1), 82.34 (C4), 78.17 (C2), 77.05 (C3), 73.27, 73.04, 72.42, 72.22, 72.01 (CH_2 PMB-αβ), 69.89, 69.54 (C5-αβ), 55.40, 55.39 (OMe, αβ).

IR (film): 2935, 2837, 1709, 1612, 1512, 1302, 1246, 1205, 1156, 1110, 1033, 819, 755, 695, 515 cm^{-1}.

HRMS (ESI$^+$) calcd for $C_{29}H_{34}O_8Na$ (M+Na) 533.2146. Found 533.2147. $[\alpha]_D^{20}$ +41.6 (c = 1, in DCM)

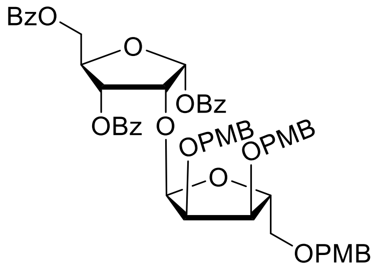

α-1,3,5-Tri-*O*-benzoyl-2',3',5'-tri-*O*-*p*-methoxylbenzyl-parobiose (8)

Compound **6** (5.9 g, 8.66 mmol) and α-D-ribofuranose 1,3,5-tribenzoate **7** (3.64 g, 7.87 mmol) were co-evaporated with toluene (once), 1,4-dioxane (twice) and DCE (once), dissolved in dry DCM and stirred with freshly activated 3 Å molecular sieves at room temperature for 1 hour under N_2 to remove traces of water. The solution was then cooled to -78 ℃ and TMSOTf (28 µL, 0.16 mmol) was added to the reaction mixture. The reaction was being stirred at the same temperature for 10 minutes after which it was quenched by the addition of triethylamine. The reaction mixture was concentrated under reduced pressure and purified by silica gel chromatography (DCM/EtOAc, 97/3 – 95/5) to afford **8** as a white foam (7.51 g, 7.87 mmol, 100%).

^{1}H NMR (400 MHz, chloroform-*d*) δ 8.17 – 8.14 (m, 4H, arom.), 8.10 – 8.02 (m, 2H, arom.), 7.58 – 7.46 (m, 3H, arom.), 7.44 – 7.40 (dd, *J* = 8.2, 7.0 Hz, 2H, arom.), 7.33 – 7.19 (m, 4H, arom.), 7.14 – 7.07 (m, 2H, arom.), 7.03 – 6.94 (m, 4H, arom.), 6.83 – 6.80 (m, 2H, arom., H1'), 6.69 – 6.58 (m, 4H, arom.), 5.68 (dd, *J* = 6.4, 1.9 Hz, 1H, H3'), 5.29 (d, *J* = 4.0 Hz, 1H, H1''), 4.77 – 4.70 (m, 2H, H4', H2'), 4.70 – 4.55 (m, 2H, H5'), 4.52 – 4.16 (m, 7H, CH_2 PMB, H4''), 3.89 – 3.79 (m, 2H, H2'', H3''), 3.79 (s, 3H, OMe), 3.73 (s, 3H, OMe), 3.72 (s, 3H, OMe), 3.42 (AB, *J* = 10.8, 3.3 Hz, 1H, H5''), 3.31 (AB, *J* = 10.8, 3.7 Hz, 1H, H5'').

^{13}C NMR (101 MHz, $CDCl_3$) δ 166.32, 166.12, 165.76 (CO Bz), 159.19, 158.99, 158.96 (Cq. arom.), 133.24, 133.16 (Cq. arom.), 130.33 (arom.), 130.18 (Cq. arom.), 130.04, 130.00,

129.87 (arom.), 129.75 (Cq. arom.), 129.64 (arom.), 129.29, 129.22, 128.99, 128.53, 128.35, 128.31, 113.73, 113.53, 113.50 (arom.), 102.01 (C1''), 95.12 (C1'), 83.38 (C4'), 81.67 (C4''), 77.60 (C2''), 75.44 (C2'), 75.27 (C3''), 72.98 (CH_2 PMB), 72.24 (C3'), 72.07 (CH_2 PMB), 71.70 (CH_2 PMB), 69.05 (C5''), 64.35 (C5'), 55.25, 55.22, 55.21 (OMe).

IR (film): 2934, 1721, 1612, 1513, 1451, 1266, 1248, 1175, 1111, 1068, 1026, 820, 710, 516 cm^{-1}.

HRMS (ESI$^+$) calcd for $C_{55}H_{54}O_{15}Na$ (M+Na) 977.3355. Found 977.3357. $[\alpha]_D^{20}$ +84.0 (c = 1, in DCM)

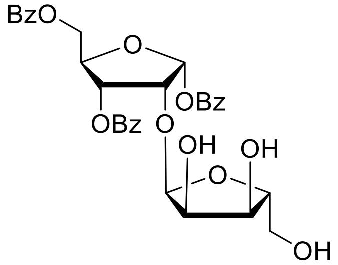

α-1,3,5-tri-*O*-benzoylparobiose (9)

Compound **8** (7.0 g, 7.34 mmol), DCM (60 mL) and TFA (3.37 mL, 44 mmol) were added into a flask and the reaction was stirred for 90 minutes after which was quenched by saturated aqueous $NaHCO_3$. DCM extracted the mixture (four times) and the organic layers were combined, dried ($MgSO_4$) and filtered. The filtration was concentrated under reduced pressure and purified by silica gel chromatography (DCM/acetone, 100/1 – 100/7 – 90/10 – 80/20) to furnish **9** as a white foam (2.61 g, 4.40 mmol, 64%).

^{1}H NMR (500 MHz, chloroform-*d*) δ 8.13 – 8.03 (m, 6H, arom.), 7.63 – 7.55 (m, 3H, arom.), 7.47 (t, *J* = 7.8 Hz, 2H, arom.), 7.43 – 7.33 (m, 4H, arom.), 6.76 (d, *J* = 4.2 Hz, 1H, H1'), 5.74 (dd, *J* = 6.3, 2.0 Hz, 1H, H3'), 5.19 (d, *J* = 4.2 Hz, 1H, H1''), 4.87 (td, *J* = 3.8, 1.9 Hz, 1H, H4'), 4.75 (dd, *J* = 6.3, 4.2 Hz, 1H, H2'), 4.63 (AB, *J* = 12.1, 3.8 Hz, 2H, H5'), 4.00 – 3.97 (m, 2H, H2'', H4''), 3.86 (s, 1H, H3'), 3.64 (AB, *J* = 12.2, 3.1 Hz, 1H, H5''), 3.55 (AB, *J* = 12.1, 3.9 Hz, 1H, H5''), 2.73 (s, 1H, OH), 2.56 (s, 1H, OH), 2.04 (s, 1H, OH).

^{13}C NMR (126 MHz, $CDCl_3$) δ 166.82, 166.14, 165.92 (CO Bz), 133.94, 133.66, 133.57, 130.04, 130.03, 129.79 (arom.), 129.66, 129.51, 129.05 (Cq. arom.), 128.71, 128.57 (arom.), 102.26 (C1''), 95.31 (C1'), 86.38 (C4''), 82.77 (C4'), 75.38 (C2'), 72.30 (C2''), 72.20 (C3'), 70.51 (C3''), 64.21 (C5'), 62.56 (C5'').

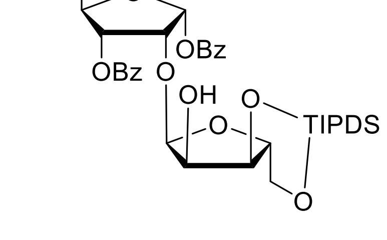

α-1,3,5-tri-*O*-benzoyl-3',5'-*O*-(1,1,3,3-tetraisopropyldisiloxane-1,3-diyl)-parobiose (10)

Compound **9** (2.48 g, 4.17 mmol) and imidazole (852 mg, 12.52 mmol) were co-evaporated with toluene (twice), dissolved in DCM (41 mL) and then $TIPDSCl_2$ (1.6 mL, 5.01 mmol) was added. The reaction was stirred at room temperature for 16 hours and quenched by addition of H_2O (200 mL). The mixture was washed by DCM (thrice) and the organic layer was dried ($MgSO_4$),

filtered and concentrated under reduced pressure. The residue was purified by silica gel chromatography (DCM/acetone, 100/0 – 97/3) to obtain **10**[6] as colourless foam (2.75 g, 3.29 mmol, 79%).

^{1}H NMR (500 MHz, chloroform-*d*) δ 8.20 – 8.02 (m, 6H, arom.), 7.65 – 7.53 (m, 3H, arom.), 7.53 – 7.44 (m, 2H, arom.), 7.44 – 7.35 (m, 4H, arom.), 6.79 (d, *J* = 4.2 Hz, 1H, H1’), 5.67 (dd, *J* = 6.4, 2.1 Hz, 1H, H3’), 5.19 (d, *J* = 4.2 Hz, 1H, H1”), 4.81 – 4.73 (m, 2H, H2’, H4’), 4.73 – 4.58 (m, 2H, H5’), 4.12 – 4.05 (ddd, *J* = 19.8, 7.7, 4.6 Hz, 2H, H2”, H3”), 3.93 (ddd, *J* = 8.4, 5.0, 3.5 Hz, 1H, H4”), 3.82 (dd, *J* = 11.7, 3.6 Hz, 1H, H5”), 3.66 (AB, *J* = 11.7, 8.3 Hz, 1H, H5”), 2.84 (d, *J* = 8.6 Hz, 1H, OH), 1.13 – 0.83 (m, 24H, CH_3 TBDPS), 0.80 (d, *J* = 7.3 Hz, 2H, CH TIPDS), 0.74 (d, *J* = 7.3 Hz, 2H, CH TIPDS).

^{13}C NMR (126 MHz, $CDCl_3$) δ 166.15, 165.70 (CO Bz), 133.49, 133.46, 133.43, 130.16, 129.99, 129.82 (arom.), 129.71 (Cq. arom.), 128.67, 128.57, 128.49 (arom.), 101.96 (C1”), 95.14 (C1’), 83.87 (C4”), 83.32 (C4’), 75.67 (C2’), 71.95 (C3’), 71.08 (C2”), 70.84 (C3”), 64.31 (C5’), 63.50 (C5”), 17.55, 17.49, 17.46, 17.41, 17.07, 16.98, 16.83, 16.69, 13.46, 13.27, 13.04, 12.38 (CH, CH_3 TIPDS).

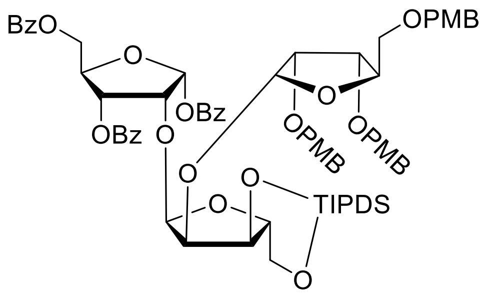

α-1,3,5-tri-*O*-benzoyl-3’,5’-*O*-(1,1,3,3-tetraisopropyldisiloxane-1,3-diyl)-2’’,3’’,5’’-di-*O*-*p*-methoxylbenzyl-parobiose (S8)

Compound **10** (1.43 g, 1.71 mmol) and **6** (1.40 g, 2.05 mmol) were co-evaporated with toluene (twice), 1,4-dioxane (twice) and DCE (once), dissolved in dry DCM and stirred with freshly activated 3 Å molecular sieves at room temperature for 1 hour under N_2 to remove traces of water. The solution was then cooled to -78 ℃ and TMSOTf (10 µL, 0.05 mmol) was added to the reaction mixture. The reaction was stirred at the same temperature for 30 minutes after which it was quenched by the addition of triethylamine. The reaction mixture was concentrated under reduced pressure and purified by silica gel chromatography (pentane/EtOAc, 100/0 – 90/10 – 80/20) to afford **S8** as a white foam (1.16 g, 0.87 mmol, 51%).

^{1}H NMR (500 MHz, chloroform-*d*) δ 8.18 – 8.11 (m, 2H, arom.), 8.07 (d, *J* = 6.9 Hz, 4H, arom.), 7.61 – 7.51 (m, 2H, arom.), 7.47 (td, *J* = 7.6, 3.9 Hz, 3H, arom.), 7.35 (t, *J* = 7.8 Hz, 2H, arom.), 7.27 – 7.16 (m, 4H, arom.), 7.12 – 7.02 (m, 4H, arom.), 6.90 – 6.67 (m, 7H, arom. H1’), 5.65 (dd, *J* = 6.2, 1.8 Hz, 1H, H3’), 5.38 (d, *J* = 3.5 Hz, 1H, H1”), 5.32 (d, *J* = 3.8 Hz, 1H, H1’”), 4.85 (dd, *J* = 6.2, 4.1 Hz, 1H, H2’), 4.75 – 4.60 (m, 3H, H4’, H5’, C*H*H PMB), 4.54 (AB, *J* = 12.0, 4.0 Hz, 1H, H5’), 4.48 – 4.45 (m, 2H, H2”, C*H*H PMB), 4.34 (dd, *J* = 32.6, 11.5 Hz, 2H, C*H2*, PMB), 4.26 – 4.00 (m, 4H, H4’”, H3”, C*H*H PMB, H4”), 3.89 – 3.67 (m, 12H, H5”,

C*H*H PMB, OMe PMB), 3.61 – 3.47 (m, 2H, H3''', H5'''), 3.47 – 3.33 (m, 2H, H2''', H5'''), 1.12 – 0.80 (m, 28H).

^{13}C NMR (126 MHz, CDCl$_3$) δ 166.04, 165.87, 165.59 (CO Bz), 159.14, 159.05, 158.84 (Cq. arom.), 133.45, 133.39, 133.24 (arom.), 131.05, 130.43, 130.38 (Cq. arom.), 130.08 (arom.), 129.92 (Cq. arom.), 129.88, 129.81 (arom.), 129.65, 129.57, 129.43, 129.26, 129.21, 128.55, 128.52, 128.48, 113.73, 113.70, 113.66, 113.61, 113.49, 113.32 (arom.), 102.31 (C1''), 101.30 (C1'''), 94.98 (C1'), 83.35 (C4'), 80.89 (C4''), 79.10 (C4'''), 76.30 (C2'''), 75.51 (C3'''), 75.31 (C2'), 73.24 (C2''), 72.96 (CH2 PMB), 72.28 (C3'), 72.07, 71.10 (CH2 PMB), 68.92 (C3''), 68.73 (C5'''), 64.20 (C5'), 59.82 (C5''), 55.23, 55.21, 55.16 (OMe), 17.42, 17.37, 17.32, 17.17, 17.08, 17.05, 16.94 (CH_3 TIPDS), 13.48, 13.03, 12.67, 12.51 (CH TIPDS).

IR (film): 2944, 2867, 1724, 1613, 1514, 1266, 1248, 1112, 1035, 711cm^{-1}.

HRMS (ESI$^+$) calcd for $C_{72}H_{92}NO_{20}Si_2$ (M+NH$_4$) 1346.5746. Found 1346.5753. $[\alpha]_D^{20}$ +77.6 (c = 1, in DCM)

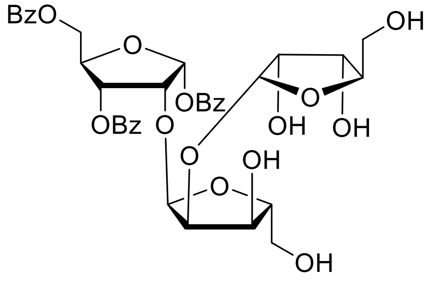

α-1,3,5-tri-*O*-benzoyl-2'',3''-di-*O*-benzylparotriose (13)

The procedure from **S8**:

Compound **S8** (430 mg, 0.32 mmol) and pyridine (3 mL) were added into a flask and cooled to 0 ℃. Subsequently, HF-pyridine (0.25 mL, 9.62 mmol) was added at the same temperature under N_2. The reaction was stirred at room temperature for 3 hours and quenched by the addition of saturated aqueous $NaHCO_3$. The mixture was extracted by DCM (thrice) and the organic layers are combined and dried ($MgSO_4$). The mixture was filtered, concentrated under reduced pressure and co-evaporated with toluene (thrice). To the residue, DCM (3 mL) and TFA (0.25 mL, 3.27 mmol) were added and the reaction was stirred for 20 minutes after which was quenched by addition of aqueous saturated $NaHCO_3$. The mixture was extracted by DCM (thrice), dried ($MgSO_4$), filtered and concentrated under reduced pressure. The residue was purified by silica gel chromatography (DCM/Methanol, 100/0 – 99/1 – 97/3 – 95/5 – 96/4) to obtain **13** as a white foam (106 mg, 0.15 mmol, 47%).

^{1}H NMR (500 MHz, chloroform-*d*) δ 8.17 – 7.97 (m, 6H, arom.), 7.63 – 7.50 (m, 3H. arom.), 7.50 – 7.32 (m, 6H, arom.), 6.76 (d, *J* = 4.1 Hz, 1H, H1'), 5.69 (dd, *J* = 6.3, 1.7 Hz, 1H, H3'), 5.27 (d, *J* = 4.0 Hz, 1H, H1''), 4.95 (d, *J* = 4.0 Hz, 1H, H1'''), 4.82 (td, *J* = 4.1, 1.6 Hz, 1H, H4'), 4.72 (dd, *J* = 6.3, 4.2 Hz, 1H, H2'), 4.61 (AB, *J* = 12.0, 4.1 Hz, 2H, H5'), 4.07 (dd, *J* = 5.8, 4.0 Hz, 1H, H2''), 3.98 – 3.93 (m, 3H, H3'', H4''', H4''), 3.72 – 3.35 (m, 7H, H3''', H2''', H5'', H5''', OH), 3.30 (s, 1H, OH), 3.22 – 3.08 (m, 1H, OH), 3.00 – 2.94 (m, 2H, OH).

^{13}C NMR (126 MHz, CDCl$_3$) δ 166.67, 166.20, 165.96 (CO Bz), 133.85, 133.77, 133.52, 130.16, 129.79 (arom.), 129.56, 129.54, 129.14 (Cq. arom.), 128.67, 128.58, (arom.) 101.44

(C1''), 101.05 (C1'''), 95.37 (C1'), 86.33 (C4''), 85.75 (C4'''), 83.21 (C4'), 75.34 (C2'), 75.29 (C2''), 72.46 (C2'''), 72.16 (C3'), 70.91 (C3''), 70.85 (C3'''), 64.31 (C5'), 62.78 (C5'''), 62.19 (C5'').

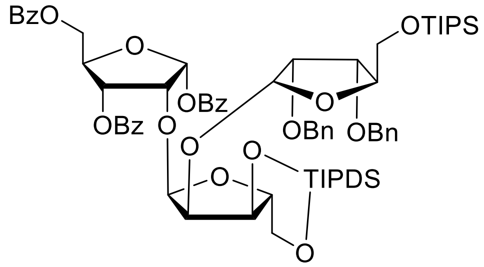

α-1,3,5-tri-*O*-benzoyl-3',5'-*O*-(1,1,3,3-tetraisopropyldisiloxane-1,3-diyl)-2'',3''-di-*O*-benzyl-5''-*O*-triisopropylsilylparotriose (12)

Compound **12** was synthesised according to our previously reported procedure with minor modification.[6] Compound **10** (4.7 g, 5.61 mmol) and **11** (5.17 g, 7.86 mmol) were co-evaporated with toluene (once), 1,4-dioxane (twice) and DCE (once). Dry DCM (94 mL) and freshly activated 3Å molecular sieves were added to the mixture. The reaction was stirred under N_2 at room temperature for 2 hours and then cooled to -78 ℃. Next, TMSOTf (30 uL, 0.17 mmol) was added, the reaction mixture was stirred at the same temperature for 15 minutes and subsequently quenched by addition of triethylamine. The reaction mixture was concentrated under reduced pressure and purified by silica gel chromatography (pentane/EA, 100/0 – 100/3 – 90/10) to obtain **12** as a white foam (6.58 g, 5.04 mmol, 90%).

^{1}H NMR (500 MHz, chloroform-*d*) δ 8.17 – 8.12 (m, 2H, arom.), 8.08 (ddd, *J* = 11.8, 8.4, 1.4 Hz, 4H, arom.), 7.59 – 7.53 (m, 2H, arom.), 7.52 – 7.43 (m, 3H, arom.), 7.36 (t, *J* = 7.8 Hz, 2H, arom.), 7.29 – 7.14 (m, 12H, arom.), 6.79 (d, *J* = 4.1 Hz, 1H, H1'), 5.60 (dd, *J* = 6.3, 1.9 Hz, 1H, H3'), 5.36 (d, *J* = 3.5 Hz, 1H, H1''), 5.27 (d, *J* = 3.8 Hz, 1H, H1'''), 4.84 (dd, *J* = 6.3, 4.1 Hz, 1H, H2'), 4.76 – 4.66 (m, 2H, CH_2 Bn, H4'), 4.62 (AB, *J* = 12.0, 3.4 Hz, 1H, H5'), 4.52 (AB, *J* = 12.0, 4.0 Hz, 1H, H5'), 4.46 – 4.43 (m, 1H, C*H*H Bn), 4.40 (dd, *J* = 5.3, 3.5 Hz, 1H, H2''), 4.32 (d, *J* = 11.8 Hz, 1H, CH_2, Bn), 4.22 – 4.14 (m, 2H, H3'', H4'''), 4.04 (dt, *J* = 8.8, 2.5 Hz, 1H, H4''), 3.97 (d, *J* = 11.4 Hz, 1H, C*H*H Bn), 3.82 (AB, *J* = 13.0, 2.4 Hz, 1H, H5''), 3.78 – 3.64 (m, 4H, H5'', H5''', H3'''), 3.44 (dd, *J* = 6.4, 3.8 Hz, 1H, C*H*H Bn), 1.13 – 0.85 (m, 49H, TIPS, TIPDS).

^{13}C NMR (126 MHz, $CDCl_3$) δ 166.14, 165.99, 165.64 (CO Bz), 138.98, 138.71 (Cq. arom.), 133.48, 133.45, 133.28, 130.14 (arom.), 130.06 (Cq. arom.), 130.00, 129.92 (arom.), 129.80, 129.70 (Cq. arom.), 128.61, 128.55, 128.53, 128.12, 127.99, 127.84, 127.68, 127.34, 127.14 (arom.), 102.22 (C1''), 101.25 (C1'''), 95.08 (C1'), 83.33 (C4'), 81.18 (C4'''), 81.16 (C4''), 77.38 (C2'''), 75.61 (C3'''), 75.14 (C2'), 73.61 (C2''), 72.34 (C3'), 72.30 (CH_2, Bn), 71.78 (CH_2, Bn), 69.09 (C3'''), 64.25 (C5'), 62.64 (C5''), 60.02 (C5'''), 18.06, 17.51, 17.46, 17.41, 17.40, 17.21, 17.16, 17.08, 16.95, 13.60, 13.13, 12.78, 12.60, 12.03 (CH_3, CH, TIPDS, TIPS).

α-1,3,5-tri-*O*-benzoyl-2'',3''-di-*O*-benzylparotriose (13)

The procedure from **12**:

Compound **12** (3.21 g, 2.46 mmol) was dissolved in *t*BuOH/dioxane/H_2O (50 mL, 4/4/1; v/v/v), Pd/C (500 mg, 10% loading) and one drop of AcOH were added. The mixture was sonicated under N_2 for 10 minutes then was transferred into an autoclave. The reaction in the autoclave was stirred for 16 hours under 80 bar of H_2 after which was filtered and concentrated under reduced pressure. The residue was co-evaporated with toluene (thrice) and pyridine (thrice). To the intermediate, pyridine (12 mL), triethylamine (5.14 mL, 36.91 mmol) and $Et_3N{\cdot}3HF$ (6.02 mL, 36.91 mmol) were added successively under 0 ℃. The reaction was allowed to warm up to room temperature and stirred for 16 hours after which it was quenched carefully by addition of saturated aqueous $NaHCO_3$. The product was DCM extracted (thrice), dried over $MgSO_4$ and purified by silica gel chromatography (DCM/Methanol, 100/0 – 95/5 – 90/10) to obtain **13**[6] as white foam (1.41 g, 1.94 mmol, 79%).

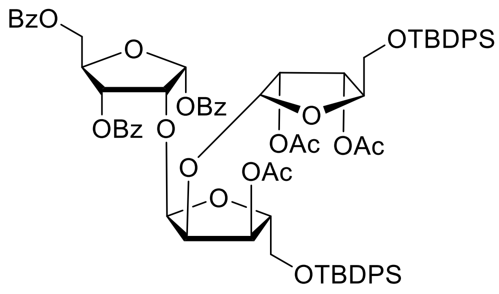

α-1,3,5-tri-*O*-benzoyl-3'-*O*-acetyl-5'-*O*-tertbutyldiphenylsilyl-2'',3''-di-*O*-acetyl-5''-*O*-tertbutyldiphenylsilylparotriose (14)

Compound **13** (2.36 g, 3.25 mmol) was co-evaporated with pyridine (once) and then N_2 was applied. Pyridine (32 mL) and TBDPSCl (2.54 mL, 9.75 mmol) were added and the mixture was stirred under N_2 at room temperature for 16 hours. Ac_2O (9.2 mL, 97.5 mmol) was added into the reaction and the mixture was stirred for 6 hours after which it was quenched by addition of saturated aqueous $NaHCO_3$. The mixture was extracted by DCM (thrice), dried ($MgSO_4$) and concentrated under reduced pressure. Purification by silica gel chromatography (pentane/actone, 100/0 – 90/10 – 80/20) gave **14**[6] as a white foam (3.54 g, 2.66 mmol, 82%).

^{1}H NMR (500 MHz, chloroform-*d*) δ 8.23 – 8.17 (m, 2H, arom.), 8.17 – 8.13 (m, 2H, arom.), 8.11 – 8.04 (m, 2H, arom.), 7.65 (dq, *J* = 6.5, 1.5 Hz, 4H, arom.), 7.63 – 7.50 (m, 7H, arom.), 7.45 – 7.30 (m, 18H, arom.), 6.80 (d, *J* = 4.3 Hz, 1H, H1'), 5.72 (dd, *J* = 6.3, 1.8 Hz, 1H, H3'), 5.46 (dd, *J* = 6.6, 1.9 Hz, 1H, H3''), 5.39 (dd, *J* = 7.0, 3.2 Hz, 1H, H3'''), 5.31 (d, *J* = 4.2 Hz, 1H, H1''), 5.29 (d, *J* = 4.4 Hz, 1H, H1'''), 4.88 (dd, *J* = 7.0, 4.4 Hz, 1H, H2'''), 4.76 (td, *J* = 3.8, 1.8 Hz, 1H, H4'), 4.71 – 4.58 (m, 3H, H2', H5'), 4.35 (dd, *J* = 6.6, 4.2 Hz, 1H, H2'''), 4.09 – 4.06 (m, 2H, H4''', H4''), 3.81 (AB, *J* = 11.2, 2.8 Hz, 1H, H5''), 3.69 (AB, *J* = 11.2, 3.1 Hz, 1H, H5''), 3.67 – 3.58 (m, 2H, H5'''), 2.01 (s, 3H, Ac), 1.78 (s, 3H, Ac), 1.63 (s, 3H, Ac), 1.05 (s, 9H, CH_3 TBDPS), 0.97 (s, 10H, CH_3, TBDPS).

^{13}C NMR (126 MHz, $CDCl_3$) δ 170.70, 170.10, 169.70 (CO, Ac), 166.18, 166.15, 165.57 (CO, Bz), 135.76, 135.74, 135.72, 135.68, 133.51, 133.49 (arom.), 133.16, 133.11, 133.04, 132.98, 130.28 (Cq. arom.), 130.17, 130.13 (arom.), 129.99 (Cq. arom.), 129.96, 129.94, 129.90,

129.86, 129.81 (arom.), 129.75 (Cq. arom.), 128.70, 128.51, 127.93, 127.91, 127.90, 127.88 (arom.), 101.29 (C1''), 99.53 (C1'''), 95.17 (C1'), 83.74 (C4''), 83.67 (C4'), 83.14 (C4'''), 76.35 (C2'), 74.63 (C2''), 71.76 (C2'''), 71.67 (C3'), 71.26 (C3''), 69.84 (C3'''), 64.45 (C5'), 63.92 (C5''), 63.43 (C5'''), 26.91, 26.86 (CH_3 TBDPS), 20.70, 20.39, 20.17 (Ac), 19.38, 19.30 (Cq. TBDPS).

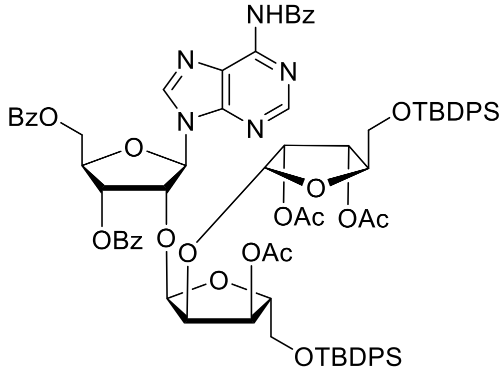

6-*N*-benzoyl-9-(3',5'-di-*O*-benzoyl-3''-*O*-acetyl-5''-*O*-tertbutyldiphenylsilyl-2''',3'''-di-*O*-acetyl-5'''-*O*-tertbutyldiphenylsilyl-β-parotriosyl)adenine (15)

Compound **14** (1.93 g, 1.48 mmol) and N^6-benzoyladenine (0.71 g, 2.97 mmol) were co-evaporated with 1,4-dioxane (twice), ACN (once) and dissolved in dry MeCN (24 mL) under N_2. N,O-bis(trimethylsilyl)trifluoroacetamide (BSTFA) (5.56 mL, 20.76 mmol) was added and the mixture was stirred at room temperature for 5 minutes. $HClO_4$-SiO_2 (7.4 g, 0.4 mmol/g, 2.96 mmol) was added and the mixture was refluxed for 16 hours. The reaction was quenched by aqueous saturated $NaHCO_3$ then filtered. The mixture was extracted with EtOAc (thrice), dried ($MgSO_4$) and concentrated under reduced pressure. Purification by silica gel chromatography (pentane/acetone, 100/0 – 85/15 – 80/20) gave **15**[6] as a white foam (1.82 g, 1.26 mmol, 85%).

^{1}H NMR (500 MHz, chloroform-*d*) δ 8.99 (s, 1H, NH), 8.68 (d, *J* = 1.8 Hz, 1H, H2), 8.40 (d, *J* = 1.9 Hz, 1H, H8), 8.07 (tt, *J* = 6.6, 1.6 Hz, 4H, arom.), 8.01 – 7.93 (m, 2H, arom.), 7.65 – 7.47 (m, 13H, arom.), 7.46 – 7.25 (m, 16H, arom.), 6.32 (dd, *J* = 4.7, 1.8 Hz, 1H, H1'), 5.94 (td, *J* = 5.4, 1.8 Hz, 1H, H3'), 5.68 (t, *J* = 5.0 Hz, 1H, H2'), 5.43 (dt, *J* = 6.9, 2.2 Hz, 1H, H3''), 5.41 – 5.37 (m, 1H, H3'''), 5.24 (dd, *J* = 4.4, 1.8 Hz, 1H, H1''), 5.16 (dd, *J* = 4.5, 1.8 Hz, 1H, H1'''), 4.93 (ddd, *J* = 7.2, 4.3, 1.7 Hz, 1H, H2'''), 4.91 – 4.85 (m, 1H, H5'), 4.75 (q, *J* = 4.6 Hz, 1H, H4'), 4.70 (AB, *J* = 11.9, 5.0 Hz, 1H, H5'), 4.31 (ddd, *J* = 6.8, 4.3, 1.8 Hz, 1H, H2''), 4.09 (t, *J* = 2.9 Hz, 1H, H4'''), 4.00 (q, *J* = 2.7 Hz, 1H, H4'), 3.79 – 3.76 (m, 1H, H5'''), 3.71 – 3.68 (m, 1H, H5'''), 3.59 – 3.56 (m, 1H, H5''), 3.45 – 3.41 (m, 1H, H5''), 2.10 (s, 3H, Ac), 2.07 (s, 3H, Ac), 1.68 (s, 3H, Ac), 1.01 (s, 9H, CH_3 TBDPS), 0.95 (s, 9H, TBDPS).

^{13}C NMR (101 MHz, $CDCl_3$) δ 170.57, 169.93, 169.83 (CO Ac), 166.30, 165.40, 164.49 (CO Bz), 152.94 (CH C2), 151.41 (Cq. arom.), 149.95 (CH C8), 149.80 (Cq. arom), 136.09, 135.67 (Cq. arom), 133.79 (Cq. arom), 133.63, 133.49 (arom.), 133.03, 132.98, 132.94 (Cq. arom.), 132.87 (arom.), 132.82 (Cq. arom.), 129.93, 129.90, 129.88 (aom.), 129.63, 129.60 (Cq. arom.), 128.97, 128.64, 128.59, 127.93, 127.91, 127.89 (arom.), 123.95 (Cq. arom.), 123.86 (arom.), 101.26 (C1''), 98.72 (C1'''), 89.14 (C1'), 83.11 (C4'''), 82.45 (C4''), 80.52 (C4'), 77.48 (C2'), 73.09 (C2''), 72.49 (C3'), 71.79 (C2'''), 71.02 (C3''), 69.86 (C3'''), 63.62 (C5'), 63.57

(C5’’), 63.19 (C5’’’), 26.87 (CH_3 TBDPS), 26.81 (CH_3 TBDPS), 20.77, 20.68, 20.43 (CH_3 Ac), 19.29, 19.26 (Cq. TBDPS).

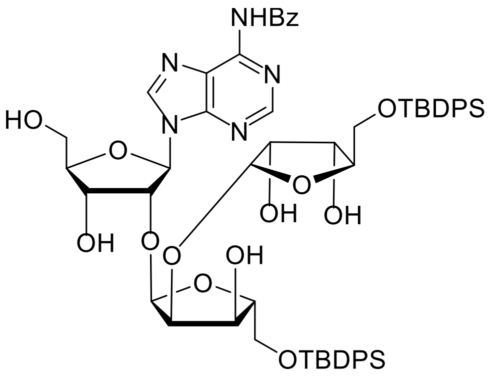

6-*N*-benzoyl-9-(5’’,5’’’-di-*O*-tertbutyldiphenylsilyl-β-parotriosyl)adenine (16)

Compound **15** (1.82 g, 1.26 mmol) was dissolved in pyridine/EtOH (12.6 mL; 2/1 v/v), cooled to 0 ℃ after which aqueous NaOH (7.56 mL, 1 M solution) was slowly added. The reaction mixture was stirred for 2 hours at the same temperature after which Amberlite-H^+ was added until the solution reached a pH of 6. The mixture was filtered, concentrated under reduced pressure and purified by silica gel chromatography (DCM/methanol, 100/0 – 97/3 – 95/5) to obtain **16**[6] as a white foam (1.26 g, 1.13 mmol, 90%).

^{1}H NMR (500 MHz, chloroform-*d*) δ 9.31 (s, 1H, NH), 8.82 (s, 1H, H2), 8.39 (s, 1H, H8), 8.02 – 7.95 (m, 2H, arom.), 7.67 – 7.54 (m, 8H, arom.), 7.48 (t, *J* = 7.8 Hz, 2H, arom.), 7.44 – 7.28 (m, 13H, arom.), 6.15 (d, *J* = 7.5 Hz, 1H, H1’), 6.07 (br, 1H, OH), 5.09 (d, *J* = 4.5 Hz, 1H, H1’’), 5.00 (d, *J* = 4.0 Hz, 1H, H1’’’), 4.94 (dd, *J* = 7.6, 4.6 Hz, 1H, H2’), 4.60 (d, *J* = 4.7 Hz, 1H, H3’), 4.41 (t, *J* = 4.8 Hz, 1H, H2’’), 4.39 – 4.33 (m, 2H, H3’’’, H4’), 4.31 – 4.19 (m, 4H, H2’’’, H3’’’, H4’’, H4’’’), 4.00 (AB, *J* = 13.0, 1.8 Hz, 1H, H5’), 3.78 (d, *J* = 12.8 Hz, 1H, H5’), 3.75 – 3.64 (m, 4H, H5’’, H5’’’), 1.00 (s, 9H, TBDPS), 1.00 (s, 9H, TBDPS).

^{13}C NMR (126 MHz, $CDCl_3$) δ 164.86 (CO Bz), 150.75, 150.45 (Cq. arom.), 135.69, 135.68, 135.66, 135.64 (arom.), 133.61, 133.09 (Cq. arom.), 133.05 (arom.), 132.89, 132.87, 132.71 (arom.), 130.09, 130.06, 130.04, 129.97, 129.00, 128.11, 127.99, 127.99, 127.97, 127.91 (arom.), 124.66 (Cq. arom.), 102.17 (C1’’’), 101.26 (C1’’), 89.74 (C1’), 88.39 (C4’), 86.56 (C4’’), 86.18 (C4’’’), 80.15 (C2’), 73.13 (C2’’’), 73.01 (C3’), 72.10 (C3’’), 71.34 (C3’’’), 64.36 (C5’’’), 64.19 (C5’’), 63.47 (C5’), 26.95 (CH3 TBDPS), 26.93 (CH_3 TBDPS), 19.34 (Cq. TBDPS), 19.30 (Cq. TBDPS).

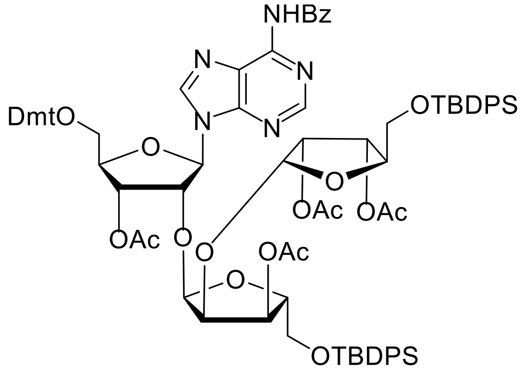

6-*N*-benzoyl-9-(3',3''2''',3'''-tetra-*O*-acetyl-5'-*O*-dimethoxyltrityl-5'',5'''-di-*O*-tertbutyldiphenylsilyl-β-parotriosyl)adenine (17)

Compound **16** (1.26 g, 1.13 mmol) was co-evaporated with pyridine (once), then N_2 was applied. Dry pyridine (5.6 mL) and 4,4'-dimethoxyltrityl chloride (DMTCl, 612 mg, 1.81 mmol) were added into the flask and the reaction was stirred at room temperature. 1 hour later, TLC showed incomplete conversion (DCM:methanol = 9.5:0.5 as eluent) and further DMTCl (153 mg, 0.45 mmol) was added. The reaction was stirred for 1 hour after which was cooled down to 0 ℃. Ac_2O (2.13 mL, 22.6 mmol) was added to the reaction flask. The mixture was stirred at 0 ℃ for 5 h after which was quenched by aqueous saturated $NaHCO_3$. DCM extracted (thrice) the mixture and the organic layers were combined, dried ($MgSO_4$), filtered, and concentrated under reduced pressure. Purification by silica gel column chromatography (pentane/acetone, 100/0 – 90/10 – 85/15 – 80/20 – 70/30) furnished **17** as a white foam (1.43 g, 0.90 mmol, 80%).

^{1}H NMR (400 MHz, chloroform-*d*) δ 8.97 (s, 1H, NH), 8.67 (s, 1H, H2), 8.38 (s, 1H, H8), 8.03 – 7.94 (m, 2H, arom.), 7.68 – 7.56 (m, 9H, arom.), 7.56 – 7.48 (m, 2H, arom.), 7.45 – 7.17 (m, 21H, arom.), 6.82 – 6.75 (m, 4H, DMT), 6.27 (d, *J* = 4.3 Hz, 1H, H1'), 5.57 – 5.47 (m, 2H, H3', H3''), 5.42 (dd, *J* = 7.3, 3.3 Hz, 1H, H3'''), 5.35 (t, *J* = 4.9 Hz, 1H, H2'), 5.27 (d, *J* = 4.4 Hz, 1H, H1''), 5.25 (d, *J* = 4.4 Hz, 1H, H1'''), 4.99 (dd, *J* = 7.3, 4.4 Hz, 1H, H2'''), 4.41 (q, *J* = 4.3 Hz, 1H, H4'), 4.35 (dd, *J* = 6.8, 4.3 Hz, 1H, H2''), 4.15 (q, *J* = 3.1 Hz, 1H, H4'''), 4.10 (q, *J* = 2.9 Hz, 1H, H4''), 3.85 – 3.65 (m, 10H, OMe DMT, H5''', H5''), 3.64 – 3.53 (m, 2H, H5'), 3.46 (AB, *J* = 10.6, 4.5 Hz, 1H, H5'), 2.13 (s, 3H, CH_3 Ac), 2.08 (s, 6H, CH_3 Ac), 2.05 (s, 3H, CH_3 Ac), 1.03 (s, 10H, CH_3, TBDPS), 0.99 (s, 9H, CH_3 TBDPS).

^{13}C NMR (101 MHz, $CDCl_3$) δ 170.59, 169.92, 169.72, 169.69 (CO Ac), 164.52 (CO Bz), 158.66, 158.64, 151.44, 149.62, 144.49 (Cq. arom.), 135.68, 135.65 (arom.), 135.58, 133.83, 133.03, 132.96, 132.85, 132.84 (Cq. arom.), 130.21, 130.14, 129.95, 129.92, 129.90, 128.96, 128.27, 127.99, 127.93, 127.90, 127.88, 127.85, 127.08 (arom.), 123.69 (Cq. arom.), 113.26 (arom.), 100.79 (C1''), 99.15 (C1'''), 88.41 (C1'), 86.75 (Cq. DMT), 83.19 (C4''), 82.72 (C4'''), 81.98 (C4'), 77.23 (C2'), 73.58 (C2''), 71.92 (C3'), 71.67 (C2''), 71.26 (C3''), 69.91 (C3'''), 63.79 (C5''), 63.29 (C5'''), 62.66 (C5'), 55.31 (OMe DMT), 26.86, 26.82 (CH_3 TBDPS), 21.08, 20.96, 20.82, 20.55 (CH_3 Ac), 19.29, 19.29 (Cq. TBDPS).

IR (film): 2935, 1743, 1739, 1507, 1245, 1241, 1236, 1233, 1227, 1223, 1178, 1175, 1113, 1107, 1092, 1037, 1030, 703 cm^{-1}.

HRMS (ESI$^+$) calcd for $C_{88}H_{96}N_5O_{19}Si_2$ (M+H) 1582.6311. Found 1582.6273. $[\alpha]_D^{20}$ +56.9 (c = 1, in $CHCl_3$)

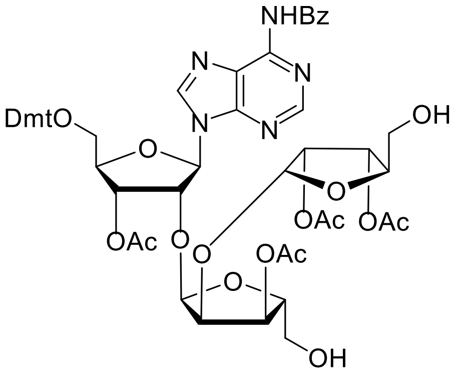

6-*N*-benzoyl-9-(3',3''2''',3'''-tetra-*O*-acetyl-5'-*O*-dimethoxyltrityl-β-parotriosyl)adenine (18)

Compound **17** (1.43 g, 0.90 mmol), dry THF (9 mL) and TBAF (tetrabutylammonium fluoride solution 1.0 M in THF, 2.7 mL, 2.7 mmol) was added into a flask and the mixture was stirred for 16 hours at room temperature. Excessive amount of EtOAc was added and the mixture was washed with H_2O (twice) and brine (once). The organic layer was dried with $MgSO_4$. The mixture was filtered, concentrated under reduced pressure and purified by silica gel column chromatography (DCM/methanol, 100/0 – 100/1 – 100/2 – 100/3) to obtain **18** as a white foam (0.86 g, 0.78 mmol, 87%).

^{1}H NMR (400 MHz, chloroform-*d*) δ 9.31 (s, 1H, NH), 8.61 (s, 1H, H2), 8.38 (s, 1H, H8), 8.08 – 7.99 (m, 2H, arom.), 7.63 – 7.55 (m, 1H, arom.), 7.55 – 7.47 (m, 2H, arom.), 7.45 – 7.40 (m, 2H, arom.), 7.36 – 7.28 (m, 4H, arom.), 7.28 – 7.18 (m, 3H, arom.), 6.79 (dd, *J* = 9.0, 3.0 Hz, 4H, DMT arom.), 6.24 (d, *J* = 5.6 Hz, 1H, H1'), 5.57 (dd, *J* = 5.4, 3.9 Hz, 1H, H3'), 5.45 (t, *J* = 5.5 Hz, 1H, H2'), 5.24 (dd, *J* = 7.3, 3.4 Hz, 1H, H3''), 5.15 (dd, *J* = 7.3, 4.0 Hz, 1H, H3'''), 5.11 (d, *J* = 4.3 Hz, 1H, H1''), 5.03 (d, *J* = 4.4 Hz, 1H, H1'''), 4.84 (dd, *J* = 7.3, 4.4 Hz, 1H, H2'''), 4.37 (d, *J* = 4.0 Hz, 1H, H4'), 4.13 – 4.10 (m, 2H, H2'', H4''), 4.05 (q, *J* = 3.2 Hz, 1H, H4'''), 3.81 – 3.61 (m, 10H, OMe DMT, H5'', H5'''), 3.56 (AB, *J* = 10.6, 3.9 Hz, 1H, H5'), 3.46 (AB, *J* = 10.6, 4.6 Hz, 1H, H5'), 3.07 (bs, 1H, OH), 2.53 (bs, 1H, OH), 2.14 (s, 3H, CH_3 Ac), 2.10 (s, 3H, CH_3 Ac), 2.08 (s, 3H, CH_3 Ac), 2.06 (s, 3H, CH_3 Ac).

^{13}C NMR (101 MHz, $CDCl_3$) δ 170.64, 170.16, 169.77, 169.73 (CO Ac), 165.07 (CO Bz), 158.64 (Cq. arom.), 152.75 (C2), 151.68, 149.74, 144.49, 135.65, 135.62, 133.55 (Cq. arom.), 132.95, 130.22, 130.19, 128.94, 128.28, 128.09, 127.97, 127.07 (arom.), 123.84 (Cq. arom), 113.24 (arom.), 101.14 (C1''), 98.77 (C1'''), 87.69 (C1'), 86.78 (Cq. arom.), 82.83 (C4''), 82.56 (C4'''), 82.39 (C4'), 76.93 (C2'), 73.04 (C2''), 72.19 (C3'), 71.36 (C2'''), 70.60 (C3''), 69.74 (C3'''), 62.97 (C5'), 62.24 (C5''), 61.80 (C5'''), 55.33 (OMe DMT), 21.03, 21.02, 20.77, 20.55 (CH_3 Ac).

IR (film): 2935, 1739, 1734, 1730, 1609, 1607, 1583, 1507, 1456, 1448, 1369, 1238, 1227, 1224, 1176, 1090, 1030, 829, 734, 705 cm^{-1}.

HRMS (ESI$^+$) calcd for $C_{56}H_{60}N_5O_{19}$ (M+H) 1106.3877. Found 1106.3896. $[\alpha]_D^{20}$ +68.0 (c = 1, in $CHCl_3$)

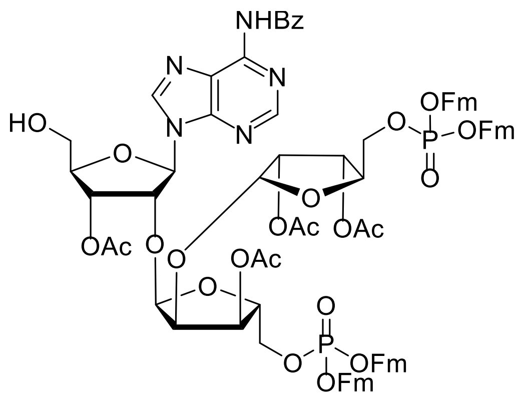

6-*N*-benzoyl-9-(3',3''2''',3'''-tetra-*O*-acetyl-5'',5'''-di-*O*-(di-fluorenyl)-β-parotriosyl)adenine (20)

Compound **18** (0.86 g, 0.78 mmol), DCI activator (0.25 M 4,5-dicyanoimidazole solution in ACN, 12.48 mL, 3.12 mmol) and freshly activated 3Å molecular sieves were added into flask. Compound **19** (0.2 M in ACN, 11.7 mL, 2.34 mmol) was added into the mixture and the reaction was stirred for 10 minutes at room temperature after which *tBu*OOH (5.5 M in decane, 1.42 mL, 7.80 mmol) was added at 0 ℃. The reaction was stirred at the same temperature for 45 minutes and quenched by saturated aqueous $NaHCO_3$. The mixture was filtered and an excessive amount of EtOAc was added to the filtration. The organic layer was washed by H_2O (once) and brine (twice) and was dried (Na_2SO_4). The mixture was filtered, concentrated under reduced pressure and co-evaporated with toluene (thrice). To the residue, DCM (10.4 mL) and TFA (0.15 mL, 1.95 mmol) were added and the reaction was stirred for 10 minutes at room temperature after which the reaction was quenched with saturated aqueous $NaHCO_3$. DCM extracted (twice) the mixture and the organic layers were combined and washed by H_2O (once) and brine (once). The organic layer was dried ($NaSO_4$), filtered and concentrated under reduced pressure. The residue was purified by silica gel column chromatography (DCM/methanol, 100/0 – 100/1 – 100/2 – 100/3) to obtain **20** as a white foam (1.01 g, 0.60 mmol, 77%).

^{1}H NMR (500 MHz, chloroform-*d*) δ 9.07 (s, 1H, NH), 8.78 (s, 1H, H2), 8.55 (s, 1H, H8), 7.95 – 7.89 (m, 2H, arom.), 7.72 – 7.61 (m, 8H, arom.), 7.56 – 7.51 (m, 1H, arom.), 7.48 – 7.14 (m, 26H), 6.24 (dd, *J* = 11.9, 2.4 Hz, 1H, OH), 6.12 (d, *J* = 8.0 Hz, 1H, H1'), 5.66 (d, *J* = 5.4 Hz, 1H, H3'), 5.15 (dd, *J* = 8.0, 5.4 Hz, 1H, H2'), 5.10 – 5.06 (m, 2H, H3'', H3'''), 4.94 (d, *J* = 4.4 Hz, 1H, H1''), 4.84 (dd, *J* = 7.7, 4.4 Hz, 1H, H2''), 4.70 (d, *J* = 4.3 Hz, 1H, H1'''), 4.31 – 3.80 (m, 22H, CH/CH2 Fm, H4', H2''', H4'', H4''', H5', H5'', H5'''), 2.19 (s, 3H, CH_3 Ac), 2.14 (s, 3H, CH_3 Ac), 2.08 (s, 3H, CH_3 Ac), 2.05 (s, 3H, CH_3 Ac).

^{13}C NMR (126 MHz, $CDCl_3$) δ 170.21, 169.82, 169.55, 169.46 (CO Ac), 164.41 (CO Bz), 152.31 (C2), 150.59, 150.36 (Cq. arom.), 143.11, 143.07, 143.00, 142.91, 141.43, 141.39, 133.50 (Cq. arom.), 132.87, 128.89, 127.95, 127.20, 127.17, 127.15, 127.13, 125.16, 125.13, 125.10 (arom.), 124.61 (Cq. arom.), 120.11, 120.08, 120.05 (arom.), 101.26 (C1'''), 98.15 (C1''), 89.28 (C1'), 86.88 (C4'), 79.97, 79.90 (C4'''), 79.50, 79.44 (C4''), 77.69 (C2'), 74.23 (C3'), 71.45 (C2'''), 71.08 (C2'), 69.85 (C3'''), 69.48, 69.43, 69.37 (CH_2 Fm), 69.06 (C3''), 66.32, 66.29 (C5''), 66.09, 66.05 (C5'''), 62.95 (C5'), 47.95, 47.93, 47.89, 47.87, 47.83 (CH Fm), 21.15, 20.81, 20.69, 20.65 (CH_3 Ac).

^{31}P NMR (202 MHz, $CDCl_3$) δ -1.15, -1.20.

IR (film): 2931, 1743, 1739, 1582, 1451, 1448, 1238, 1234, 1103, 1067, 1016, 991, 759, 740 cm^{-1}.

HRMS (ESI$^+$) calcd for $C_{91}H_{84}N_5O_{23}P_2$ (M+H) 1676.5027. Found 1676.5161. $[\alpha]_D^{20}$ +38.3 (c = 1, in $CHCl_3$)

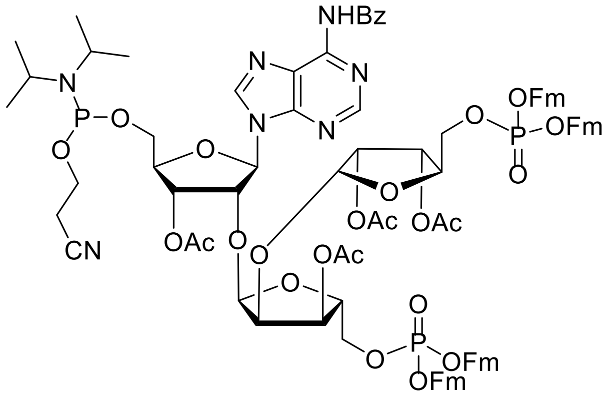

6-*N*-benzoyl-9-(3',3''2''',3'''-tetra-*O*-acetyl-5'-*O*-(*N*,*N*-diisopropylamino-*O*-cyanoethyl)phosphoramidite)-5'',5'''-di-*O*-(di-flourenyl)-β-parotriosyl)adenine (3) Compound **20** (1.01 g, 0.60 mmol), DMF (6 mL), DIPEA (0.21 mL, 1.2 mmol) and 2-cyanoethyl *N*,*N*-diisopropylchlorophosphoramidite **21** (0.15 mL, 0.66 mmol) were added into the flask under N_2. The reaction was stirred at room temperature for 10 minutes after which it was quenched with 0.3 mL methanol. An excessive amount of EtOAc was added and the mixture was washed with saturated aqueous $NaHCO_3$ (twice), H_2O (once) and brine (once). The organic layer was dried (Na_2SO_4) and filtered. The filtration was co-evaporated with toluene (once) then purified by automatic column (DCM/acetone, 100/0 – 90/10 – 80/20) to furnish **3** as a white foam (684 mg, 0.36 mmol, 60%). **Note**: Careful washes were needed for the work-up because the DIPEA in the reaction could cleave the Fm group. Column chromatography was performed by automation using a Biotage® Isolera™ Spektra Four machine using High-quality IRR silica gel column (40-63 μm).

^{1}H NMR (500 MHz, chloroform-*d*) δ 9.39 – 9.15 (m, 1H, NH), 8.72 (d, *J* = 7.4 Hz, 1H, H2), 8.50 (d, *J* = 10.7 Hz, 1H, H8), 8.01 (d, *J* = 7.7 Hz, 2H, arom.), 7.68 – 7.13 (m, 35H), 6.24 (dd, *J* = 9.8, 4.8 Hz, 1H, H1'), 5.49 (dt, *J* = 15.0, 5.0 Hz, 1H, H3'), 5.20 (ddt, *J* = 13.5, 7.6, 3.9 Hz, 3H, H1'', H2', H3''), 5.14 – 5.05 (m, 2H, H1''', H3'''), 4.79 (td, *J* = 7.8, 4.4 Hz, 1H, H2'''), 4.40 (q, *J* = 4.1 Hz, 1H, H4'), 4.41 – 3.75 (m, 23H, CH_2/CH Fm, H4'', H4''', H2'', H5', H5'', H5''', O$CH_2$$CH_2$CN), 3.59 (dddd, *J* = 13.7, 9.5, 6.8, 2.5 Hz, 2H, $(CH_3)_2$*CH*), 2.72 (td, *J* = 6.2, 2.7 Hz, 1H, C*H*HCN), 2.66 (t, *J* = 6.3 Hz, 1H, C*H*HCN), 2.59 (dq, *J* = 6.2, 3.7, 3.0 Hz, 1H, C*H*HCN), 2.15 – 2.06 (m, 12H, CH_3 Ac), 1.22 – 1.10 (m, 12H, *$(CH_3)_2$*CHN).

^{13}C NMR (126 MHz, $CDCl_3$) δ 170.16, 170.15, 169.54, 169.52, 169.51, 169.47, 169.27, 169.23 (CO Ac), 164.62, 164.59 (CO Bz), 151.53, 151.43, 149.67, 142.99, 142.98, 142.95, 142.88, 142.87, 142.81, 141.27, 141.25, 141.24, 133.60, 132.58, 128.68, 128.66, 127.93, 127.84, 127.79, 127.04, 125.04, 125.02, 124.99, 124.94 (arom.), 123.82, 123.77 (Cq. arom.), 119.98, 119.97, 119.92, 119.88 (arom.), 117.86, 117.74 (CN), 100.70 (C1''), 98.80 (C1'''), 87.77, 87.48 (C1'), 82.25, 82.18, 82.07, 82.00 (C4'), 80.41, 80.35 (C4''), 80.30, 80.23 (C4'''), 77.64, 77.59

(C2'), 72.67 (C2''), 71.62, 71.52 (C3'), 70.81, 70.76 (C2'''), 69.84, 69.81 (C3''), 69.29, 69.24 (CH_2 Fm), 69.11 (C3'''), 66.41, 66.40, 66.37 (C5''), 66.19, 66.15 (C5'''), 62.13, 62.00, 61.87, 61.75 (C5'), 58.67, 58.62, 58.51, 58.45, 58.13, 58.08 (O$CH_2$$CH_2$CN), 47.81, 47.74 (CH Fm), 43.15, 43.12, 43.06, 43.02 ($(CH_3)_2$*CH*N), 24.65, 24.62, 24.59, 24.57, 24.54, 24.52 (*$(CH_3)_2$*CHN), 20.82, 20.76, 20.72, 20.49 (CH_3 Ac), 20.35, 20.30 (*CH_2*CN), 20.27 (CH_3 Ac).
^{31}P NMR (202 MHz, $CDCl_3$) δ 149.11, 148.96, 14.18 (H-phosphonate), -1.60, -1.62, -1.70, -1.73.
IR (film): 2969, 1743, 1698, 1609, 1581, 1511, 1451, 1367, 1238, 1158, 1017, 984, 759, 742 cm^{-1}.
HRMS (ESI$^+$) calcd for $C_{94}H_{87}N_6O_{25}P_3$ ([H-phosphonate]+H) 1793.5007. Found 1793.5032.
$[\alpha]_D^{20}$ +34.8 (c = 1, in DCM)

PAR branch point (1) and **1-*O*-methyl-α-mono ADPr (23)**

200 mg resin **2** was added into a 5 mL reaction syringe with filter frit and the resin was washed with ACN (five times) under N_2. 3 mL DBU solution (10%, v/v, in ACN) was added into the syringe and the reaction was shaken for 20 minutes to remove the 5-phosphate Fm groups after which the solution was drained. The DBU treatment was repeated for another 20 minutes. The resin was washed with ACN (five times) and dried under reduced pressure to remove traceless water before use. Part of the obtained resin (50 mg, 10 μmol) was transferred into a reaction column of a Mermade 6 oligonucleotide synthesizer (BioAutomation) and the complete synthesis was performed under an argon atmosphere. Cycle A and B were performed once.

Cycle A:

The resin was rinsed with ACN (thrice) and drained. 5-(Benzylthio)-1*H*-tetrazole (BTT) (480 uL, 0.25 M in ACN) and **3** (400 uL, 0.1 M in ACN) were added into the resin and the mixture was left to stand for 10 minutes, drained. This coupling was repeated twice more. The resin was rinsed with ACN (thrice). The intermediate phosphate-phosphite was oxidized with (1S)-(+)-(10-camphorsulfonyl)-oxaziridine (CSO) solution (2 mL, 0.5 M in ACN) for 5 minutes (2 x). The resin was drained and washed with ACN (thrice). DBU solution (2 mL, 10%, v/v, in ACN) was added into the resin and was left to stand for 10 minutes (four times) to remove 5-phosphate Fm groups and the CE group after which the resin was drained and washed with ACN (thrice).

Cycle B:

The resin was rinsed with ACN (thrice) and drained. BTT (480 uL, 0.25 M in ACN) and **4** (400 uL, 0.1M in ACN) were added into the resin and the mixture was left to stand for 10 minutes, drained. This coupling was repeated thrice. The resin was rinsed by with ACN (thrice). The intermediate phosphate-phosphite was oxidized with (1S)-(+)-(10-camphorsulfonyl)-

oxaziridine (CSO) solution (2 mL, 0.5 M in ACN) for 5 minutes (twice). The resin was drained and washed with ACN (thrice). DBU solution (2 mL, 10%, v/v, in ACN) was added into the resin and was left to stand for 10 minutes to remove the CE group after which the resin was drained and washed with ACN (thrice).

After cycle A and B, the resin was transferred to a tube and treated with 10 mL NH_4OH (35%). The mixture was stirred overnight in a sealed flask, filtered and concentrated under reduced pressure. The crude product was purified by anion exchange to obtain branched core oligomer **1** 0.68 mg (0.43 mmol, 4%) and meADPr **23** 2.83 mg (4.94 mmol, 50%) as white solids.

Column: Resource Q 6mL.

Gradient: 25% - 75%. (A: 10 mM NH_4OAc, B: 1 M NH_4OAc)

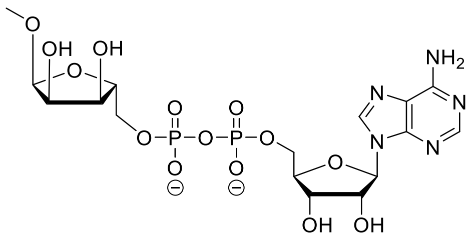

1-*O*-methyl-α-ADP-riboside (23)

^{1}H NMR (850 MHz, Deuterium Oxide) δ 8.53 (s, 1H, H2), 8.28 (s, 1H, H8), 6.15 (d, *J* = 5.9 Hz, 1H, H1'), 4.54 (d, *J* = 4.5 Hz, 2H, H3'), 4.40 (d, *J* = 2.9 Hz, 1H, H4'), 4.23 (t, *J* = 4.0 Hz, 2H, H5'), 4.18 (s, 1H, H4''), 4.13 – 4.12 (m, 2H, H2'', H3''), 4.01 (t, *J* = 4.7 Hz, 2H, H5''), 3.38 (s, 3H, OMe).

^{13}C NMR (214 MHz, D_2O) δ 155.72 (C4), 152.95 (C8), 149.21 (C6), 118.70 (C5), 103.26 (C1''), 86.83 (C1'), 83.99, 83.95 (C4'), 83.23, 83.18 (C4''), 74.27 (C2'), 70.83 (C2''), 70.43 (C3'), 69.68 (C3''), 65.62, 65.60 (C5''), 65.22, 65.20 (C5'), 55.46 (OMe)

^{31}P NMR (202 MHz, D_2O) δ -10.47, -10.57, -10.68, -10.78.

LC-MS: Rt = 3.57 min. 0-50% NH_4OAc. ESI MS+ calc. 574.1 found 574.1 $[M+1]^+$. HRMS (ESI^+) calcd for $C_{16}H_{26}N_5O_{14}P_2$ (M+H) 574.0946. Found 574.0949.

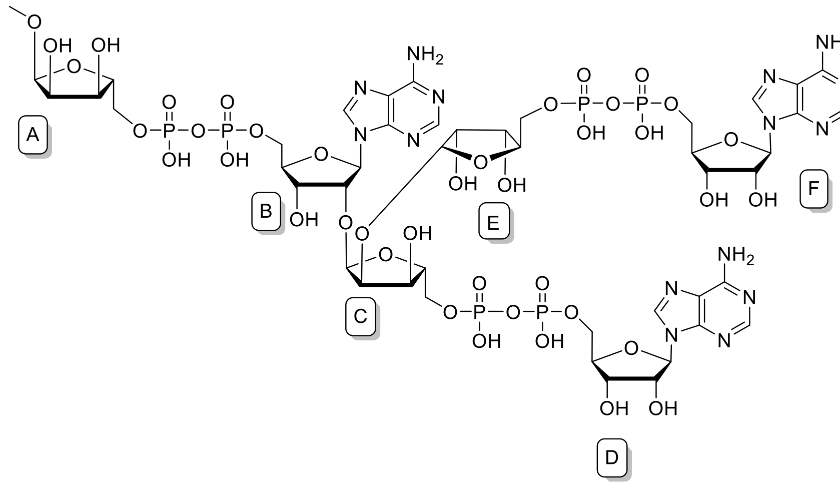

1-*O*-Methyl-α-branched tri-ADP riboside (1)

^{1}H NMR (850 MHz, Deuterium Oxide) δ 8.35 (s, 2H, H2), 8.20 (s, 1H, H2), 8.06 – 8.05 (m, 2H, H8), 7.99 (s, 1H, H8), 6.01 (d, J = 4.6 Hz, 1H, H1-B), 5.92 (d, J = 11.4 Hz, 2H, H1-DF), 5.13 – 5.06 (m, 1H, H1-C), 4.94 (d, J = 4.3 Hz, 1H, H1-E), 4.83 (t, J = 2.3 Hz, 1H, H1-A), 4.60 (t, J = 5.3 Hz, 1H, H2-D), 4.58 – 4.56 (m, 2H, H2-BF), 4.46 (t, J = 5.0 Hz, 1H, H3-B), 4.42 (t, J = 4.5 Hz, 1H, H3-F), 4.39 (s, 1H, H3-D), 4.30 – 3.93 (m, 24H, the rest H2, H3, H5), 3.30 (s, 3H, OMe).

^{13}C NMR (214 MHz, D_2O) δ 119.06, 119.05 (C5), 104.15 (C1-A), 101.98 (C1-E), 101.08 (C1-C), 88.07 (C1-D), 87.91 (C1-F), 87.25 (C1-B), 85.04, 84.76, 84.69, 84.67, 84.09, 84.06 (C4-ABDCEF), 80.02, 80.00, 75.61, 75.44, 75.34, 72.44, 71.72, 71.30, 71.14, 70.56, 70.53, 70.48, 70.26 (C2, C3-ABCDEF), 66.52, 66.39, 66.33, 66.18, 66.09, 65.67 (C5-ABDCEF), 56.36 (OMe).

^{31}P NMR (202 MHz, D_2O) δ -11.04, -11.11, -11.14, -11.17, -11.19, -11.24, -11.33, -11.43.

LC-MS: Rt = 3.54 min. 0-50% NH_4OAc. ESI MS+ calc. 1656.2 found 1656.3 $[M+1]^+$. HRMS (ESI^+) calcd for $C_{46}H_{68}N_{15}O_{40}P_6$ (M+H) 1656.2168. Found 1656.2171.

Part 4. LC-MS analysis and HRMS analysis of compound 1 and 23

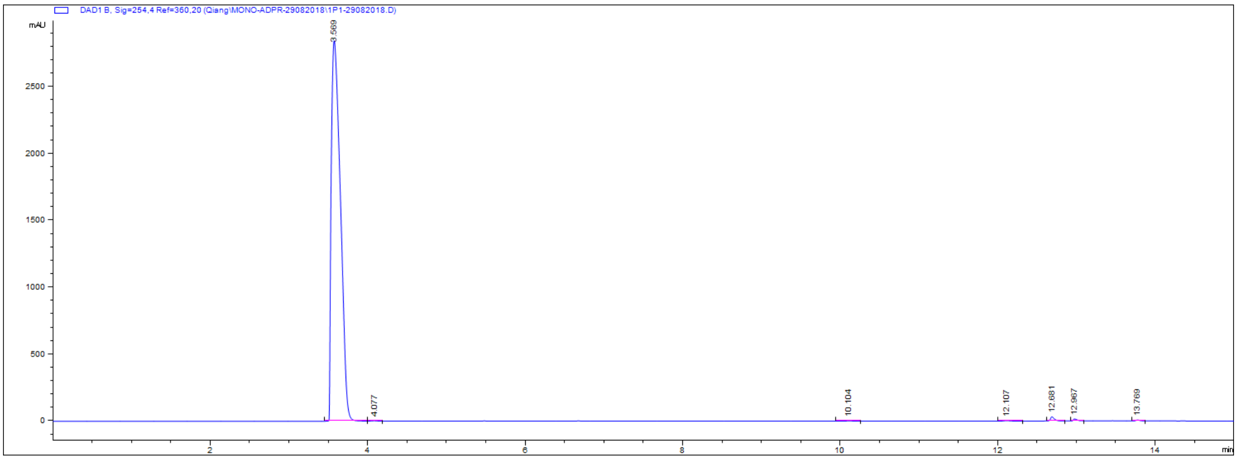

Supplementary Figure 17. UV trace of LC-MS analysis of compound **23**

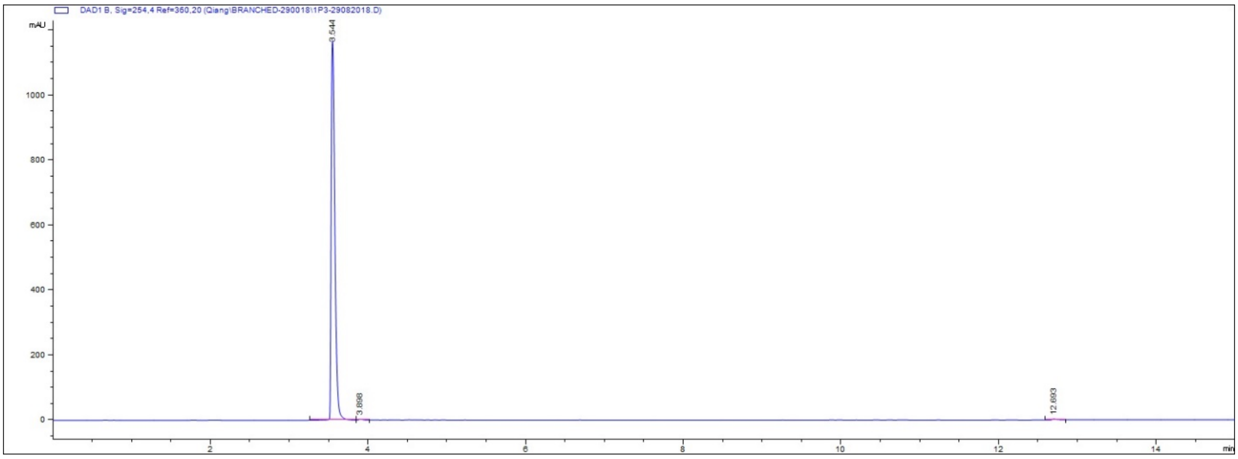

Supplementary Figure 18. UV trace of LC-MS analysis of compound **1**

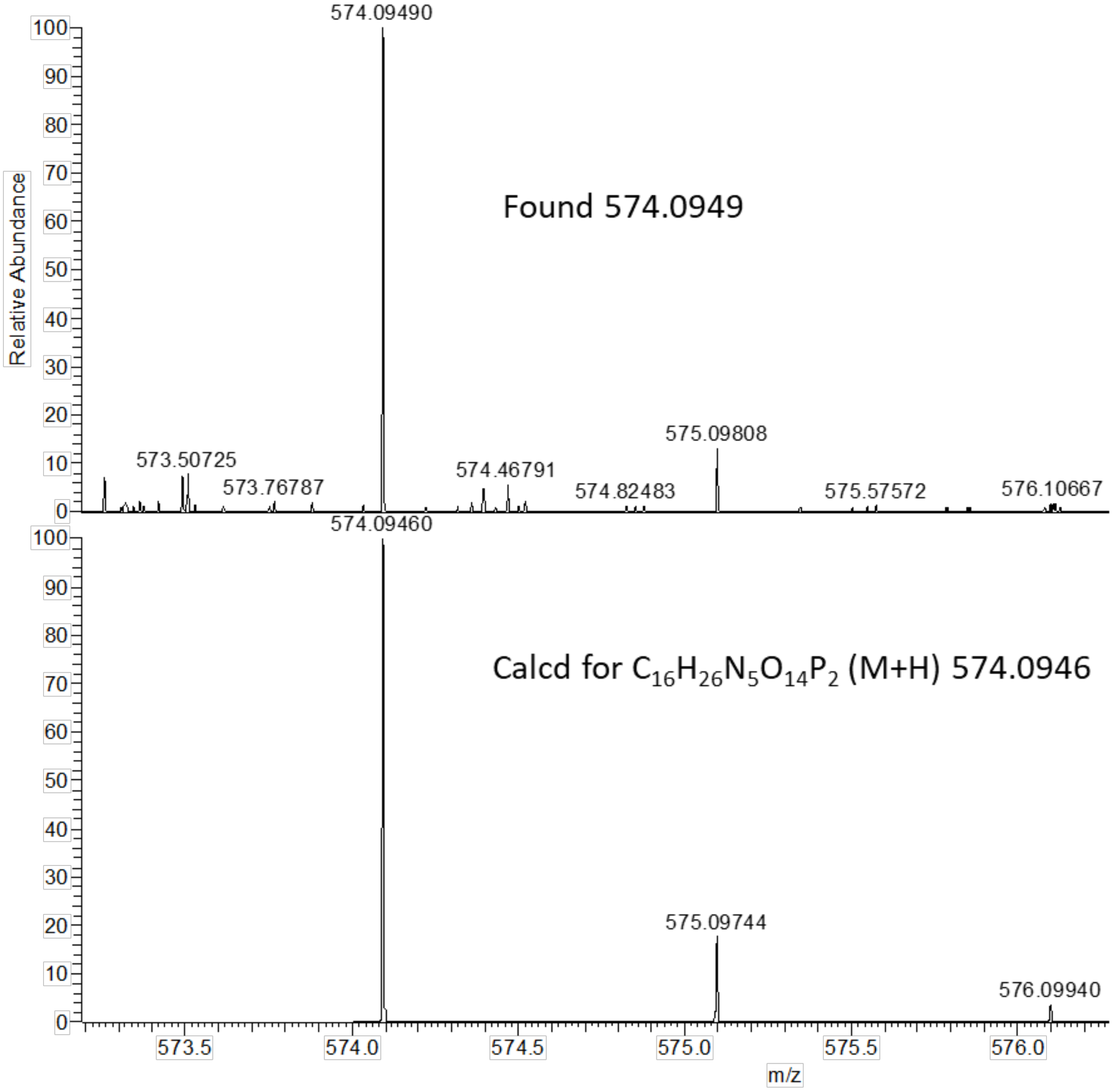

Supplementary Figure 19. HRMS analysis of compound **23**

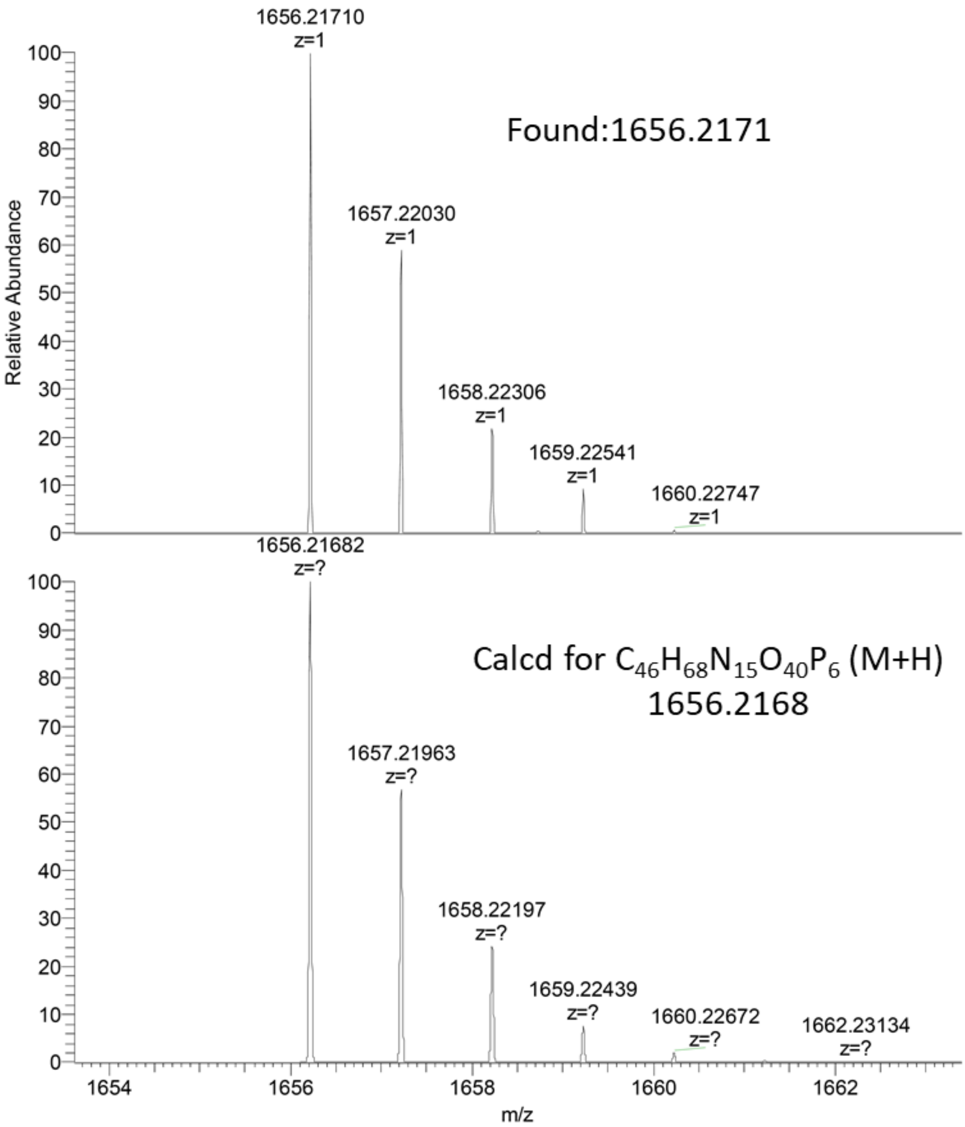

Supplementary Figure 20. HRMS analysis of compound **1**

NMR spectra data of all compounds

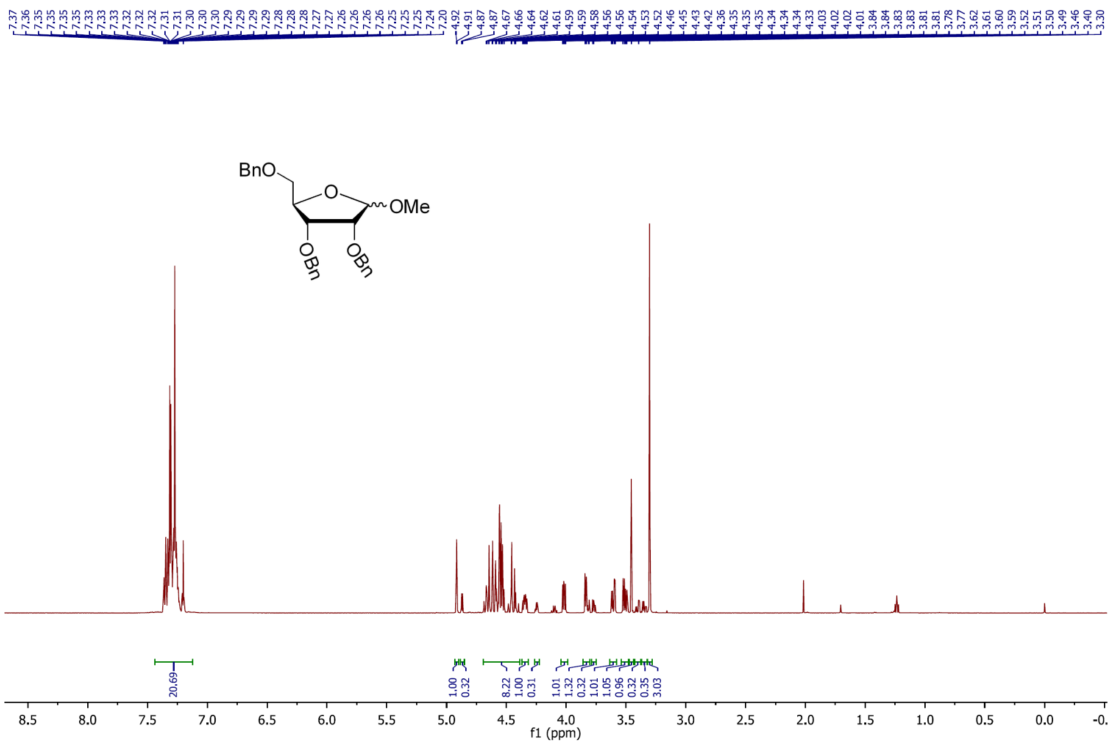

Supplementary figure 21: ^{1}H-NMR spectrum of compound **S1**.

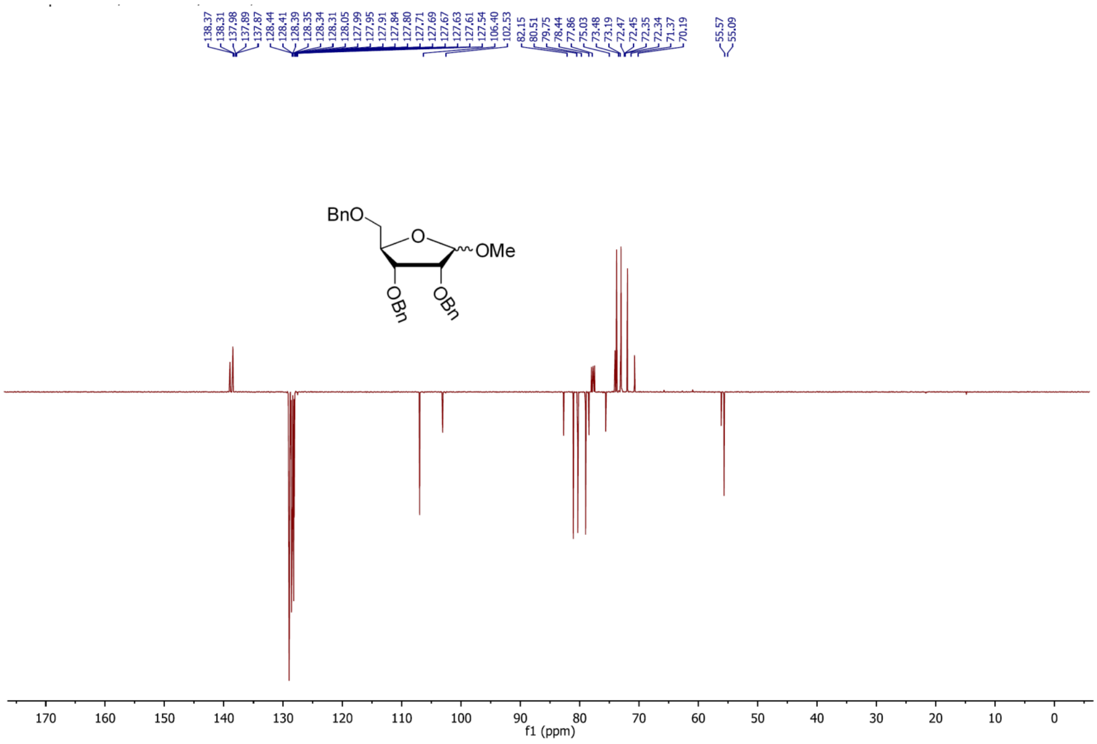

Supplementary figure 22: ^{13}C-NMR spectrum of compound **S1**.

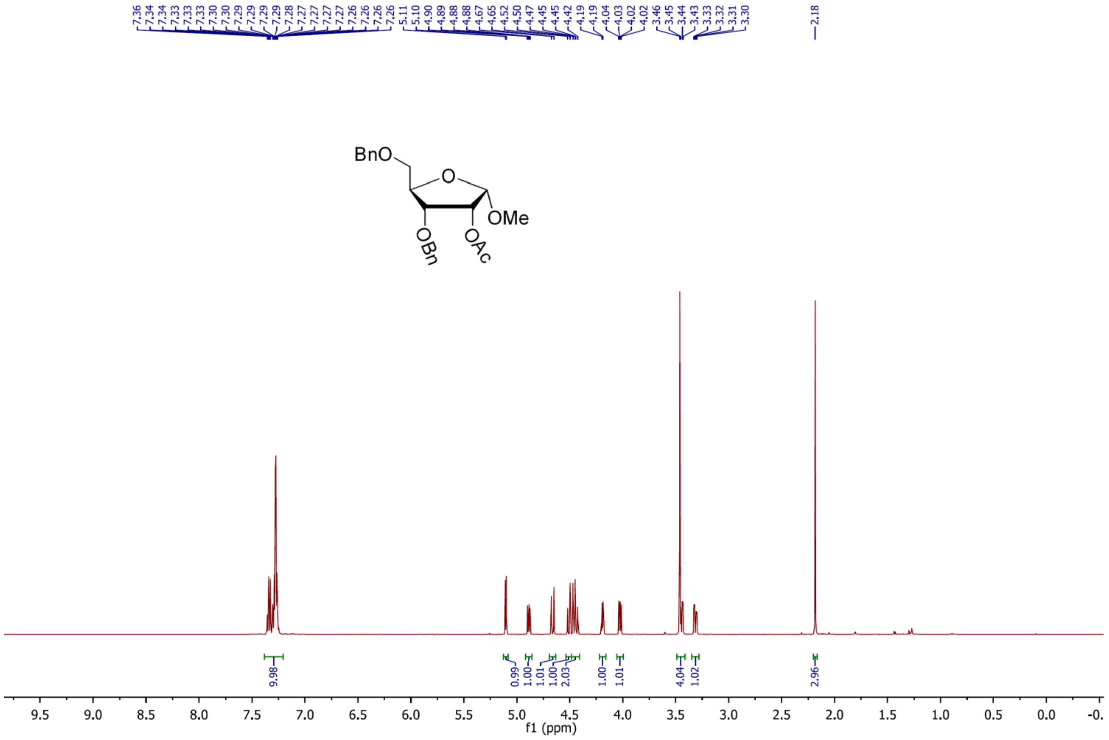

Supplementary figure 23: ^{1}H-NMR spectrum of compound **S2**.

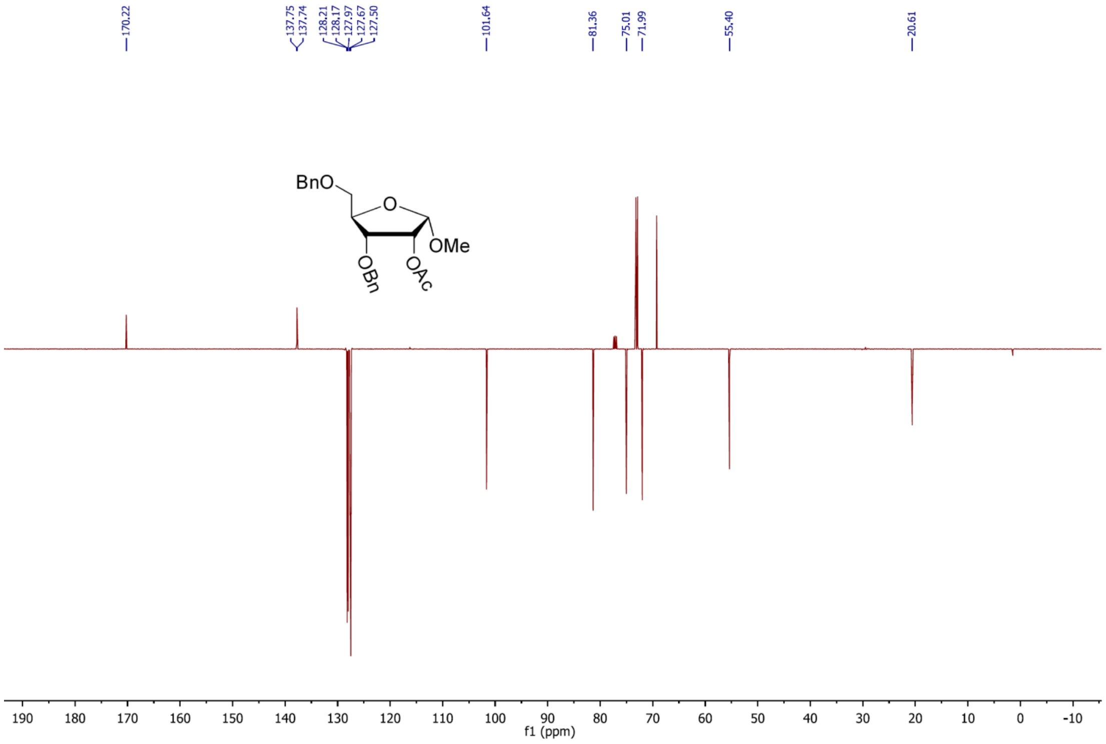

Supplementary figure 24: ^{13}C-NMR spectrum of compound **S2**.

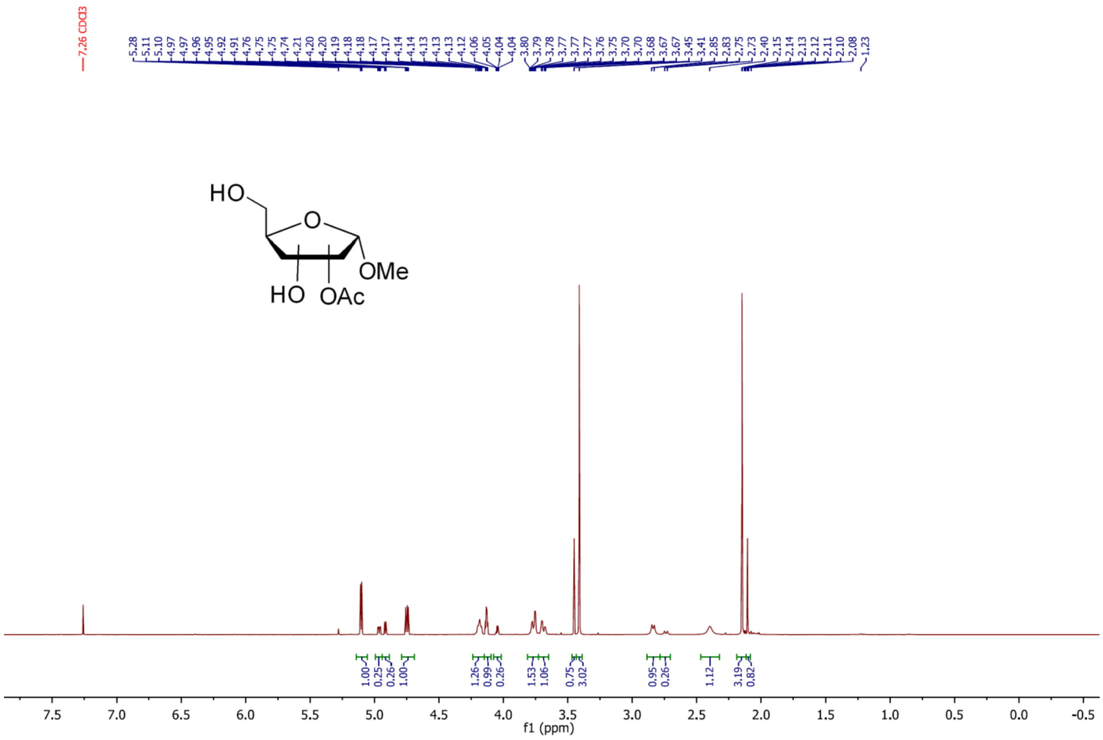

Supplementary figure 25: ^{1}H-NMR spectrum of compound **S3**.

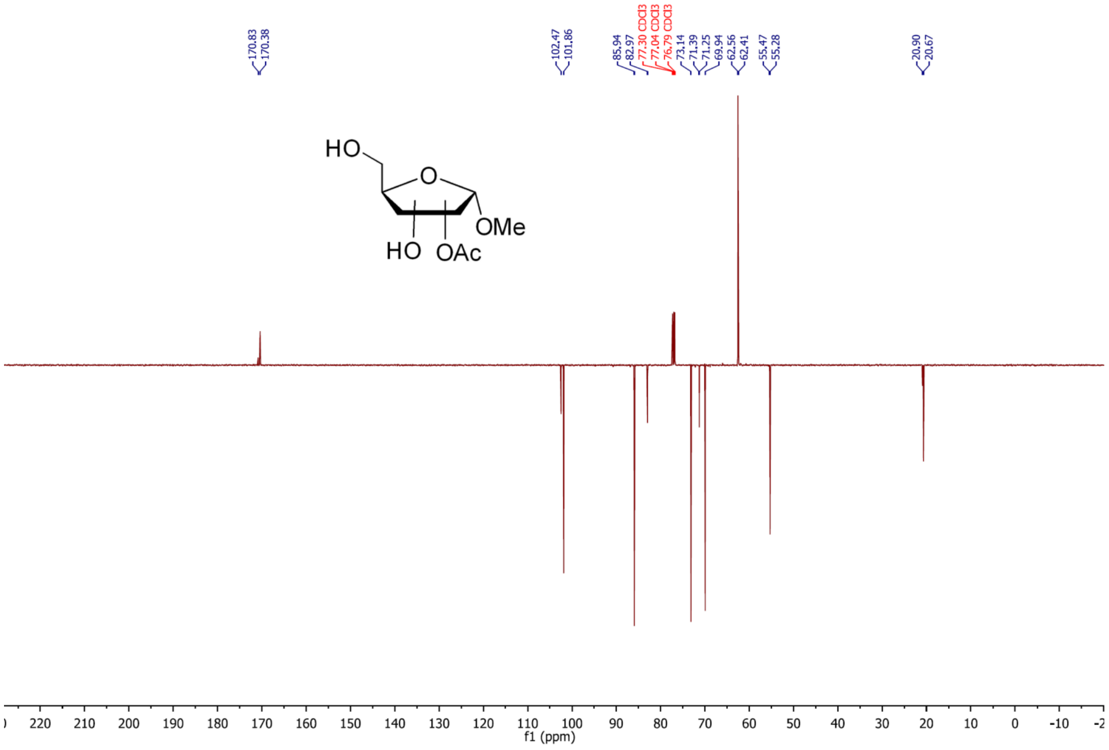

Supplementary figure 26: ^{13}C-NMR spectrum of compound **S3**.

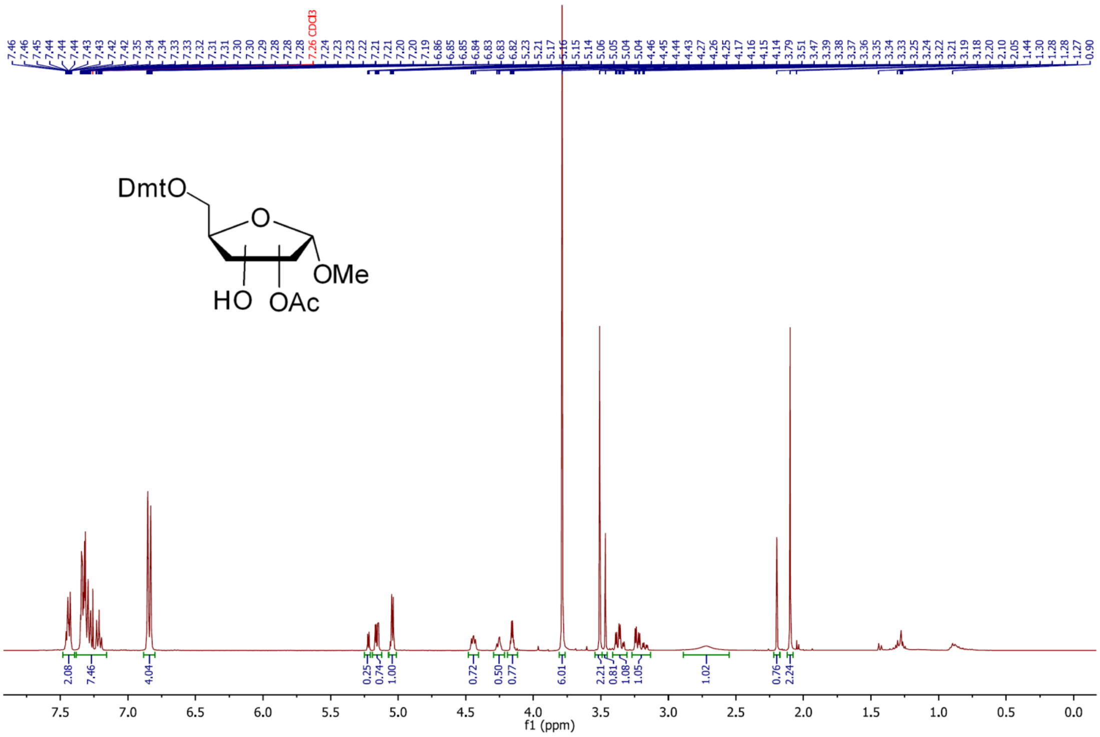

Supplementary figure 27: ^{1}H-NMR spectrum of compound **S4**.

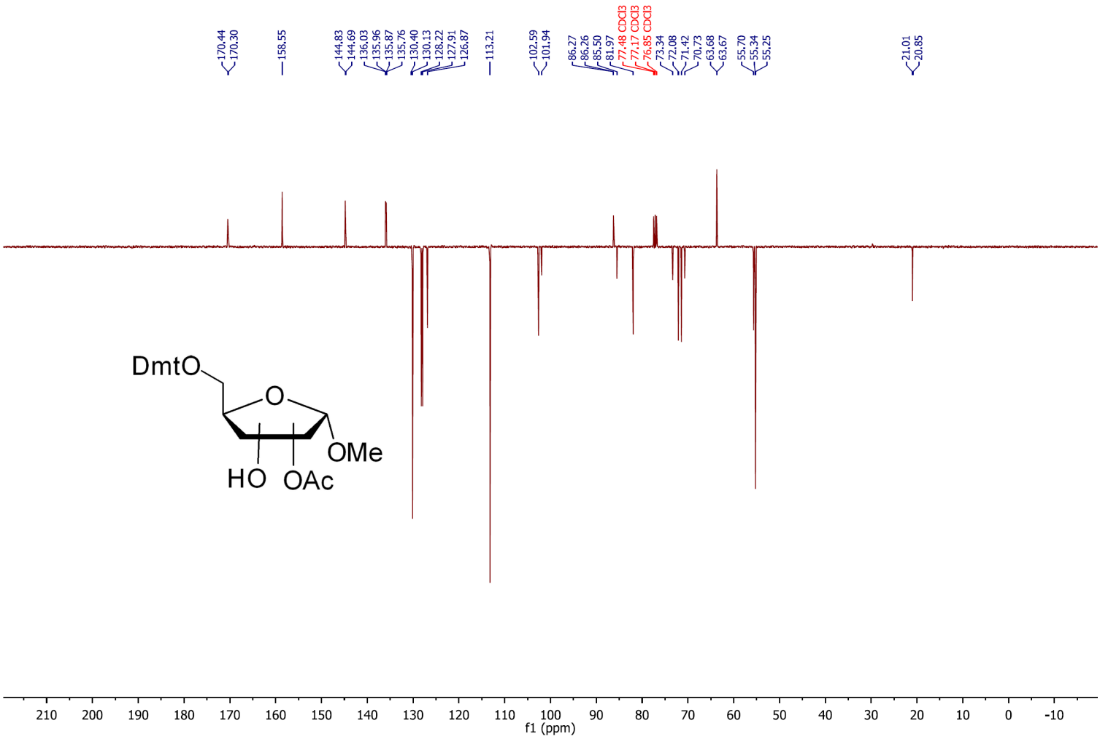

Supplementary figure 28: ^{13}C-NMR spectrum of compound **S4**.

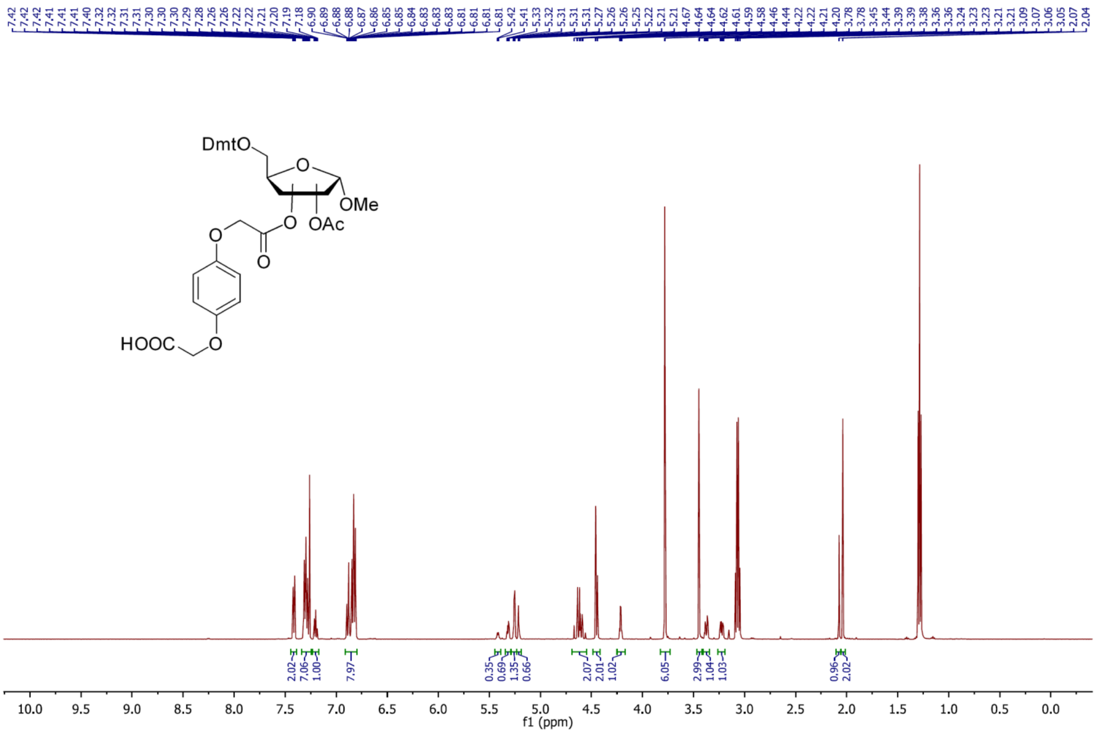

Supplementary figure 29: ^{1}H-NMR spectrum of compound **S5**.

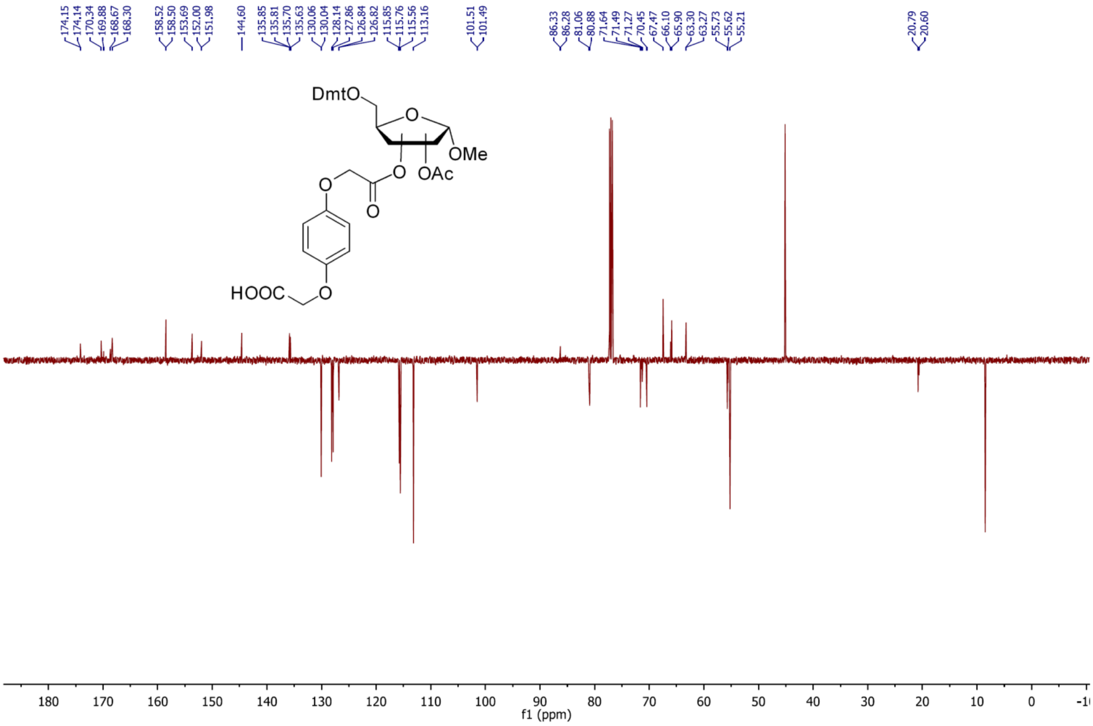

Supplementary figure 30: ^{13}C-NMR spectrum of compound **S5**.

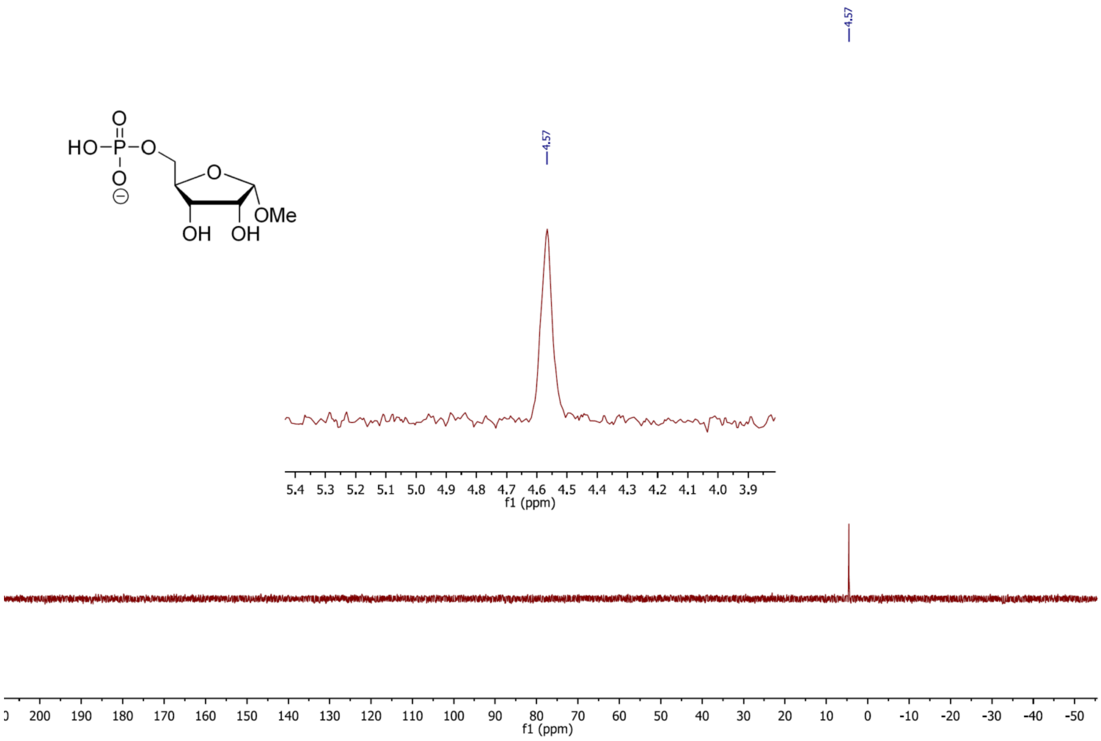

Supplementary figure 31: ^{31}P-NMR spectrum of compound **2** (deprotected).

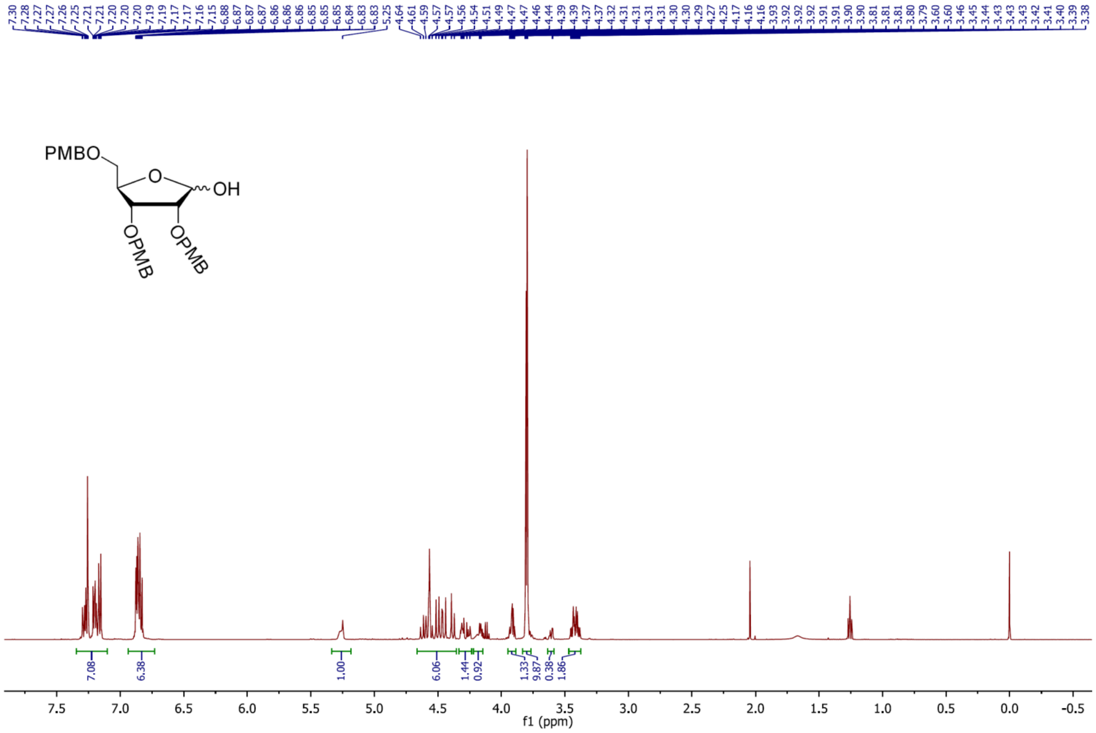

Supplementary figure 32: ^{1}H-NMR spectrum of compound **5**.

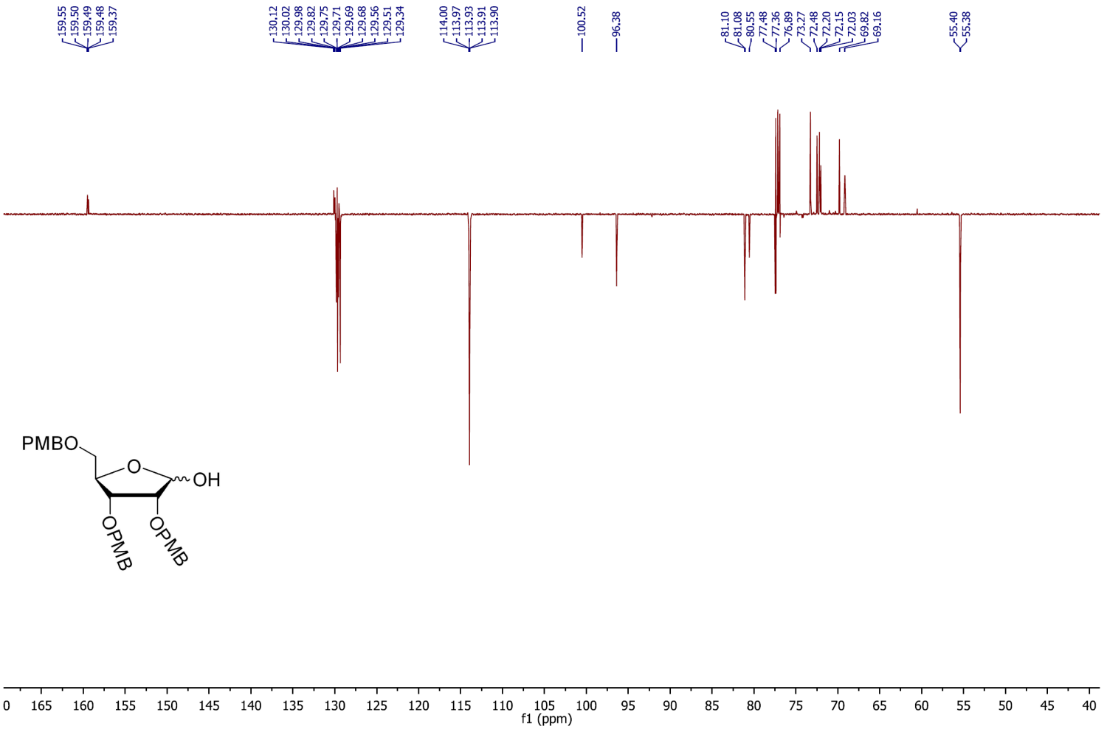

Supplementary figure 33: ^{13}C-NMR spectrum of compound **5**.

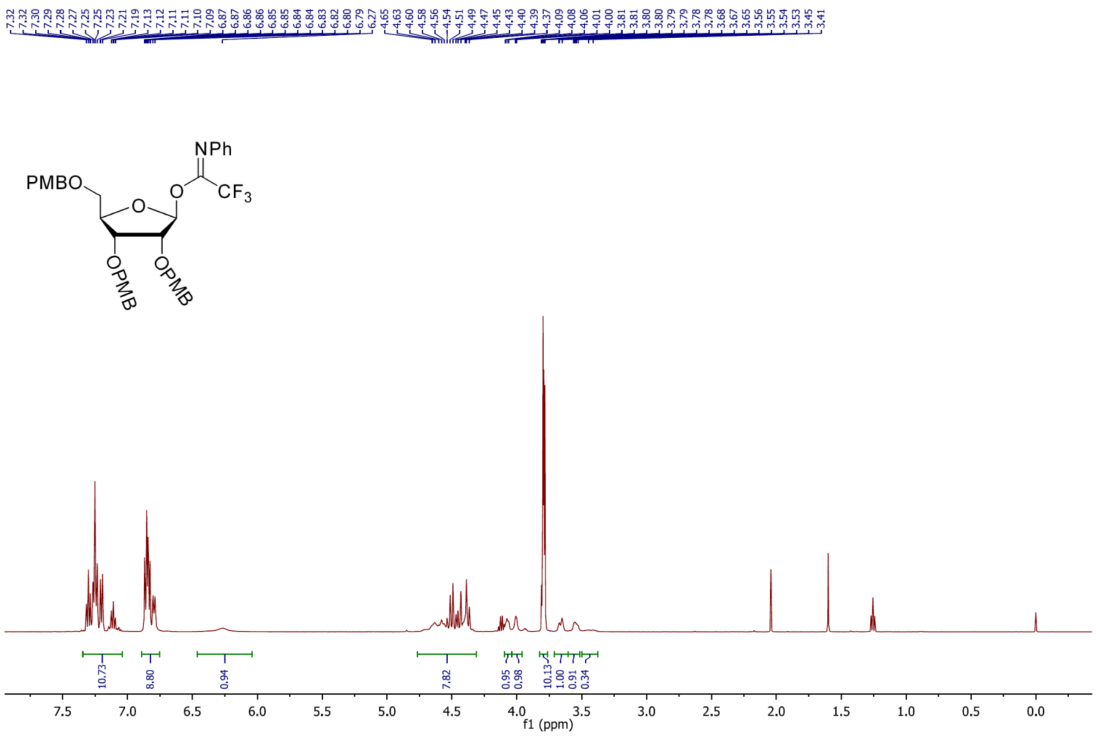

Supplementary figure 34: ^{1}H-NMR spectrum of compound **6**.

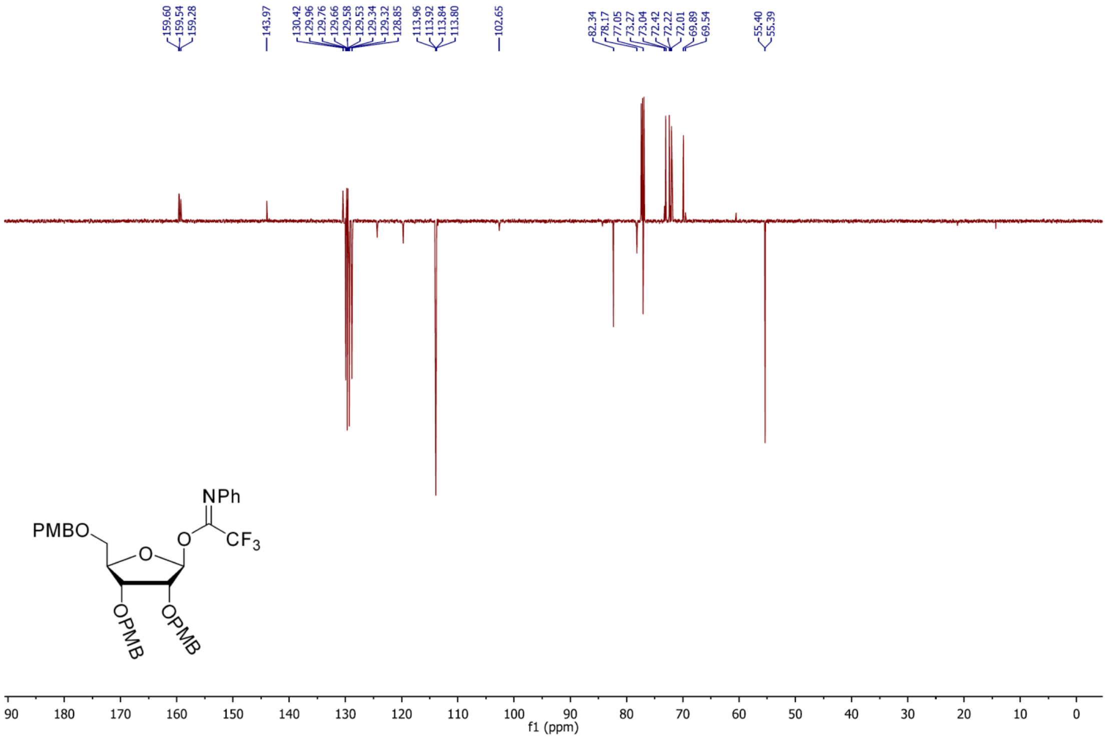

Supplementary figure 35: ^{13}C-NMR spectrum of compound **6**.

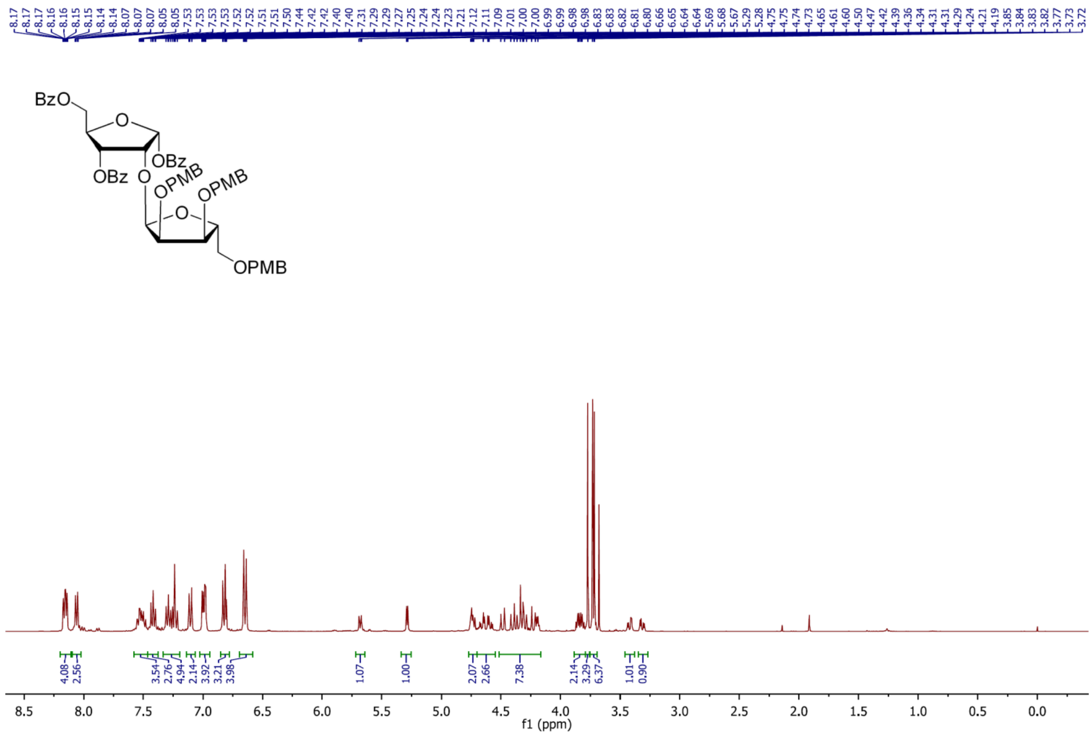

Supplementary figure 36: ^{1}H-NMR spectrum of compound **8**.

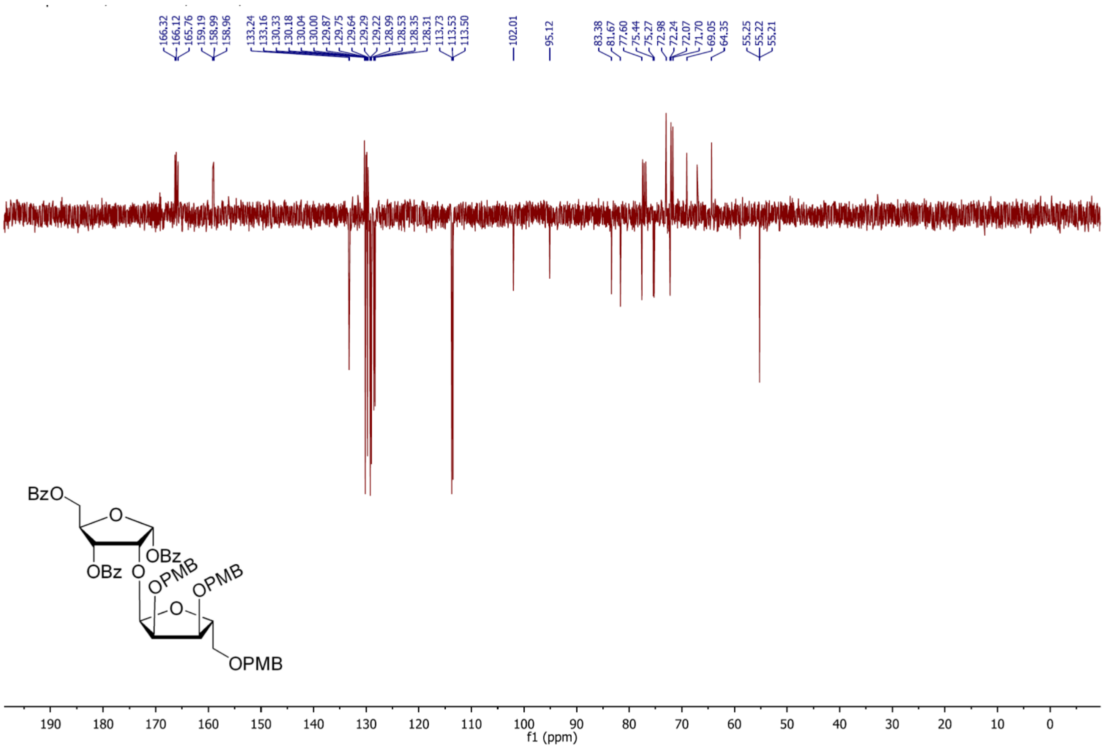

Supplementary figure 37: ^{13}C-NMR spectrum of compound **8**.

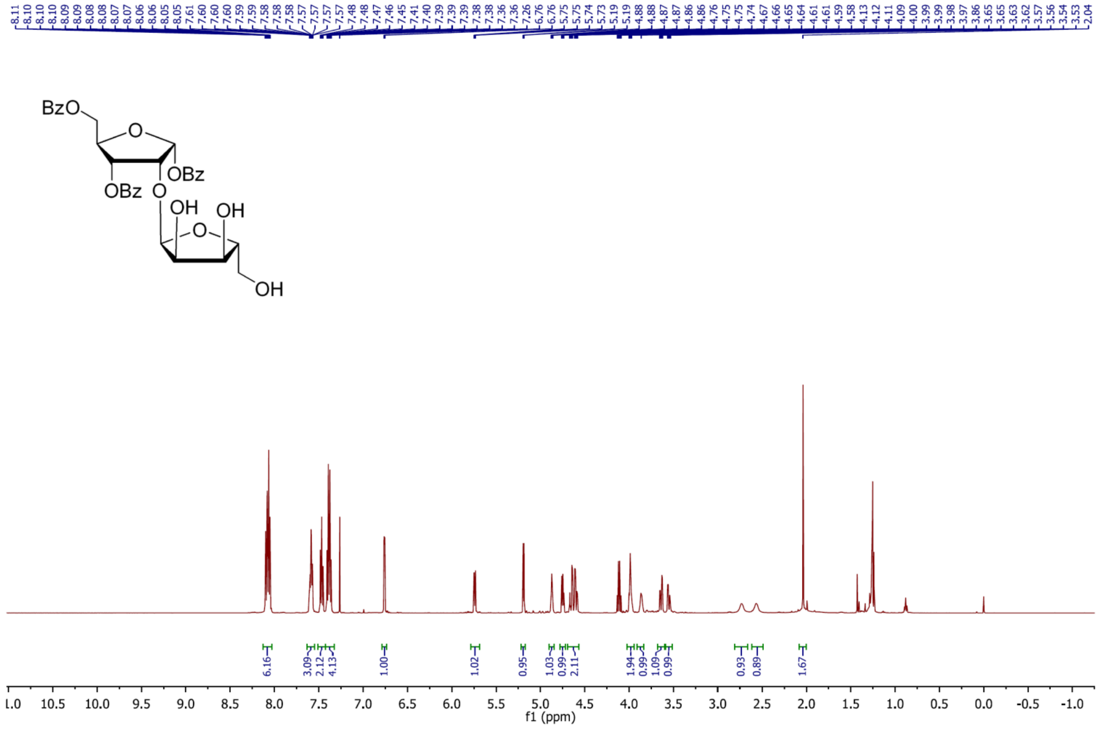

Supplementary figure 38: ^{1}H-NMR spectrum of compound **9**.

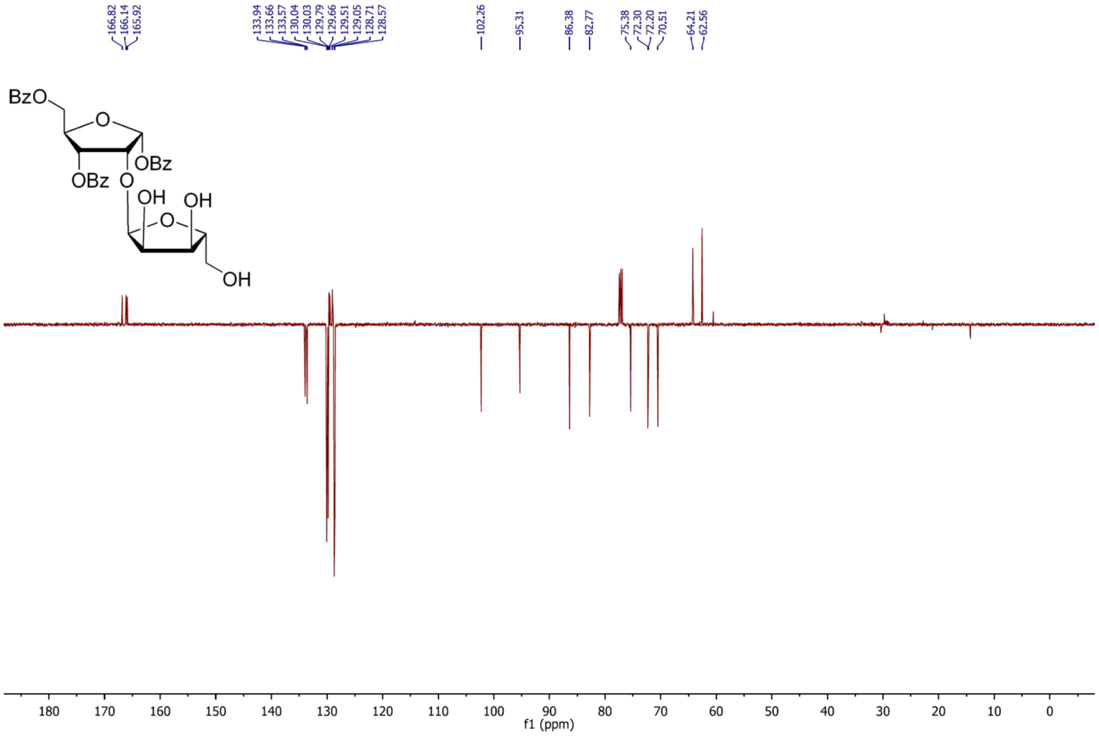

Supplementary figure 39: ^{13}C-NMR spectrum of compound **9**.

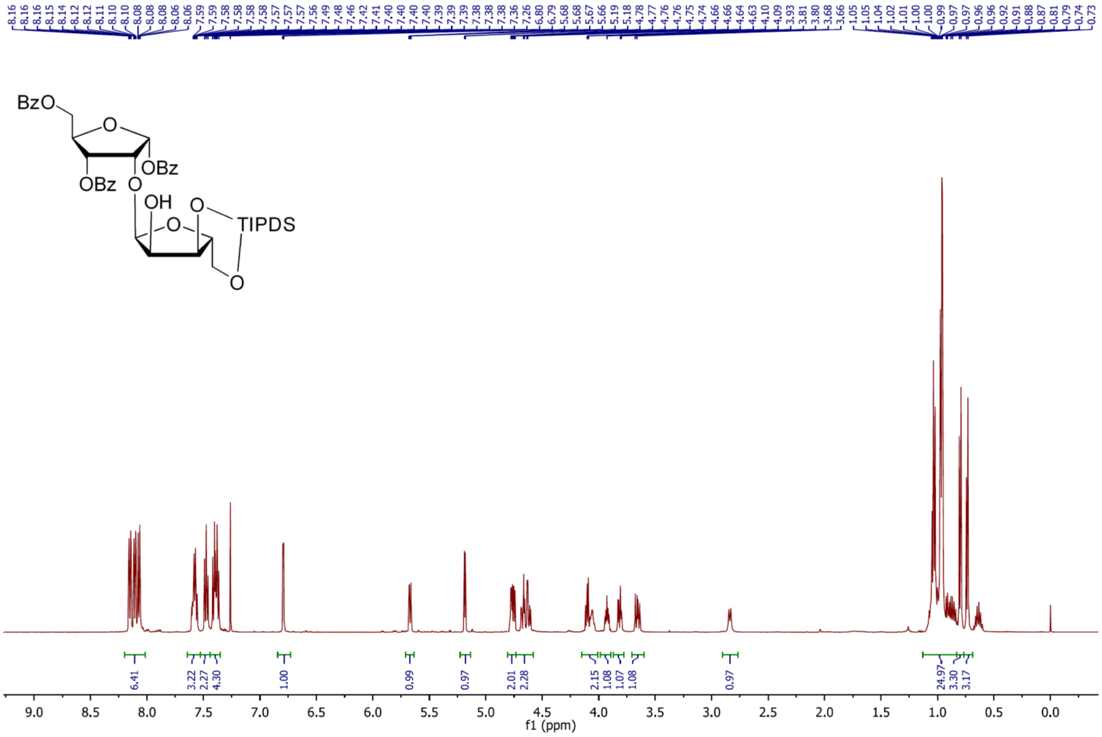

Supplementary figure 40: ^{1}H-NMR spectrum of compound **10**.

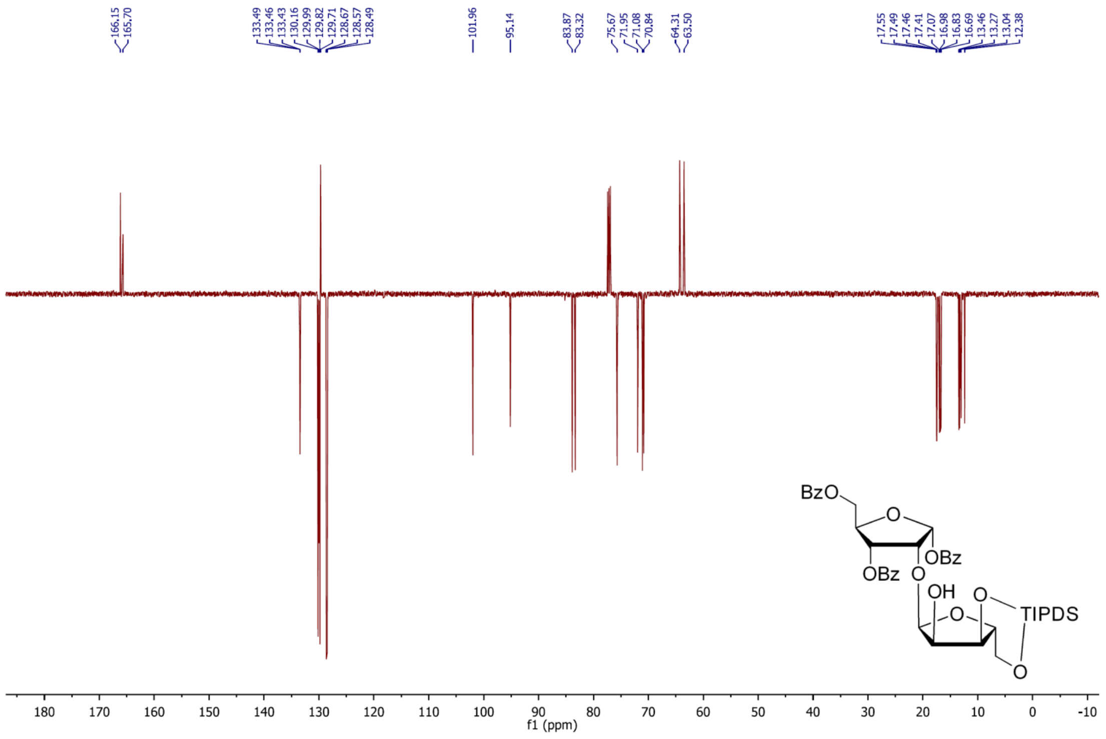

Supplementary figure 41: ^{13}C-NMR spectrum of compound **10**.

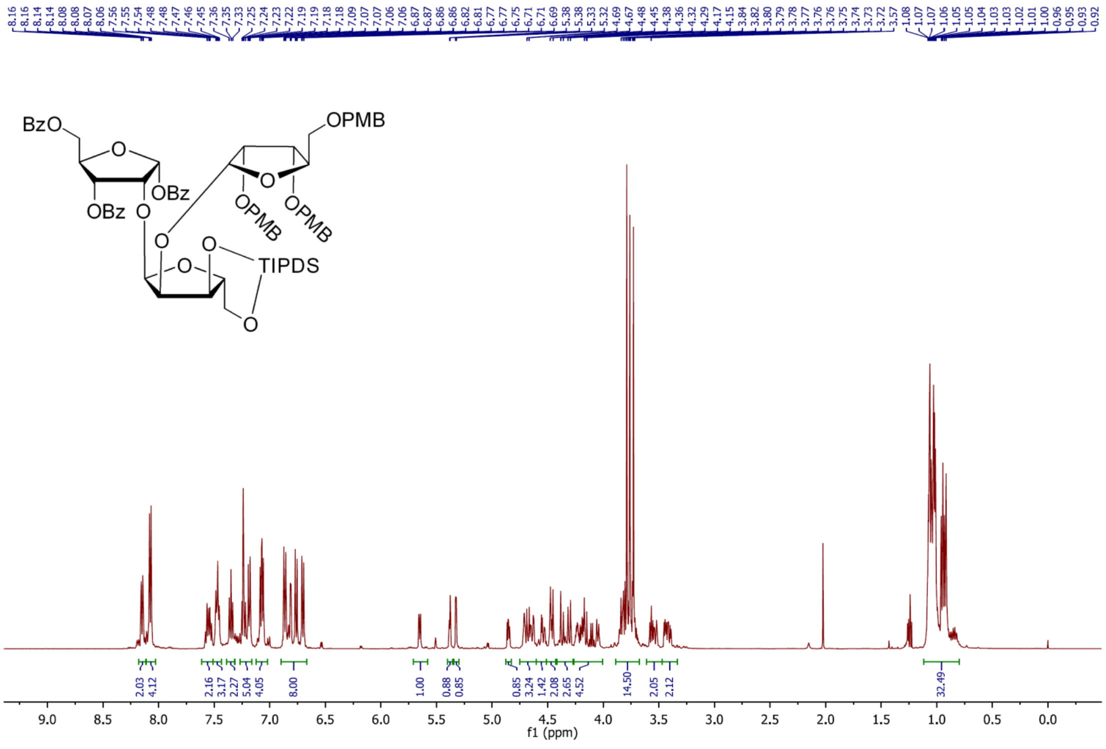

Supplementary figure 42: ^{1}H-NMR spectrum of compound **S8**.

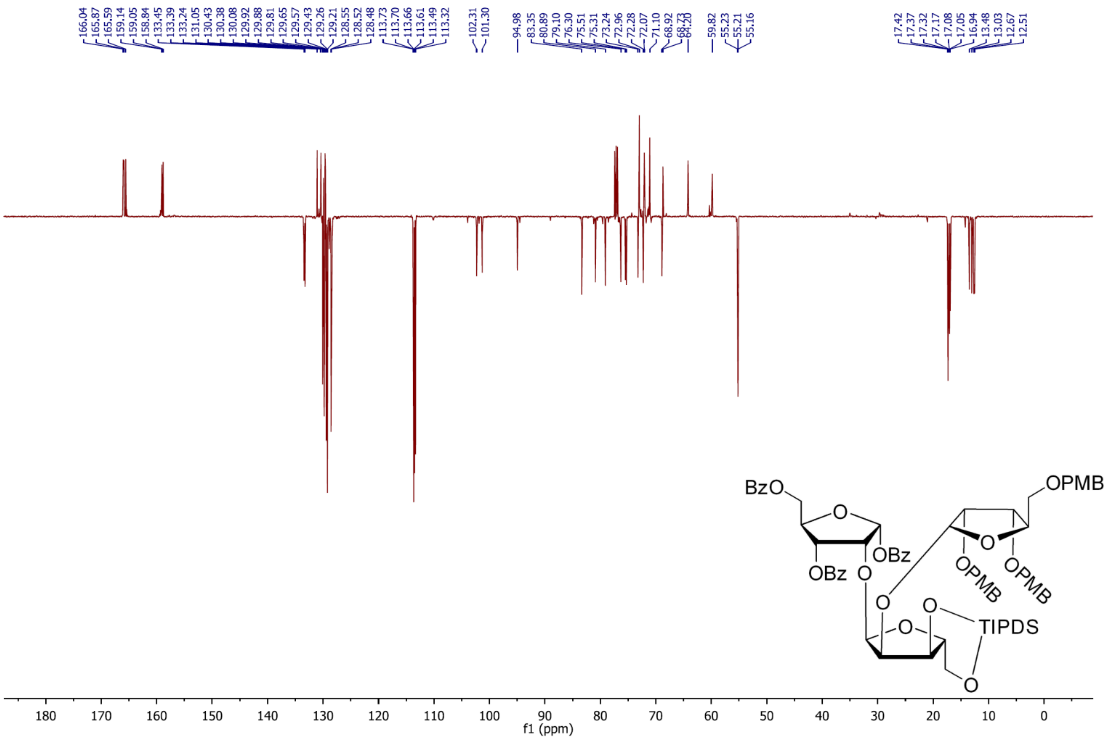

Supplementary figure 43: ^{13}C-NMR spectrum of compound **S8**.

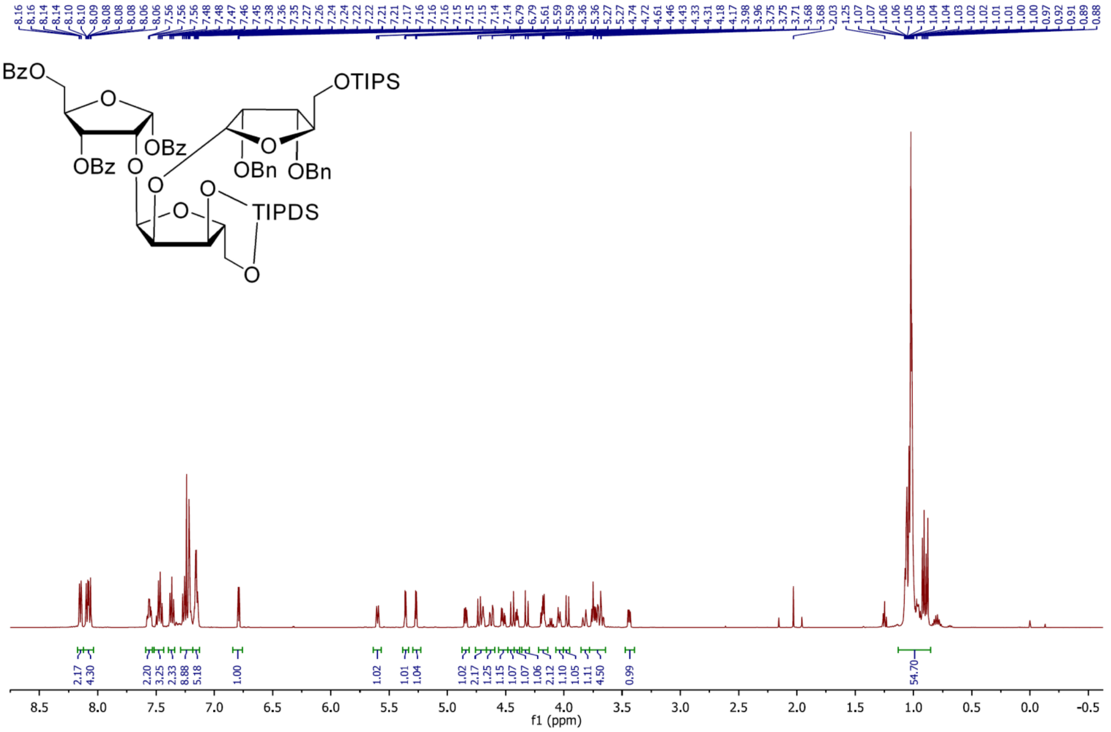

Supplementary figure 44: ^{1}H-NMR spectrum of compound **12**.

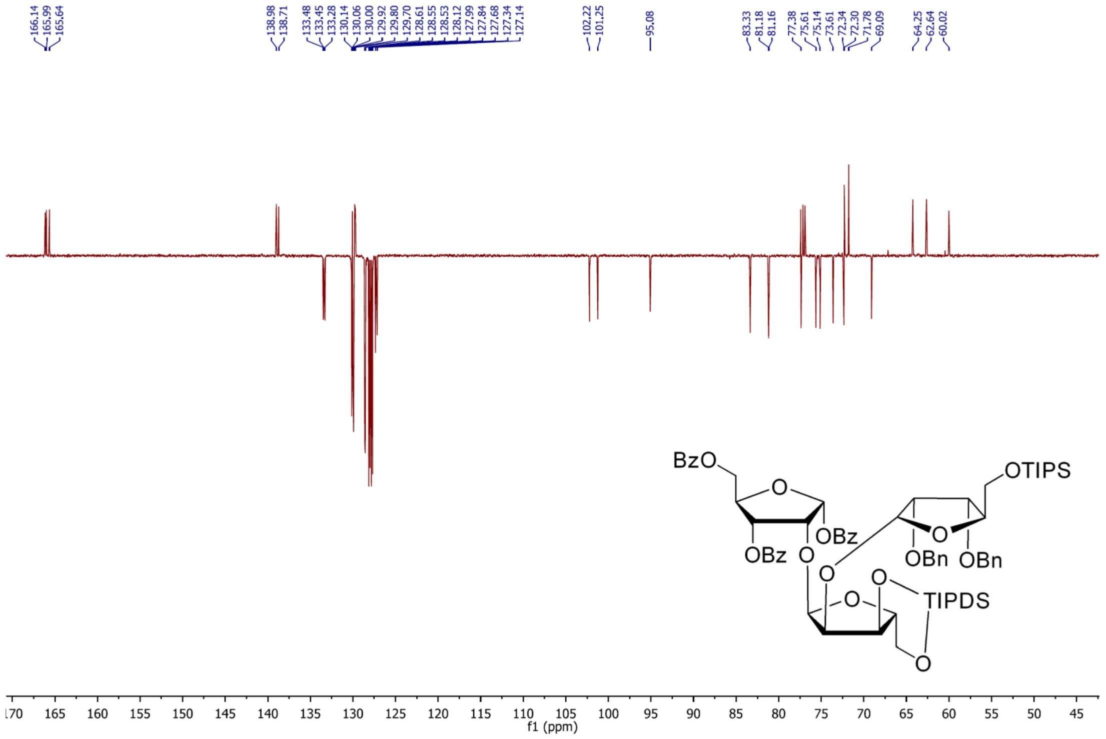

Supplementary figure 45: ^{13}C-NMR spectrum of compound **12**.

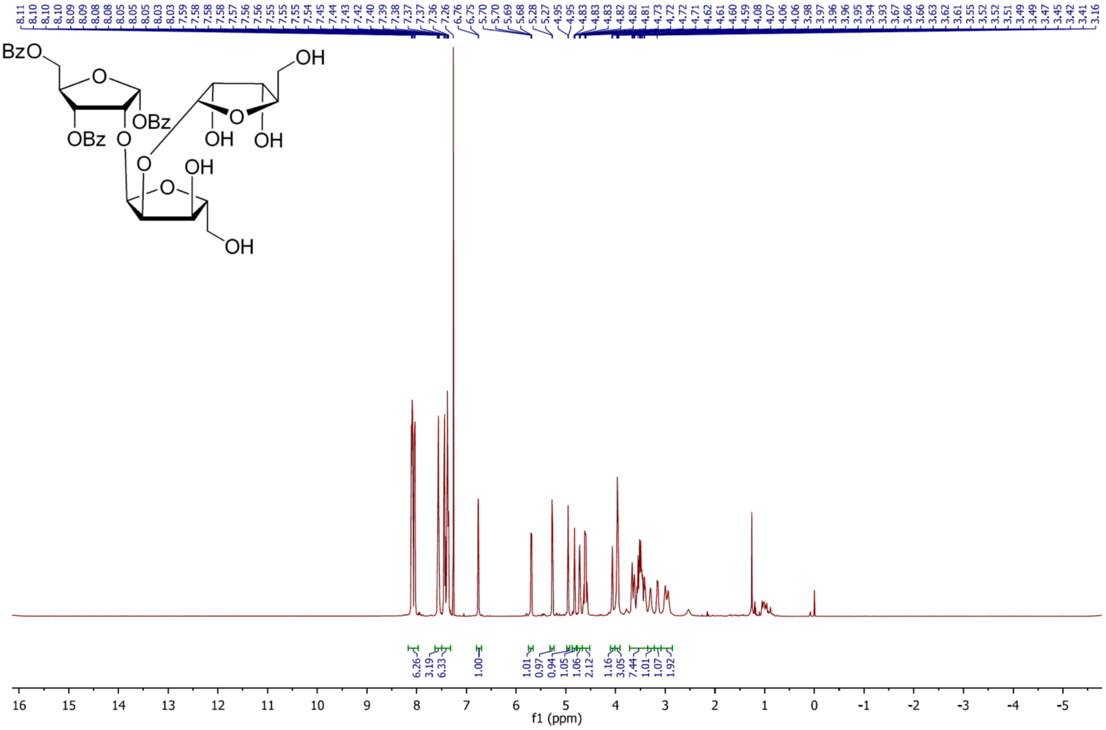

Supplementary figure 46: ^{1}H-NMR spectrum of compound **13**.

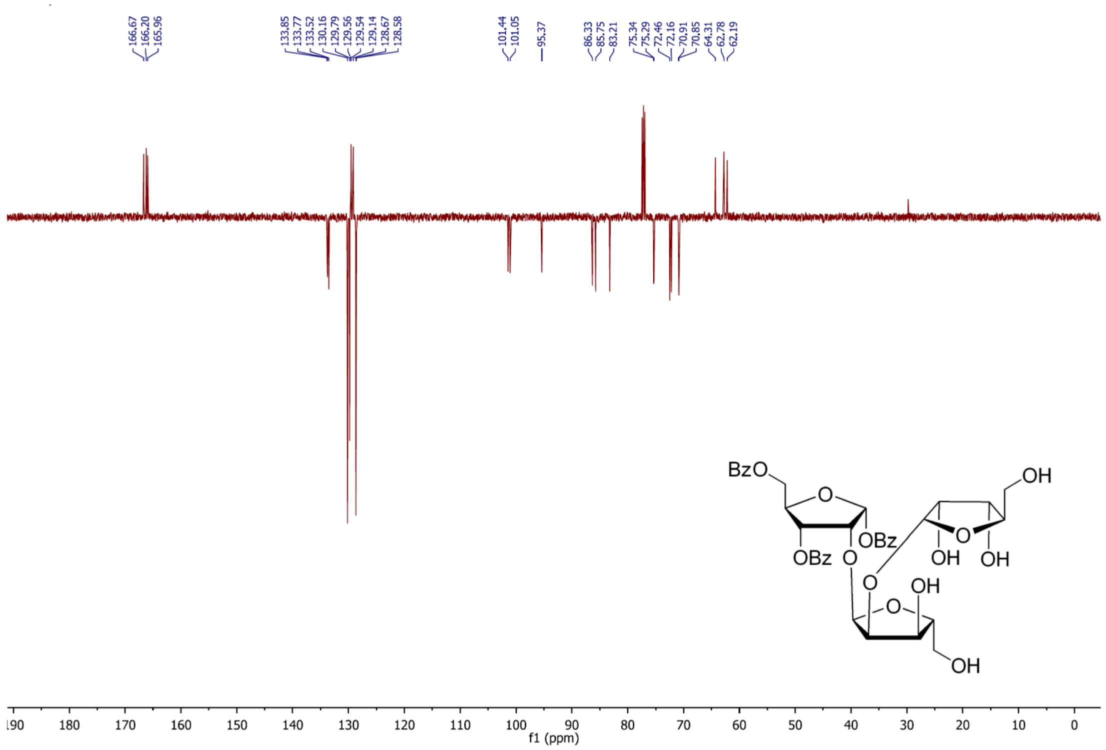

Supplementary figure 47: ^{13}C-NMR spectrum of compound **13**.

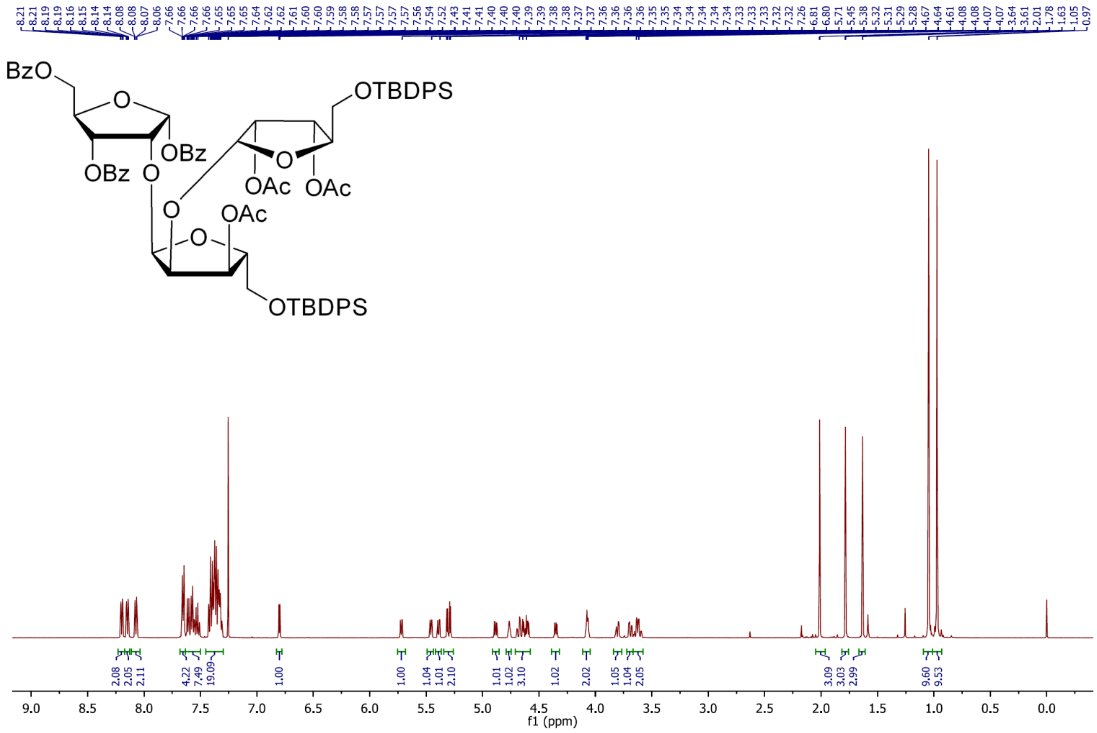

Supplementary figure 48: ^{1}H-NMR spectrum of compound **14**.

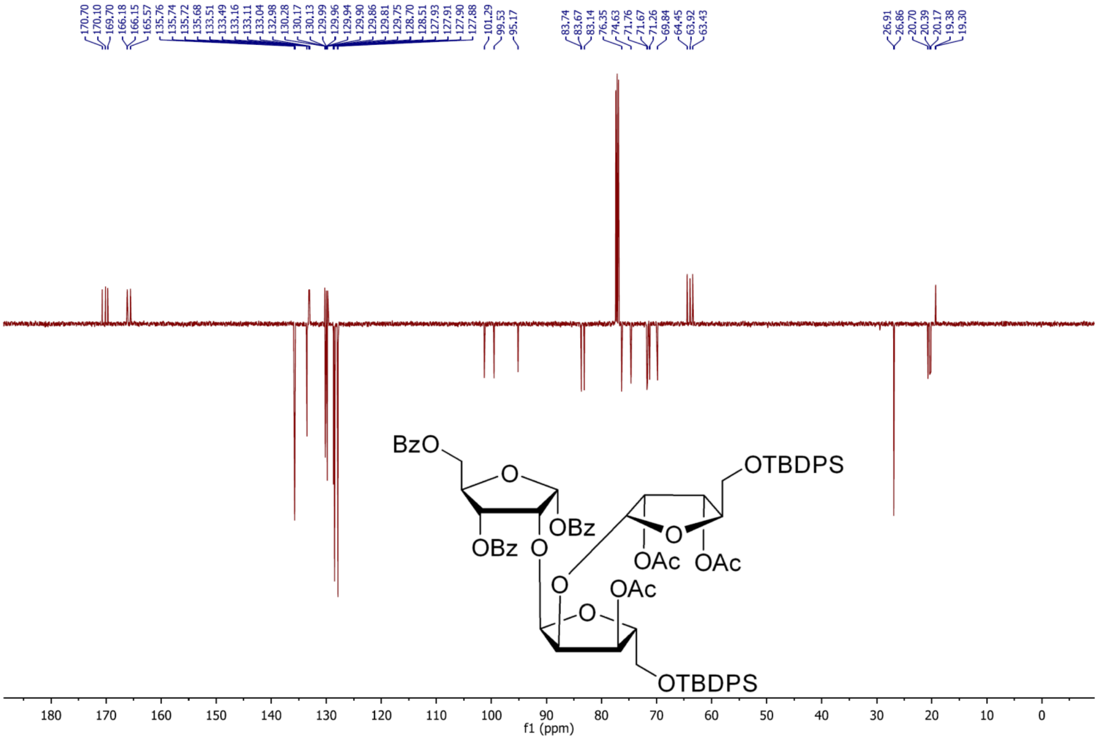

Supplementary figure 49: ^{13}C-NMR spectrum of compound **14**.

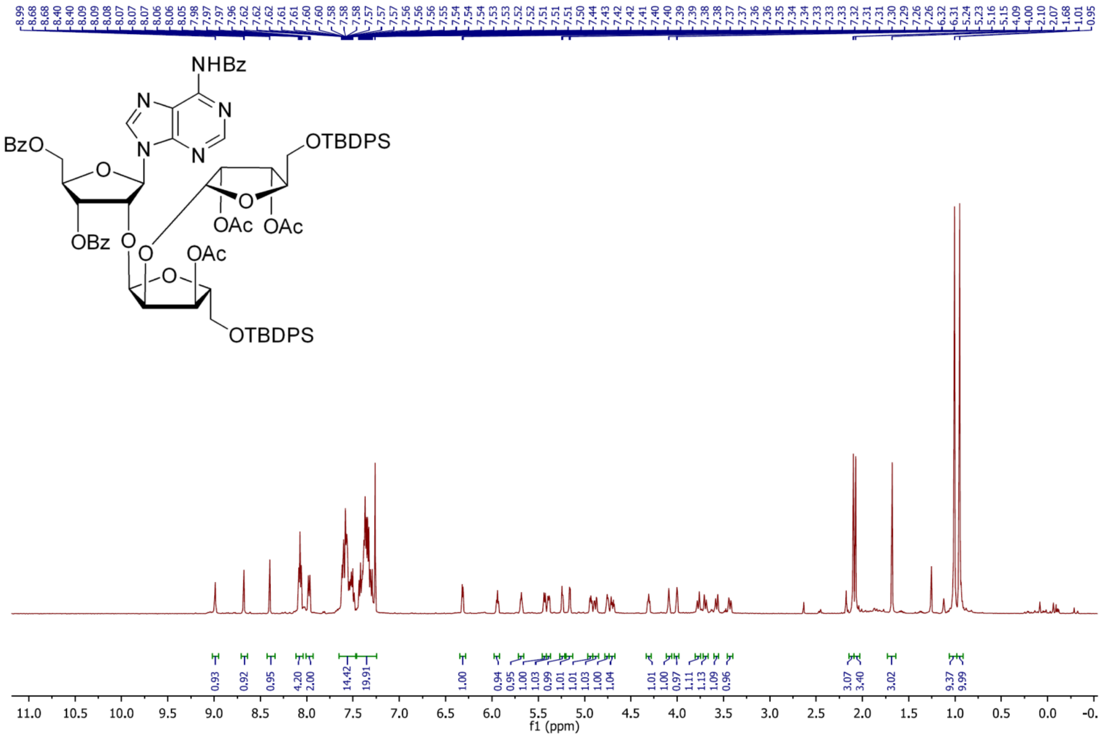

Supplementary figure 50: ^{1}H-NMR spectrum of compound **15**.

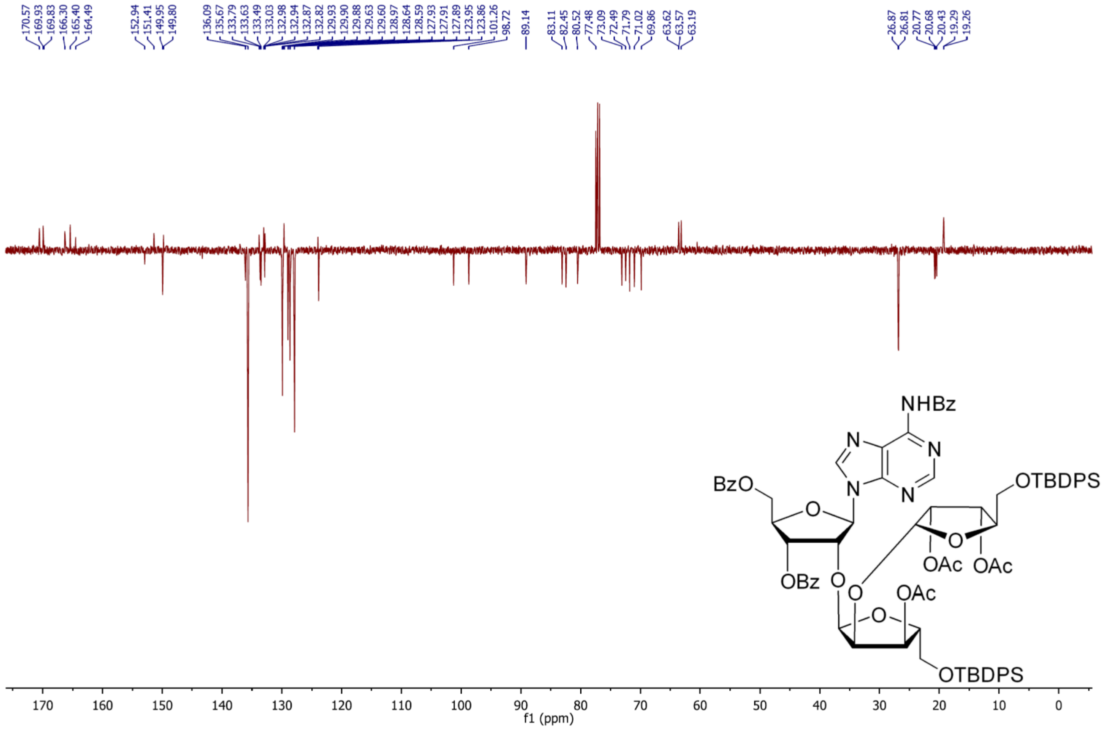

Supplementary figure 51: ^{13}C-NMR spectrum of compound **15**.

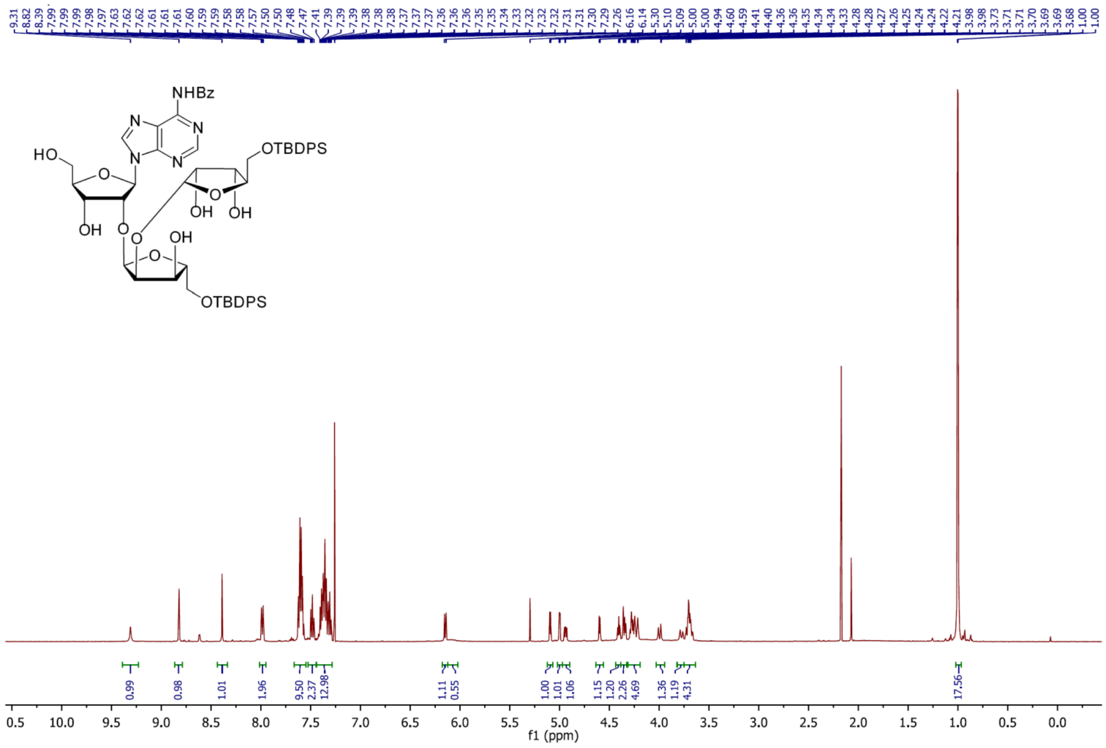

Supplementary figure 52: ^{1}H-NMR spectrum of compound **16**.

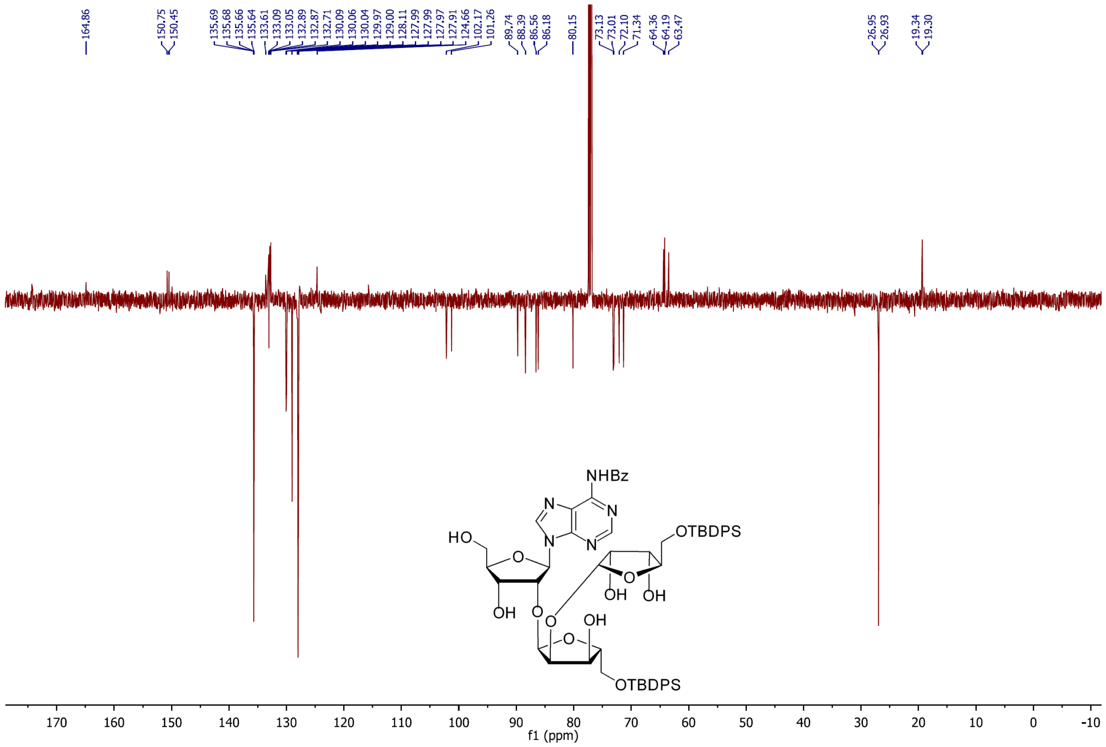

Supplementary figure 53: ^{13}C-NMR spectrum of compound **16**.

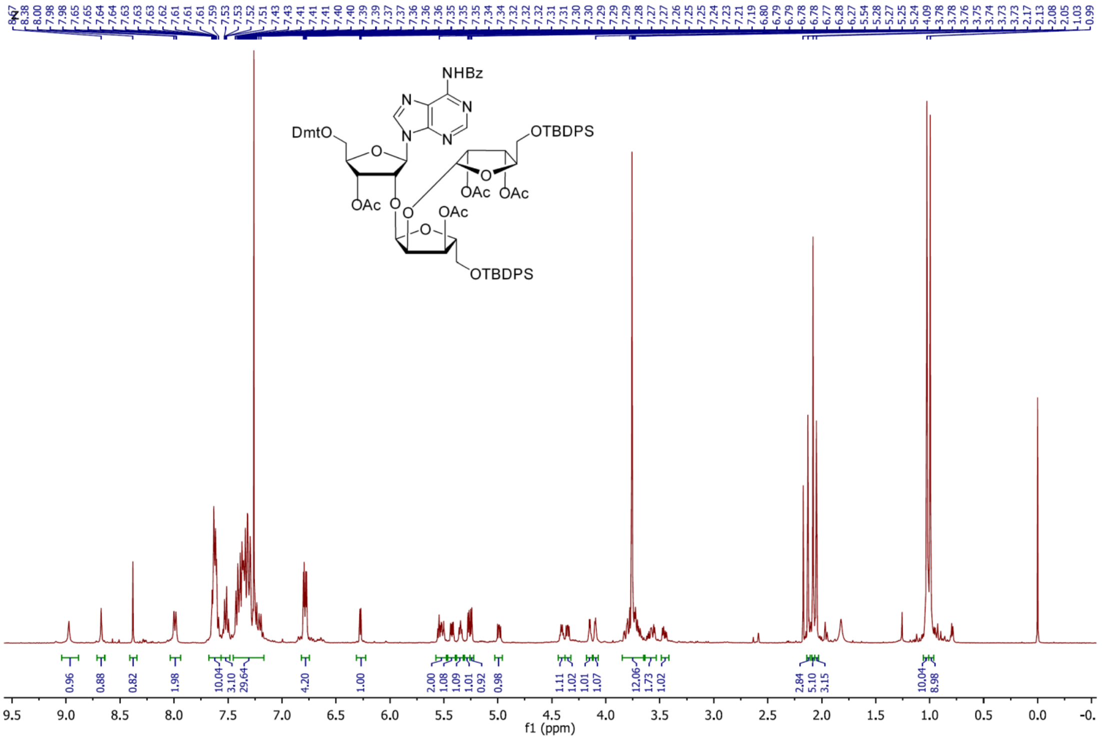

Supplementary figure 54: ^{1}H-NMR spectrum of compound **17**.

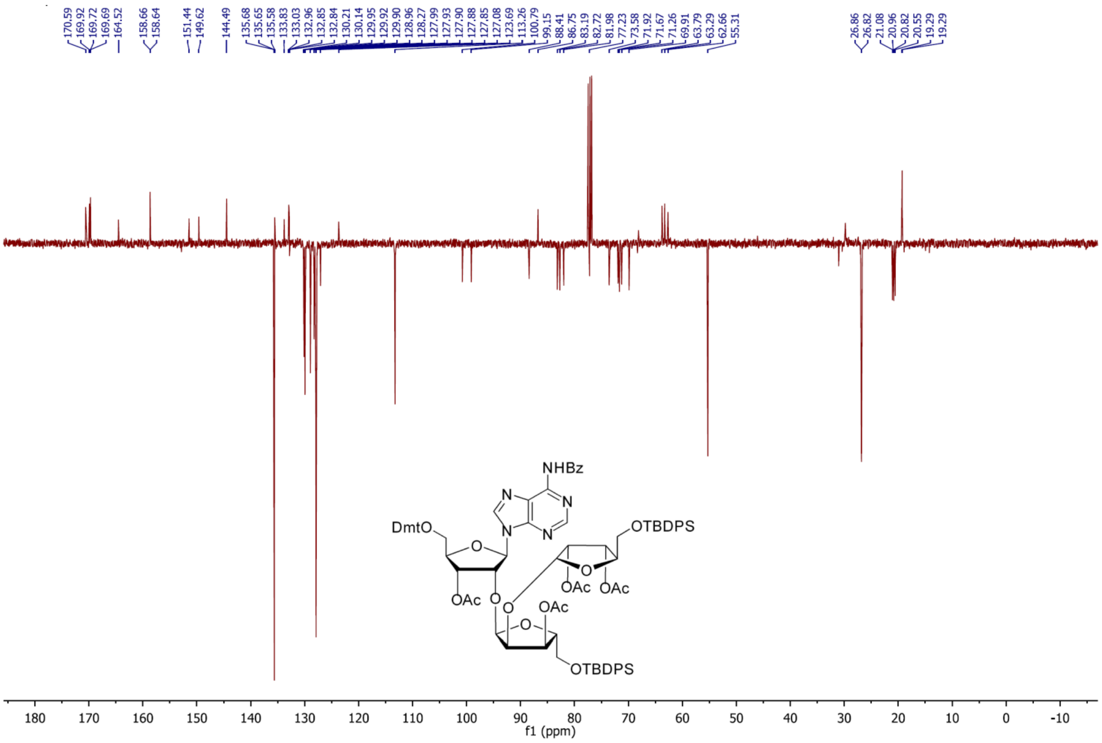

Supplementary figure 55: ^{13}C-NMR spectrum of compound **17**.

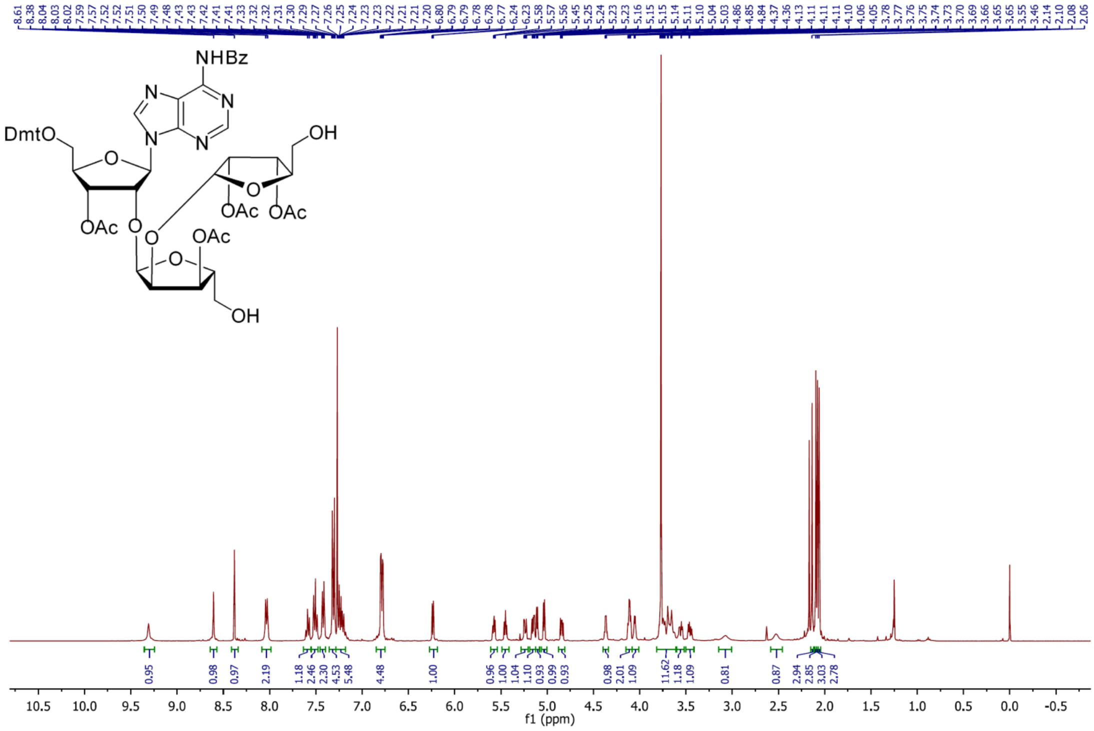

Supplementary figure 56: ^{1}H-NMR spectrum of compound **18**.

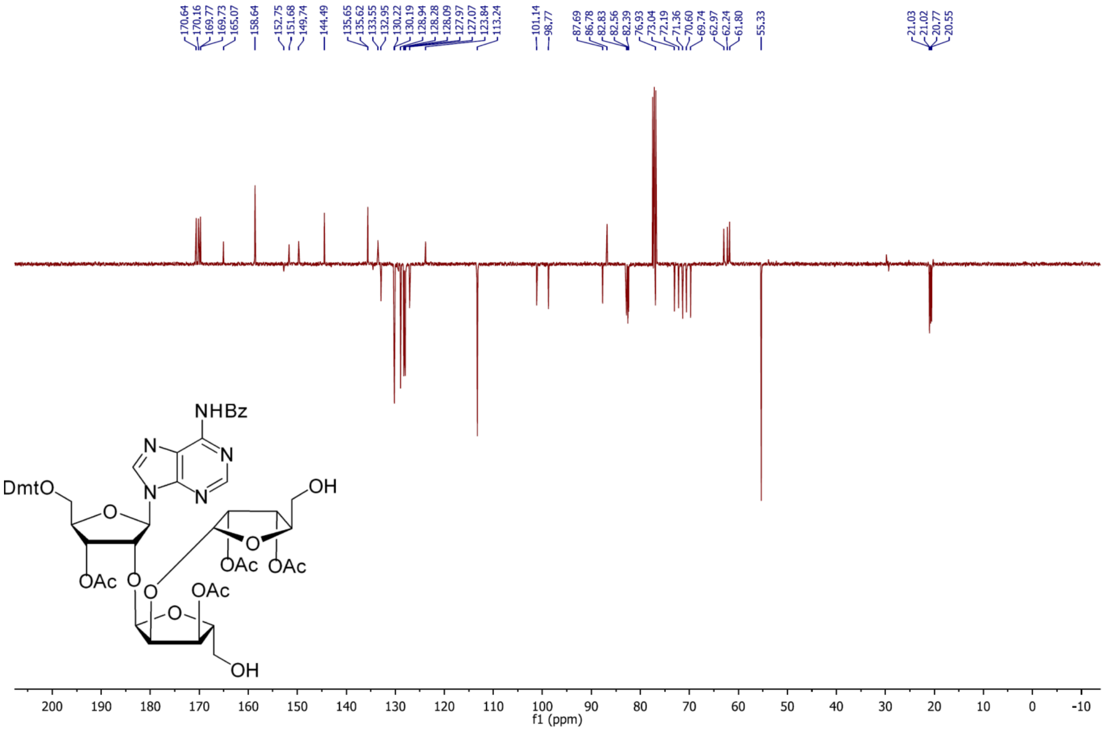

Supplementary figure 57: ^{13}C-NMR spectrum of compound **18**.

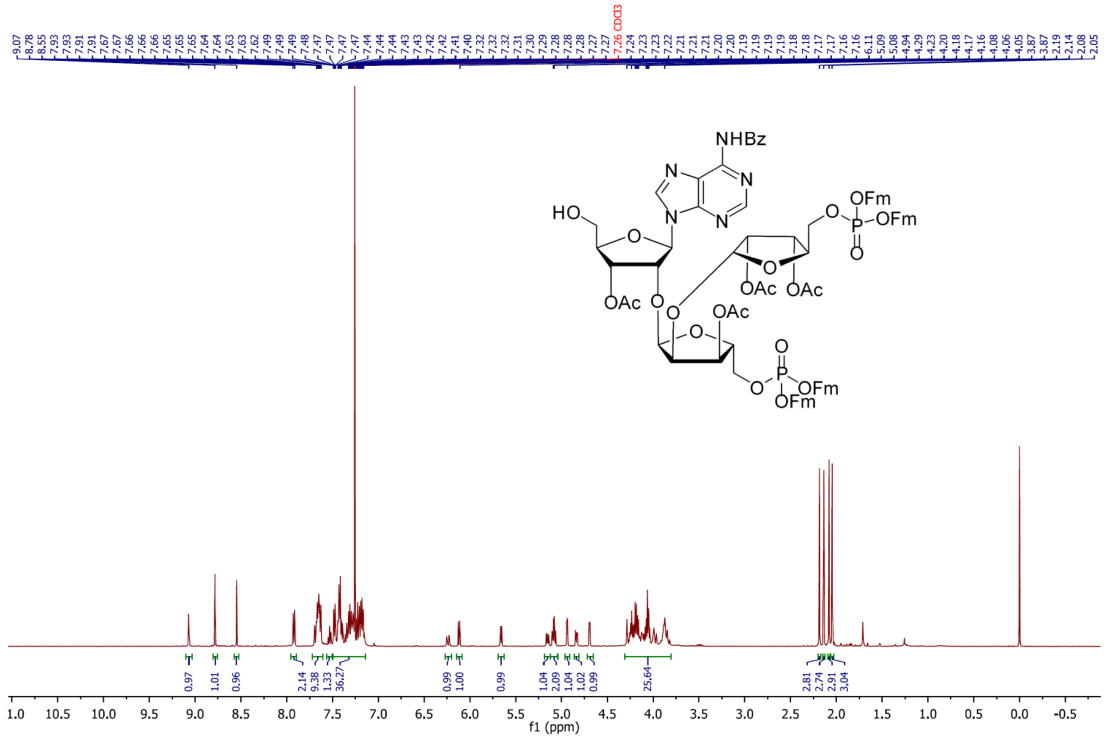

Supplementary figure 58: ^{1}H-NMR spectrum of compound **20**.

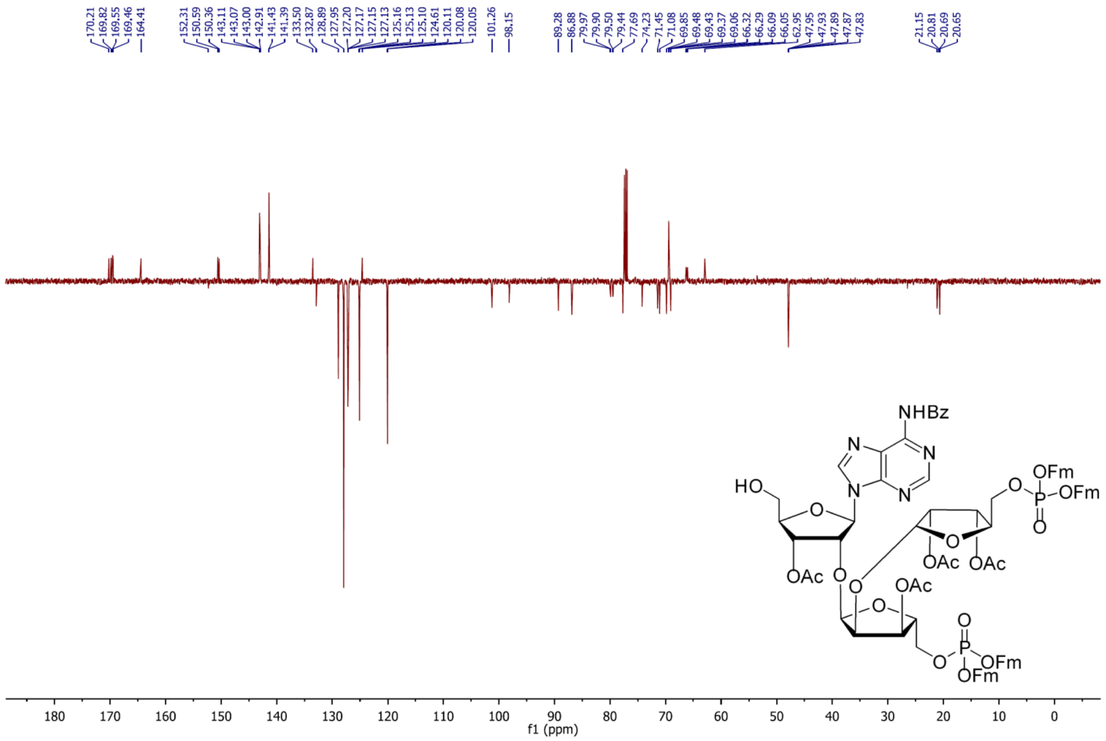

Supplementary figure 59: ^{13}C-NMR spectrum of compound **20**.

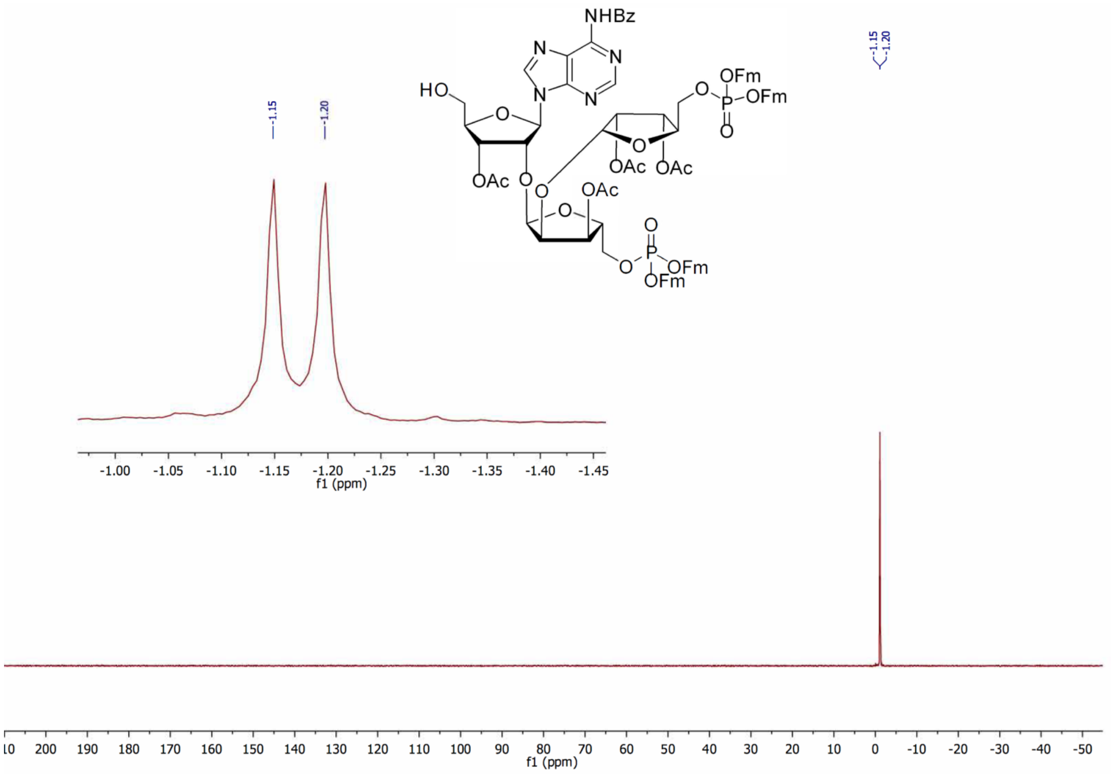

Supplementary figure 60: ^{31}P-NMR spectrum of compound **20**.

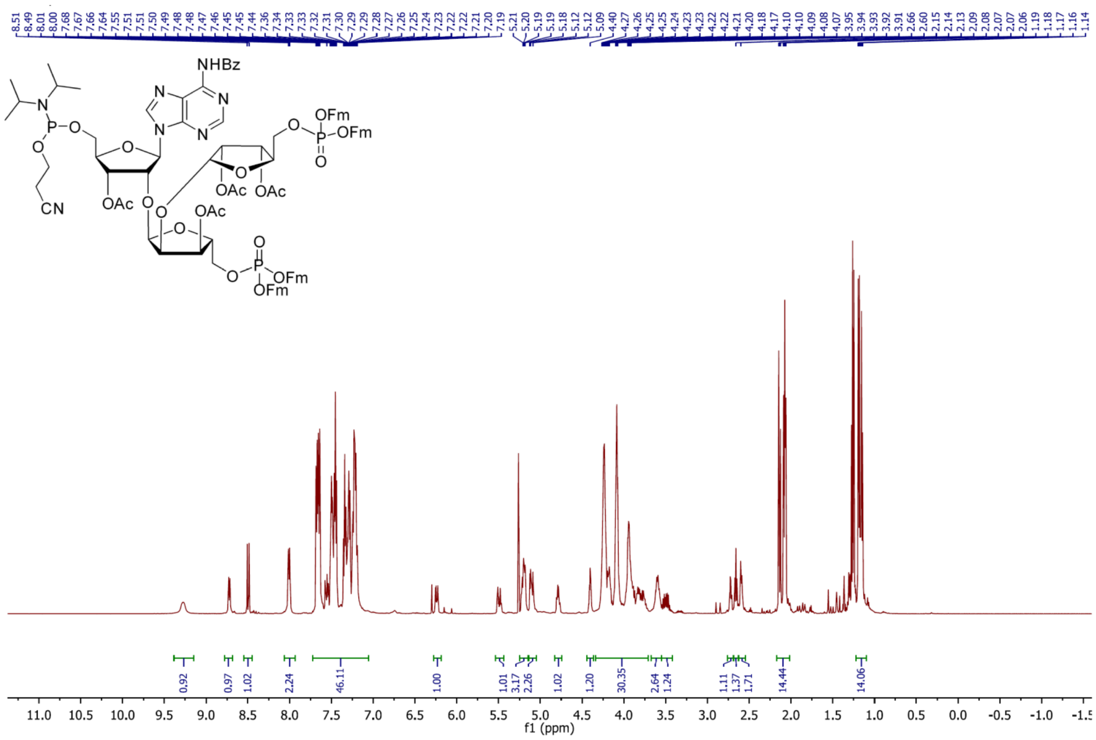

Supplementary figure 61: ^{1}H-NMR spectrum of compound **3**.

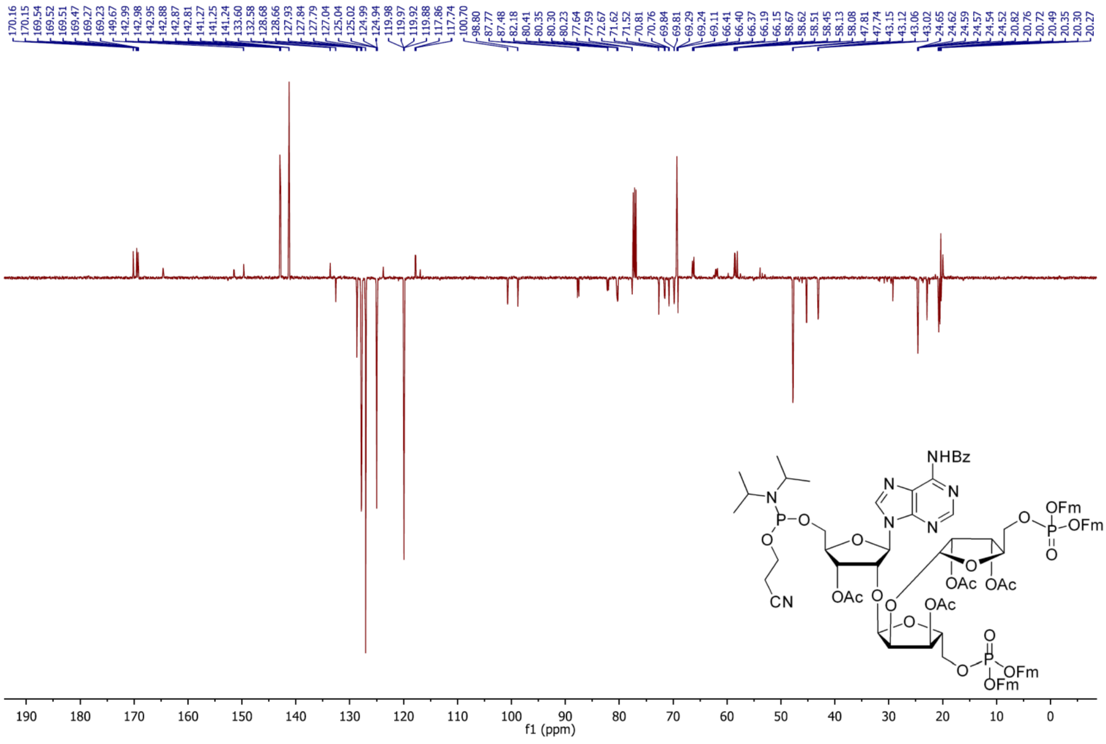

Supplementary figure 62: ^{13}C-NMR spectrum of compound **3**.

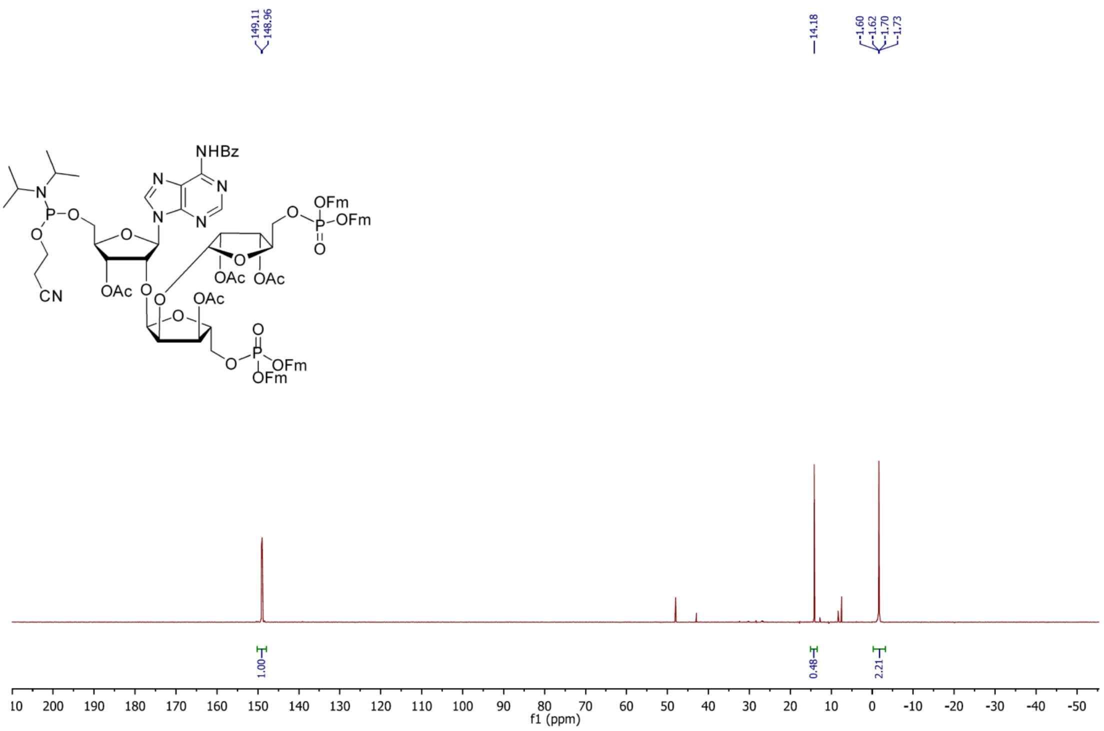

Supplementary figure 63: ^{31}P-NMR spectrum of compound **3**.

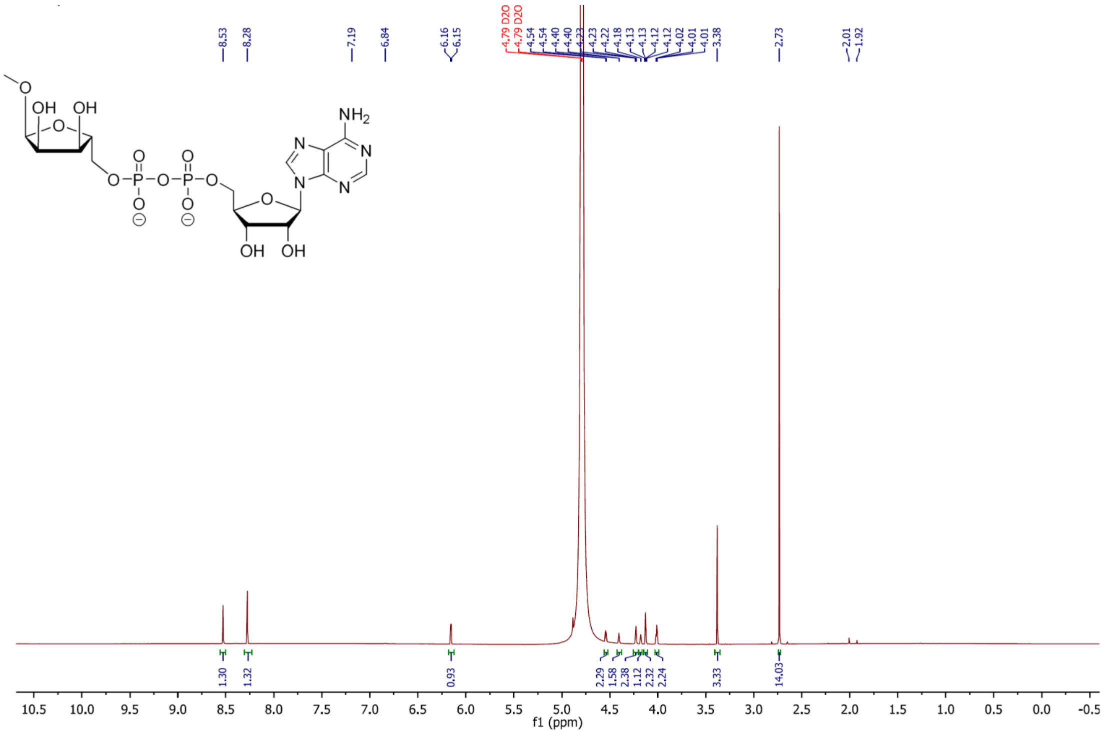

Supplementary figure 64: ^{1}H-NMR spectrum of compound **23**.

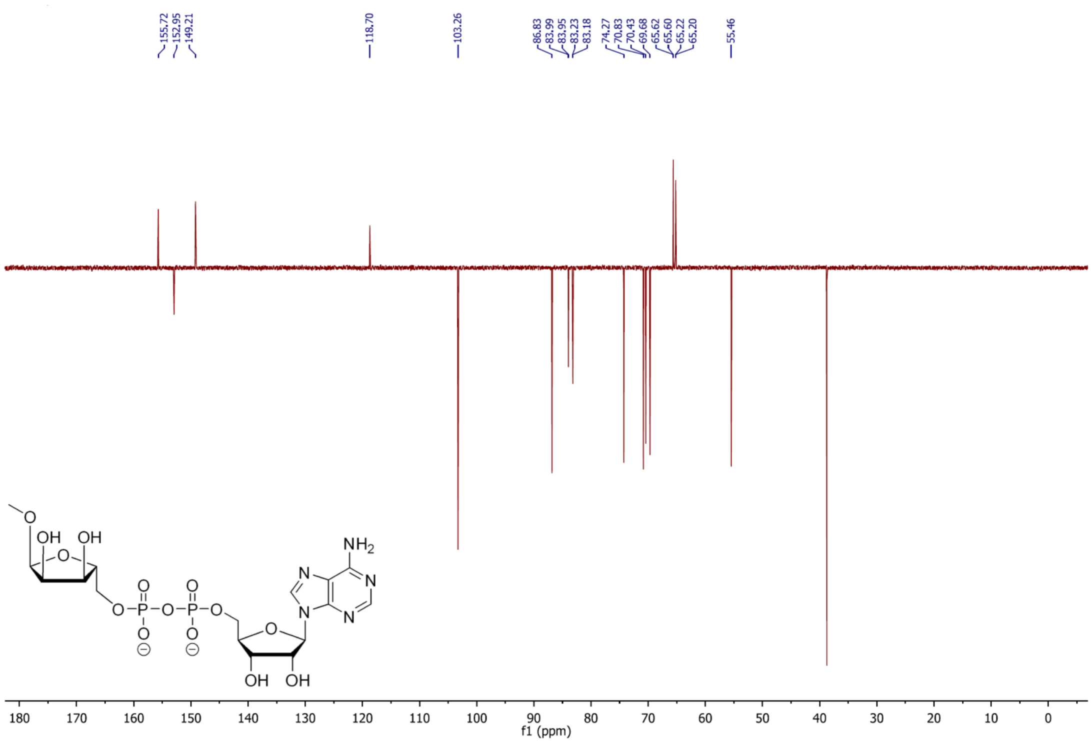

Supplementary figure 65: ^{13}C-NMR spectrum of compound **23**.

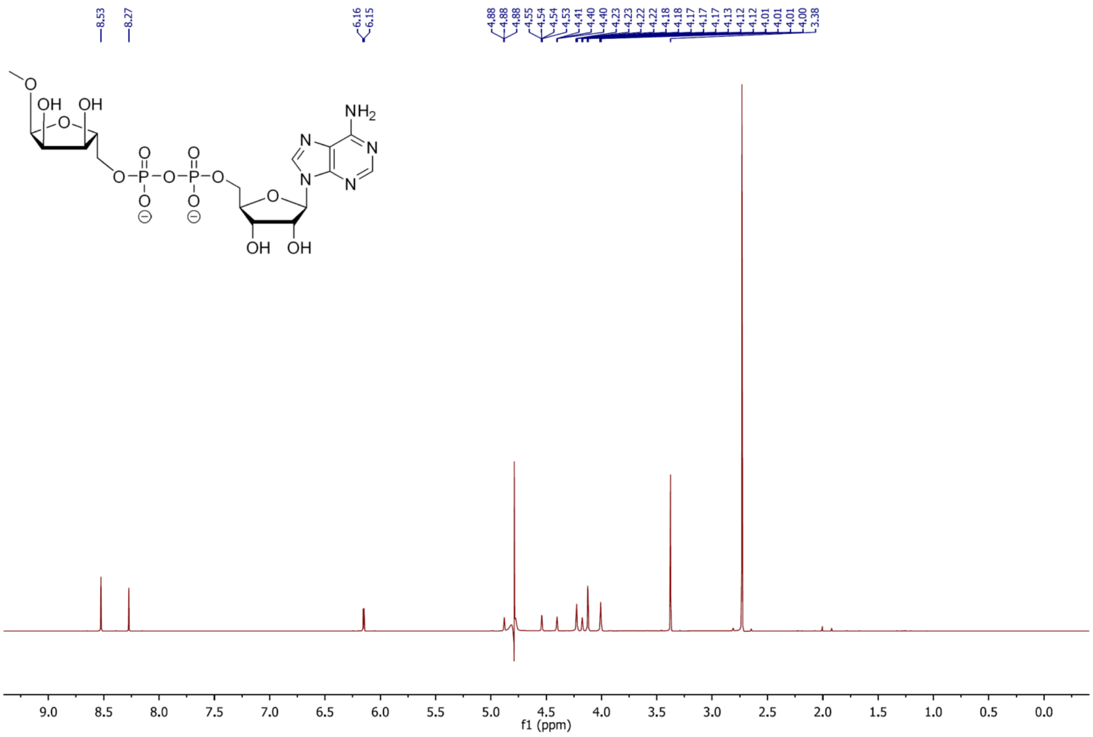

Supplementary figure 66: ^{1}H-NMR spectrum (presat) of compound **23**.

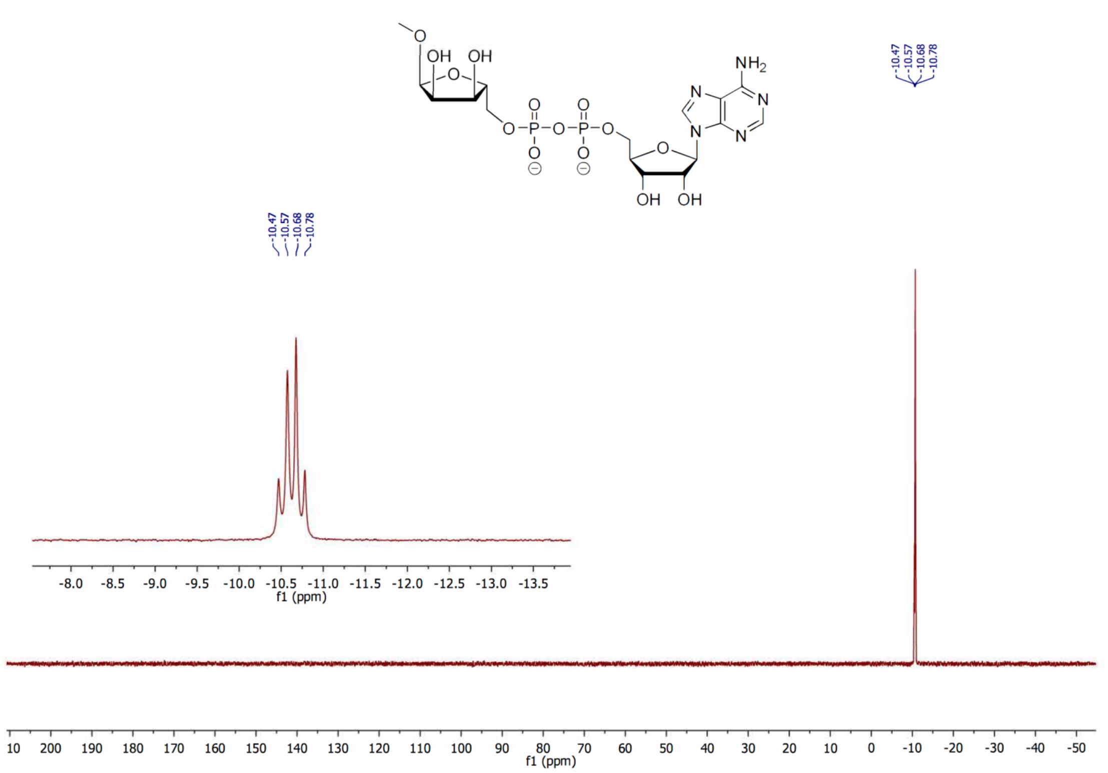

Supplementary figure 67: ^{31}P-NMR spectrum of compound **23**.

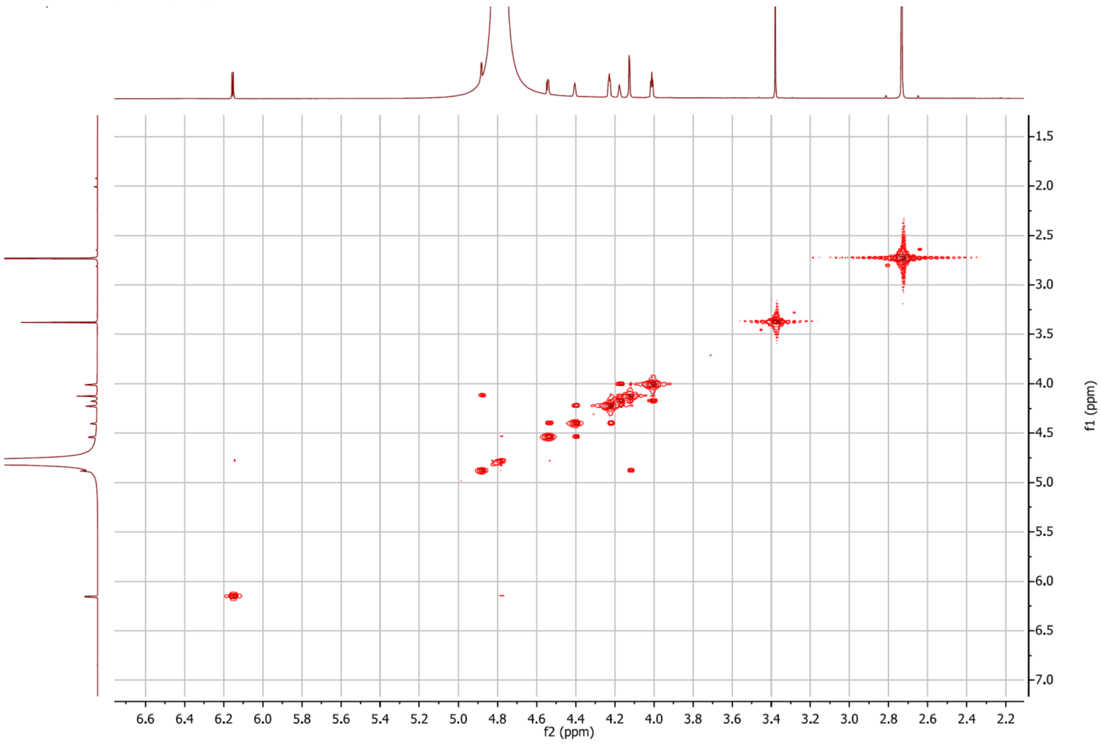

Supplementary figure 68: COSY spectrum of compound **S1**.

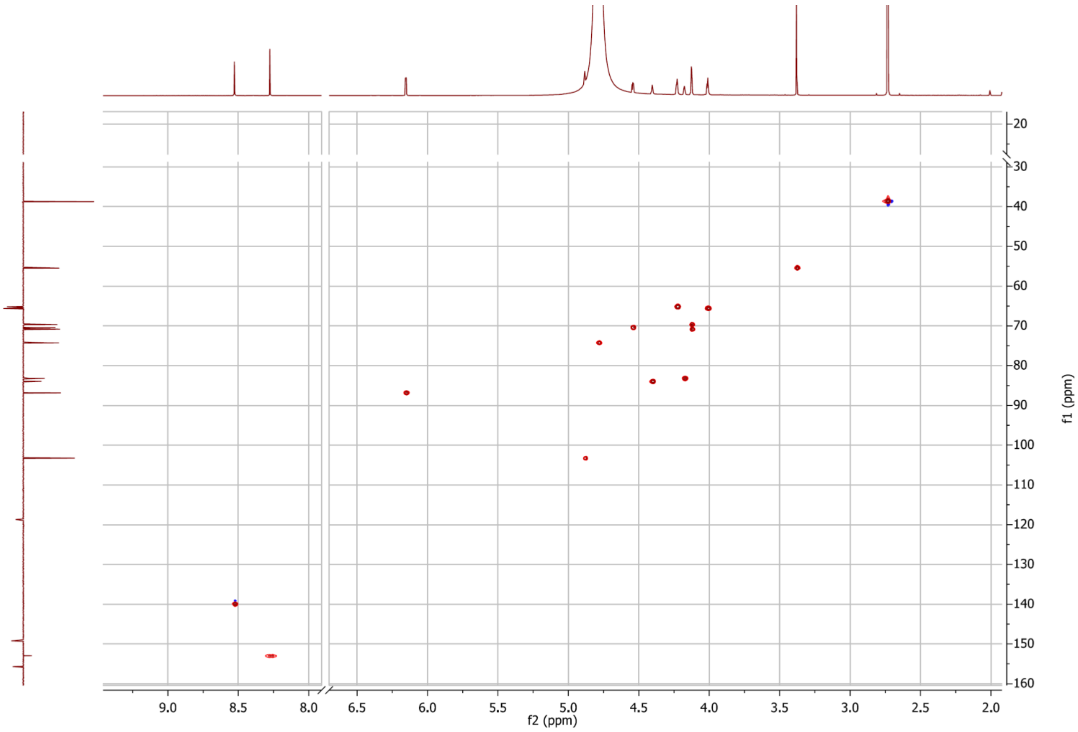

Supplementary figure 69: HSQC spectrum of compound **23**.

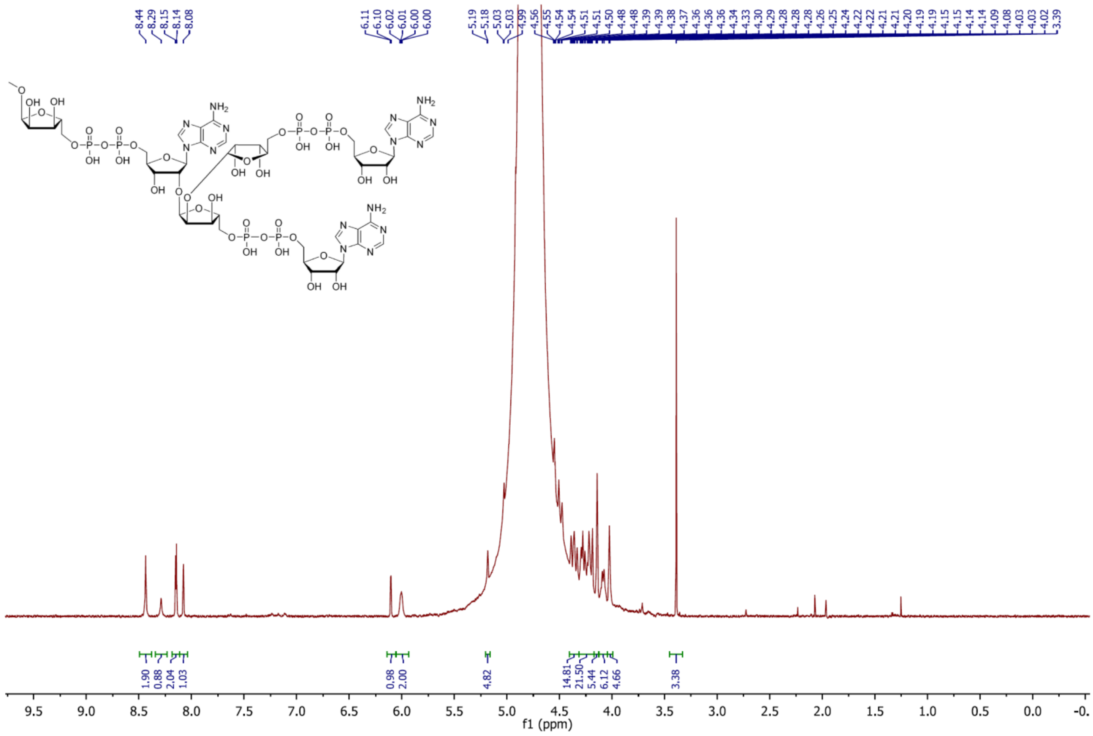

Supplementary figure 70: ^{1}H-NMR spectrum of compound **1**.

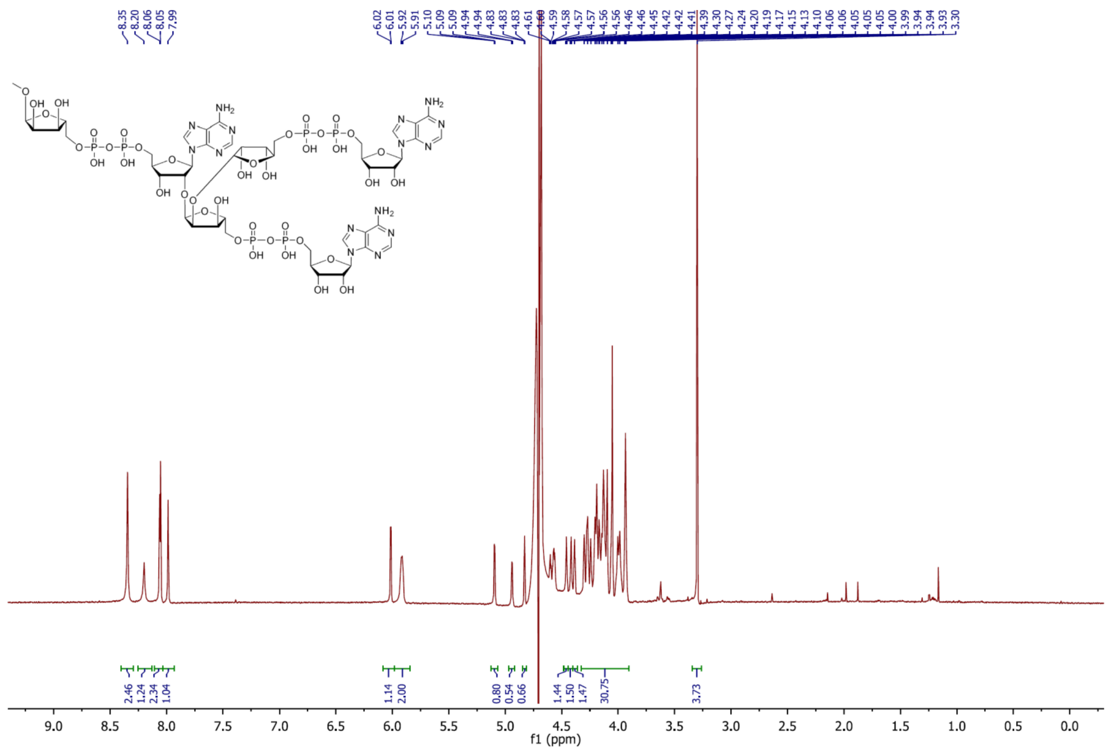

Supplementary figure 71: ^{1}H-NMR spectrum (presat) of compound **1**.

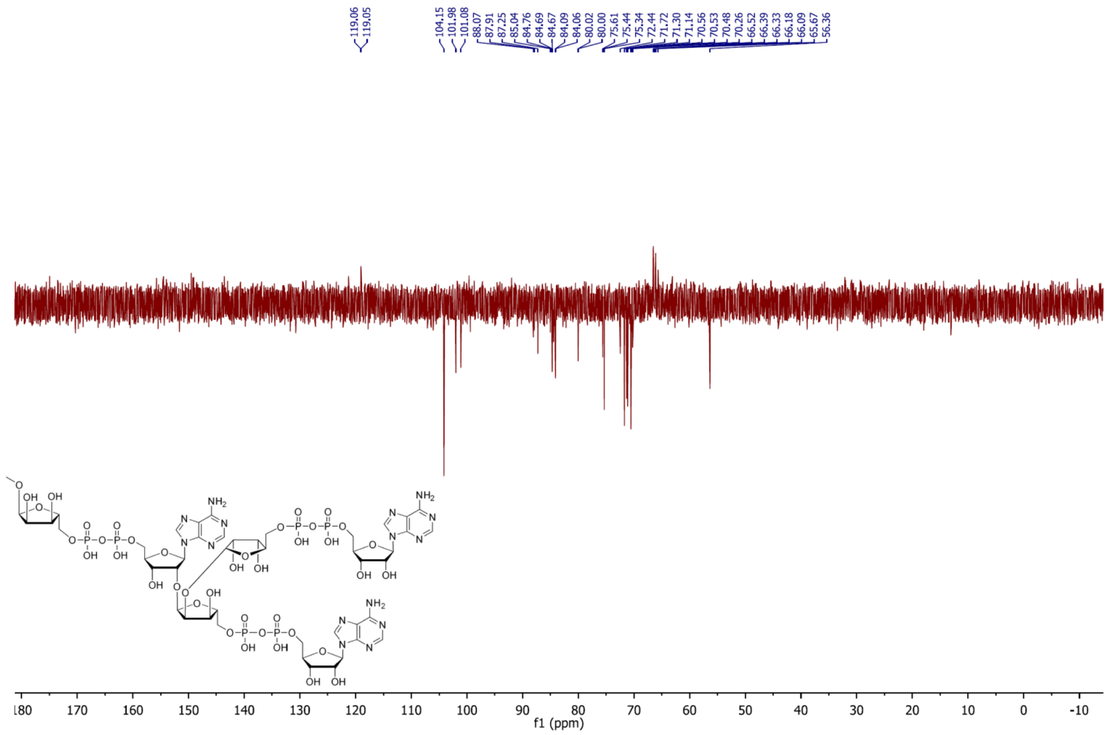

Supplementary figure 72: ^{13}C-NMR spectrum of compound **1**.

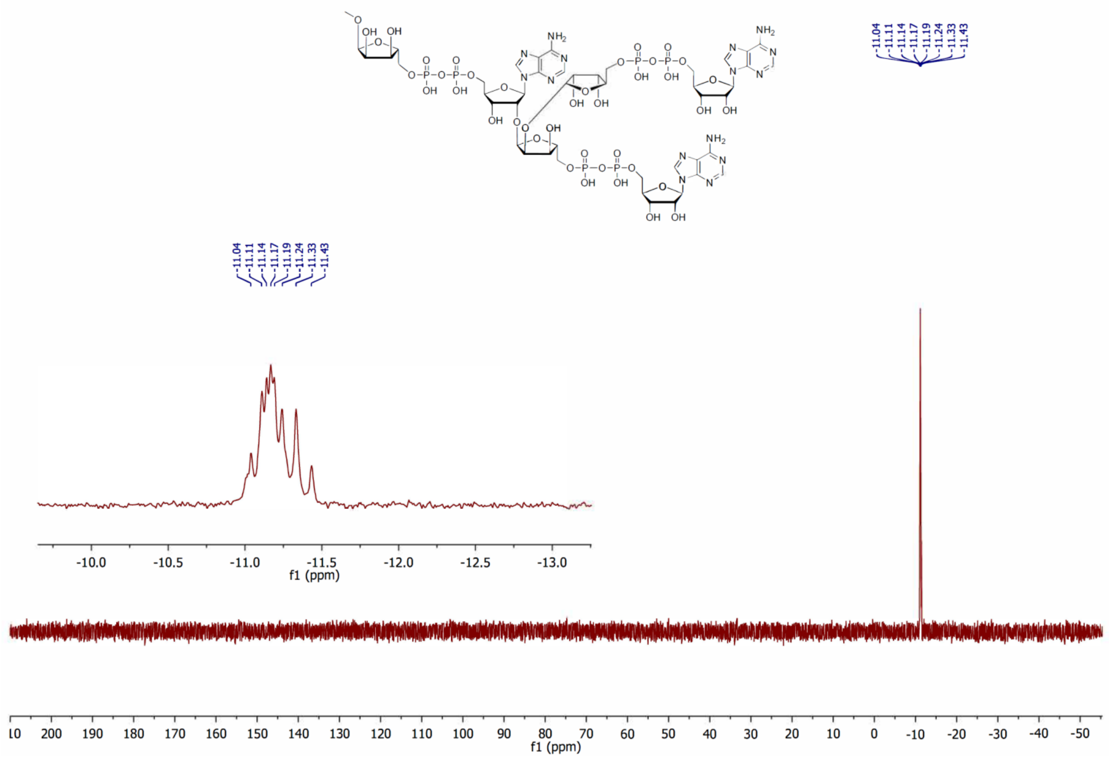

Supplementary figure 73: ^{31}P-NMR spectrum of compound **1**.

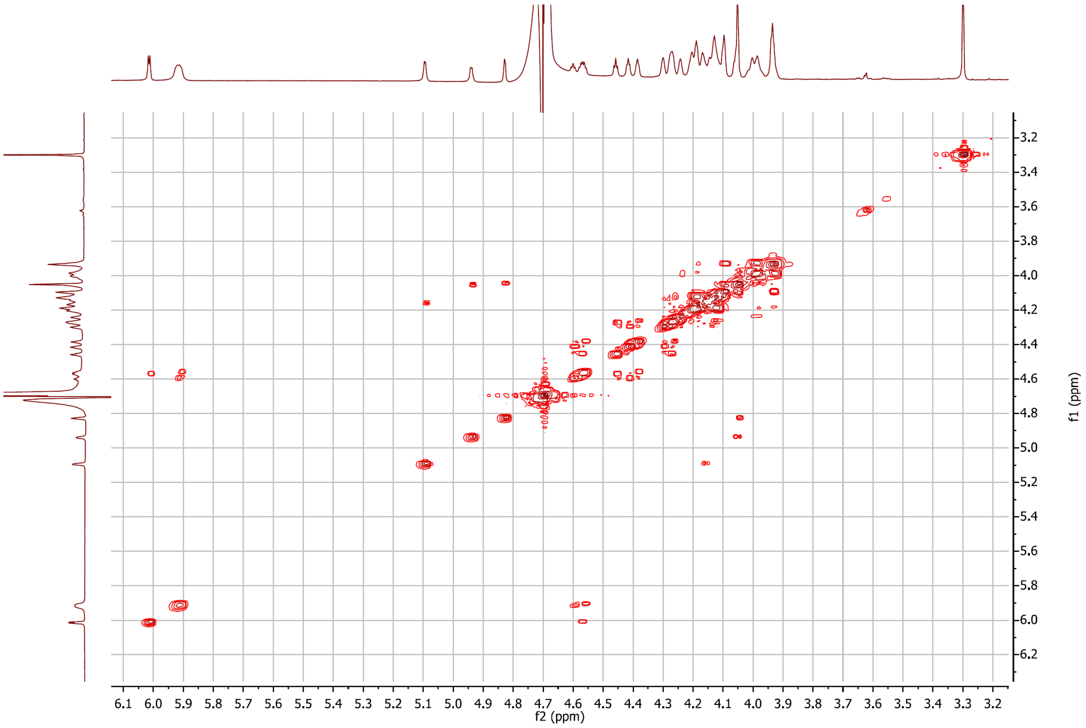

Supplementary figure 74: COSY spectrum of compound **1**.

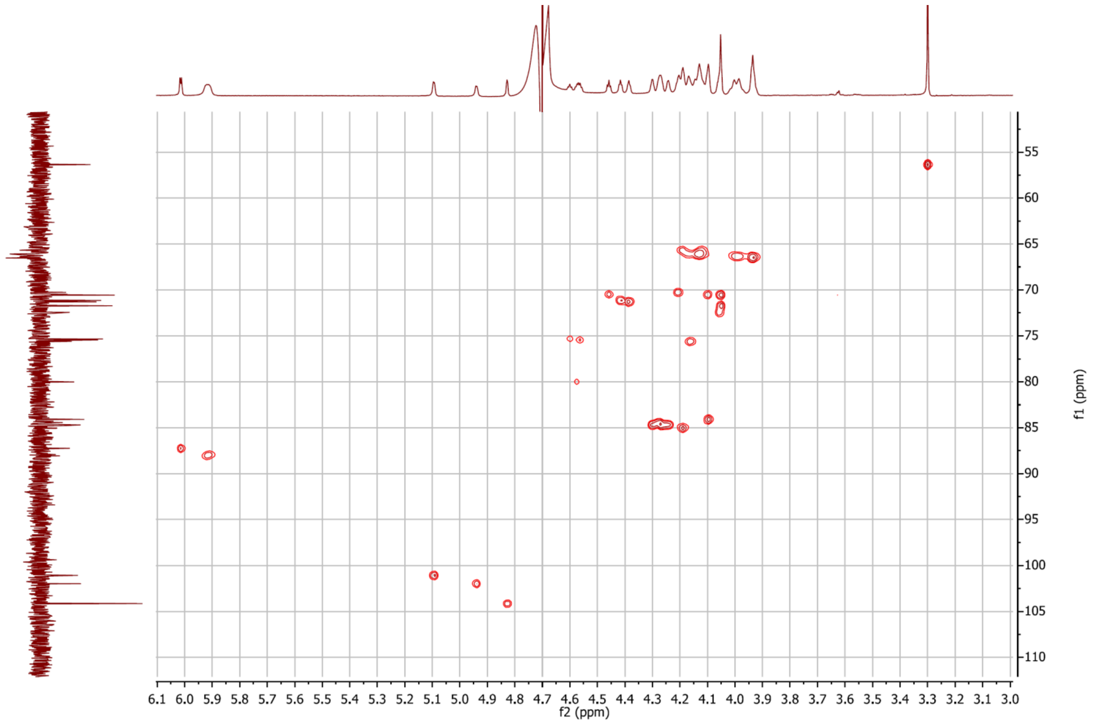

Supplementary figure 75: HSQC spectrum of compound **1**.

Supplementary References

1. Li, N.-S., Lu, J. & Piccirilli, J.A. Efficient Synthesis of Methyl 3,5-Di-O-benzyl-α-d-ribofuranoside and Application to the Synthesis of 2‘-C-β-Alkoxymethyluridines. *Organic Letters* **9**, 3009-3012 (2007).
2. Bialy, L. & Waldmann, H. Total synthesis and biological evaluation of the protein phosphatase 2A inhibitor cytostatin and analogues. *Chemistry* **10**, 2759-80 (2004).
3. Watanabe, Y., Nakamura, T. & Mitsumoto, H. Protection of phosphate with the 9-fluorenylmethyl group. Synthesis of unsaturated-acyl phosphatidylinositol 4,5-bisphosphate. *Tetrahedron Letters* **38**, 7407-7410 (1997).
4. Finch, P., Iskander, G.M. & Siriwardena, A.H. Convenient syntheses of 2,3,5-tri-O-benzyl-arabino- and −ribofuranoses via their allyl glycosides. *Carbohydrate Research* **210**, 319-325 (1991).
5. Minakawa, N., Kato, Y., Uetake, K., Kaga, D. & Matsuda, A. An improved large scale synthesis of 1,4-anhydro-4-thio-d-ribitol. *Tetrahedron* **59**, 1699-1702 (2003).
6. Liu, Q., Kistemaker, H.A.V., Overkleeft, H.S., van der Marel, G.A. & Filippov, D.V. Synthesis of ribosyl-ribosyl-adenosine-5',5'',5'''(triphosphate)-the naturally occurring branched fragment of poly(ADP ribose). *Chem. Commun.* **53**, 10255-10258 (2017).
